# Synthesis of bodinieric acids A and B, both C-18 and C-19-functionalized abietane diterpenoids: DFT study of the key aldol reaction†‡

**DOI:** 10.1039/d0ra02711a

**Published:** 2020-04-16

**Authors:** Ramón J. Zaragozá, Miguel A. González-Cardenete

**Affiliations:** Departamento de Química Orgánica, Universidad de Valencia Dr. Moliner 50, 46100 Burjassot Valencia Spain; Instituto de Tecnología Química (UPV-CSIC), Universitat Politècnica de Valencia-Consejo Superior de Investigaciones Científicas Avda de los Naranjos s/n 46022 Valencia Spain migoncar@itq.upv.es

## Abstract

The first synthesis of C-18- and C-19-bifunctionalized abietane diterpenoids, bodinieric (or callicapoic) acids, *via* an aldol reaction has been developed. This key aldol reaction was very sensitive to steric hindrance. This fact has been studied by deuterium exchange experiments and DFT methods. Optimization of this reaction led to the synthesis of anti-inflammatory bodinieric acids A and B, starting from abietic acid.

## Abstract 2

The first synthesis of C-18- and C-19-bifunctionalized abietane diterpenoids, bodinieric (or callicapoic) acids, *via* an aldol reaction has been developed.

## Graphical abstract

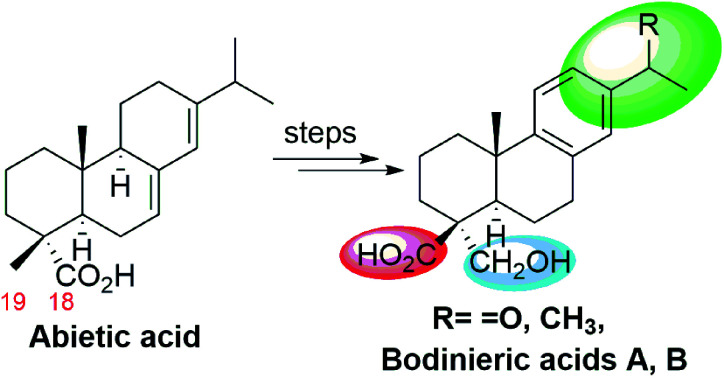

## Introduction

**Fig. 1:**
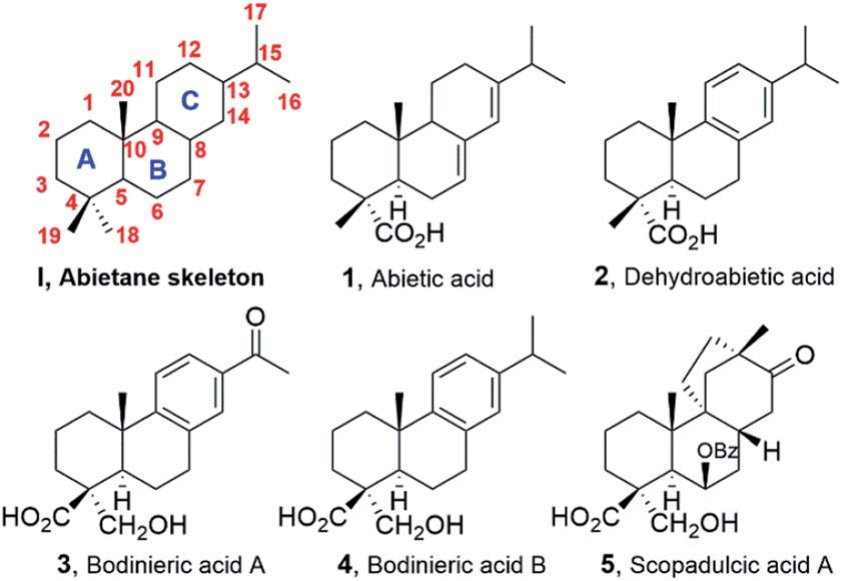
Abietane numbering system and some examples.

Diterpenoids of the abietane class have been known for almost 200 years since the discovery of abietic acid (1, Fig. 1) but new members are still being discovered and useful pharmacological applications are under study.^1^ From the organic synthesis point of view, the preparation of abietanes has long been a matter of interesting research.^2^ The abietane carbon framework characterized by a tricyclic ring system (Fig. 1) with several stereocenters and a wide grade of oxygenation pattern on the skeleton invites synthetic chemists to be creative and efficient. The recent report, in 2018, on isolation of related abietane congeners of dehydroabietic acid (2, DHA) with both C-18- and C-19-oxygenated carbons, such as bodinieric acid A (3, deacetylcallicapoic acid M5), and B (4, also known as callicapoic acid M4) attracted our attention.^3^ Their important biological activities as a selective spleen tyrosine kinase (SYK) inhibitors have led to a patent.^4^ These natural products reminded us an old synthetic work on another diterpenoid, possessing exactly the same stereochemistry at C4 and rare constitution, that is, both C18 and C19 oxidized and with the same oxidation pattern at this carbons in ring A, (−)-scopadulcic acid A (5, Fig. 1), as we had worked in the field 20 years ago.^5^ We became interested in developing a scalable synthetic route towards those acids for further study, including the control of the stereoselectivity at the quaternary stereocenter at C-4. Herein, we describe the first synthesis of bodinieric acids A (3) and B (4) through a key aldol reaction of which we give further insight based on deuterium exchange experiments and DFT computational studies.

## Results and discussion

**Scheme 1:**
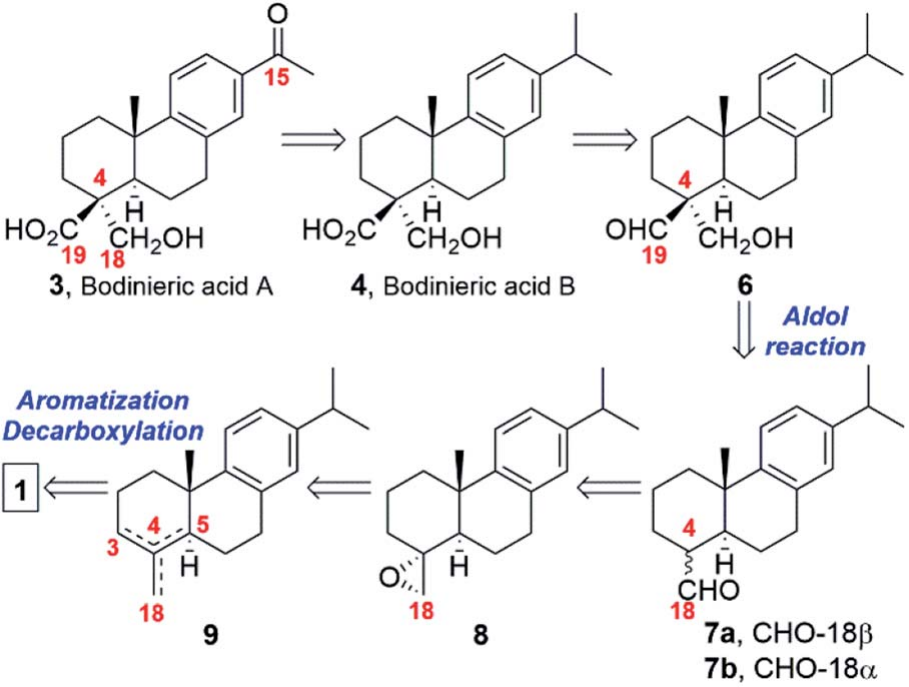
Retrosynthetic Analysis.

The original retrosynthetic analysis is outlined in Scheme 1. In a similar approach to the work of Ziegler and Wallace to complete the synthesis of (±)-scopadulcic acid A in 1995,^6^ we envisaged that bodinieric acid B (4) could be readily obtained from the aldol 6 (Scheme 1) by Pinnick oxidation. Conversion of the isopropyl moiety into a methyl ketone moiety would then afford bodinieric acid A (3). Aldol 6 would be prepared by treatment with formalin and base of a mixture of known aldehydes 7a,b used in the synthesis of callitrisic acid (4-epidehydroabietic acid) from dehydroabietic acid (2) by Pelletier and Herald.^7^ The necessary aldehydes for the key aldol reaction could be obtained after opening of the epoxide 8, which in turn is synthesized from a mixture of olefins 9 coming from an oxidative decarboxylation of DHA (2), obtainable from (−)-abietic acid (1).

**Scheme 2:**
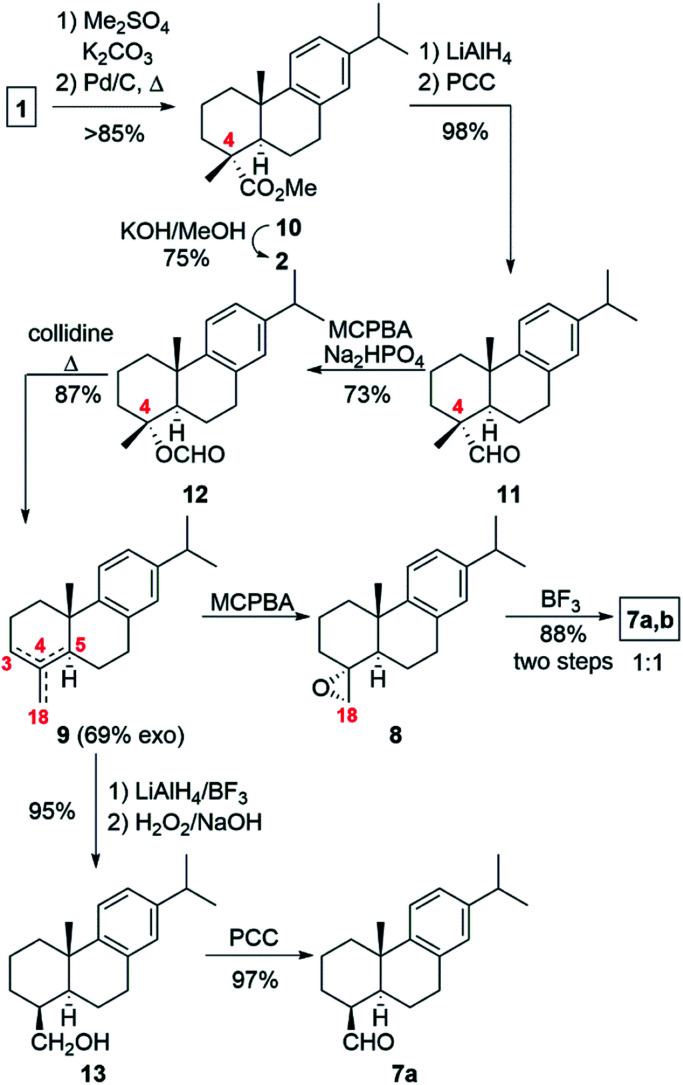
Synthesis of the key intermediate 7a.

With this straightforward synthetic plan in mind, we prepared DHA (2) from commercial (−)-abietic acid (1) following our method by methylation, aromatization with Pd/C catalyst and hydrolysis (Scheme 2).^1*c*,8^ We thought that the preparation of the exo-olefin 4(18), though as a mixture of regioisomers, would be easy and fast by treatment of DHA (2) with lead tetraacetate. But like other researchers found, the reaction was capricious and low yielding in the desired exo-olefin.^7,9^ We sought an alternative and decided to use the methodology of Barrero and co-workers based on the elimination of a formate moiety (Scheme 2).^10^ Thus, methyl dehydroabietate^1*c*^ (10) obtained from (−)-abietic acid (1) was converted into dehydroabietinal (11) in almost quantitative yield, which was oxidized with *m*-CPBA in the presence of disodium phosphate to furnish formate 12 in 73% yield. Heating in collidine at reflux of 12 led to a mixture of olefin regioisomers 9, *ca.* 5 : 1 : 1 (4,18-; 3,4-; 4,5-), in 87% yield where the major component contained the exocyclic 4,18-double bond. Continuing with our synthetic plan, epoxidation of that mixture of olefins 9 (*ca.* 69% purity in exo-olefin) with an excess of *m*-CPBA afforded epoxide 8, which was treated with BF_3_ etherate in toluene to give a 1 : 1 mixture of epimeric aldehydes 7a and 7b (88%, two steps). The introduction of the remaining carbon at C-4 and manipulation of the functionality at this site were the remaining steps to be accomplished. To our surprise, our first attempt of the planned aldol reaction^6^ (aq. HCHO/Na_2_CO_3_) with aldehydes 7a,b (*ca.* 1 : 1 mixture) did not work as expected since only β-aldehyde 7a reacted, recovering unaltered α-aldehyde 7b. At this point, we hypothesized that using a stronger base such as NaOH might help but little progress in the aldol reaction was observed when using recovered α-aldehyde 7b and NaOH as a base, even with slow addition of aq. HCHO.

Initially, it was attributed this high stereoselectivity to the presence of the axial C-20 methyl moiety, which probably hinders the approach of the base to the most hindered β-face.

This fact was investigated by computational methods and deuterium exchange experiments (see below) in order to determine the causes of the different behavior in the enolization step and hydroxymethylation of both aldehyde epimers at C-4, 7a and 7b.

In view of those results and other failed attempts to perform the aldol reaction with the epimer 7b, including organocatalysis, we modified slightly the planned synthetic sequence. Thus, the reactive aldehyde 7a (CHO-18β) was synthesized stereoselectively. To this end, 18-norabieta-8,11,13-trien-19-ol (13) was prepared by hydroboration/oxidation generating *in situ* BH_3_, treating BF_3_ etherate with LiAlH_4_, followed by addition of H_2_O_2_ in basic media.^11^ Oxidation of 13 with pyridinium chlorochromate (PCC) afforded the intermediate aldehyde 7a in high yield (92%, two steps).

**Scheme 3:**
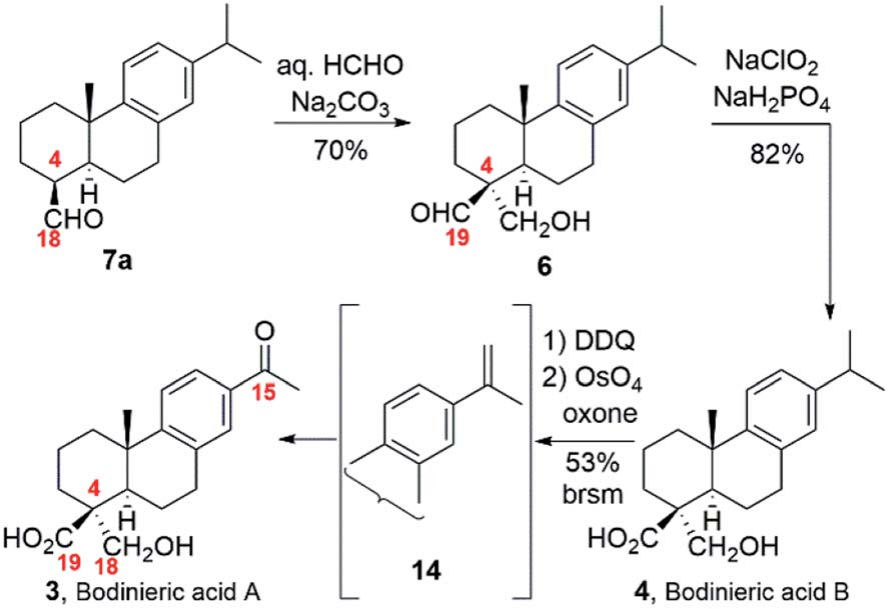
Synthesis of bodinieric acids A and B.

**Fig. 2:**
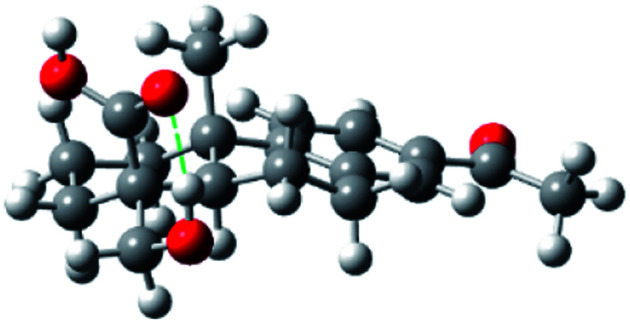
Geometry at B3LYP/6-31G** level of the most stable conformation of the bodinieric acid A (3), in acetone.

The completion of the syntheses of both bodinieric acids A and B is shown in Scheme 3. At this stage, the key aldol reaction of aldehyde 7a with aq. HCHO/Na_2_CO_3_ rapidly gave the desired α stereoselectivity as a single isomer, hydroxy-aldehyde 6 in high yield (70%). Selective oxidation of the aldehyde group of 6 with NaClO_2_ provided bodinieric acid B (4, callicapoic acid M4) in 82% yield, whose ^1^H and ^13^C NMR spectra (in CDCl_3_ and acetone-*d*_6_) and HRMS (calcd for C_20_H_29_O_3_ [M + H]^+^: 317.2117; found: 317.2111) were in complete agreement with the reported data for the natural product 4.^3,12^ Interestingly, Zhang and co-workers and Wang and co-workers defined wrongly this natural product as 18-hydroxydehydroabietic acid instead of 18-hydroxycallitrisic acid which contains the C-19 carboxylic acid, and 18-hydroxy-8,11,13-abietatetraen-19-oic acid instead of 18-hydroxy-8,11,13-abietatrien-19-oic acid, respectively.^3^ Our next synthetic target was bodinieric acid A (Scheme 3). Starting from bodinieric acid B (4), selective dehydrogenation at C-15 with dichloro dicyano quinone (DDQ) provided the isopropenyl moiety in 14 (bodinieric acid C, as a *ca.* 3 : 2 mixture with unreacted starting material). Subsequent oxidative cleavage of 14 with catalytic OsO_4_ and oxone as co-oxidant^13^ furnished bodinieric acid A (3) in moderate yield (53% brsm, two steps) which was not further optimized (10.2% overall yield from abietic acid, 13 steps, see Scheme S1‡). The data for synthetic 3 were in excellent agreement with the isolation data (^1^H NMR, ^13^C NMR, [*α*]_D_).^3*a*^ A conformational study of 3 was also carried out to estimate the ^13^C data with the GIAO (gauge including atomic orbital) method, implemented in the Gaussian package (see Fig. S1 and S2 and Tables, S1 and S2‡).^14^ Sixteen different conformations were obtained in acetone (solvent used in NMR experiments), the most stable being that indicated in Fig. 2. This conformation contains a hydrogen bond between the carbonyl of the acid group and the hydroxyl proton. A good correlation is observed between the experimental and theoretical ^13^C NMR data. In particular, our experimental values for 3 gave a mean deviation of 1.82 (Table S2‡). Only some sensitive carbons to the rotation of the carboxylic group such as C5 give a high deviation.

### Deuterium exchange experiments and computational study

To further get insight on the mechanism of the enolization, an experiment of deuterium exchange of hydrogens H-4 was designed and executed successfully. Thus, a *ca.* 1 : 1 mixture of aldehydes 7a/7b was subjected to equivalent conditions of the aldol reaction, without adding formaldehyde, with the corresponding solvents capable of releasing protons as deuterated versions (MeOD and D_2_O). The results (see ESI, Fig. S9–S12‡) indicate that after 60 minutes of reaction the proton H-4 of isomer 7a (β-aldehyde) was exchanged by deuterium, while that of isomer 7b (α-aldehyde) apparently remained unaltered. It is believed that with longer reaction times the isomer 7b should also react but in the real system with formaldehyde this reaction will be disfavored since formaldehyde will decompose in basic media.

**Scheme 4:**
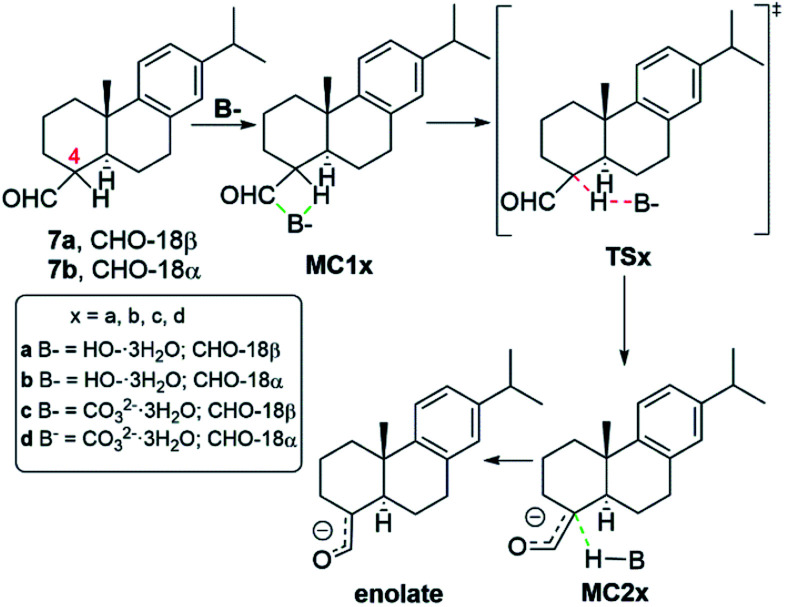
Mechanism of enolate formation starting from the alpha and beta aldehyde epimers 7.

The enolate formation of both aldehyde epimers 7 using as base HO^−^ and CO_3_^2−^ was studied by DFT methods. In Scheme 4, there is a representation of the mechanism of enolate generation, starting from 7a and 7b. Initially, both epimers form a molecular complex (MC1) with the base, which evolves through the corresponding transition state (TS) to another molecular complex (MC2). MC2 is composed by the enolate and the conjugated acid of the used base. We have considered that ion HO^−^ is solvated by three H_2_O molecules surrounding the oxygen with negative charge (see Fig. S7‡).^15^ In a similar way, the anion CO_3_^2−^ is also surrounded by three H_2_O molecules (see Fig. S8‡). All calculations have been made with those explicit H_2_O molecules. The energy results are presented in Table S4‡ (see also Fig. S3–S6‡) and the geometries of all the involved species are in Fig. S7 and S8.‡

**Fig. 3:**
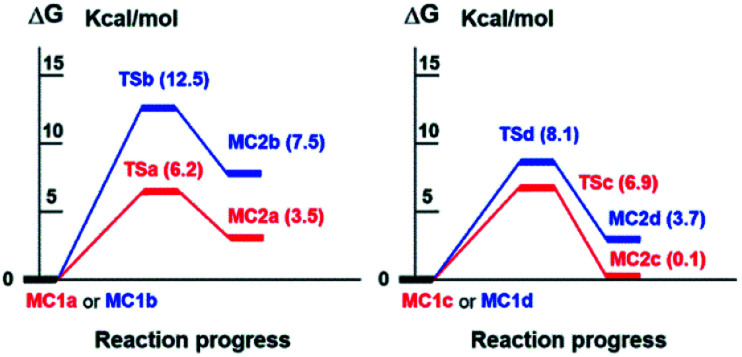
Free energy profile (Δ*G* in kcal mol^−1^) at B3LYP/6-31G** level of species involved in the enolate formation of 7a and 7b with HO^−^ (left) and CO_3_^2−^ (right), in water.

As it can be seen in Table S4‡ and in Fig. 3, the enolate formation of the epimer 7a (CHO-18β) with HO^−^ occurs through the TSa with a Gibbs free energy difference Δ*G* = 6.2 kcal mol^−1^ from MC1a. The enolate generation of the epimer 7b (CHO-18α) is clearly disfavored from the kinetic point of view (TSb, Δ*G* = 12.5 kcal mol^−1^ from MC1b). Nevertheless, both enolate formations are very endergonic (MC2a, Δ*G* = 3.5 kcal mol^−1^ and MC2b, Δ*G* = 7.5 kcal mol^−1^). As a result, a very low concentration of complex MC2 is obtained and therefore, low concentration of enolate.

It must be considered that formaldehyde under the conditions of reaction is an aqueous solution stabilized by methanol. Those conditions provide a low concentration of formaldehyde, since there are several equilibria on reacting formaldehyde with water to give methylene glycol (HOCH_2_OH) and poly(oxymethylene)glycols and with methanol to form hemiformal (CH_3_OCH_2_OH) and poly(oxymethylene)hemiformals.^16^ Thus, the low concentration of enolate and free formaldehyde makes the alkylation a slow reaction. This fact could be also accompanied by a side reaction, the Cannizzaro reaction,^17^ that normally occurs under basic conditions. As a final result formaldehyde decomposes without reacting with the enolate.

**Fig. 4:**
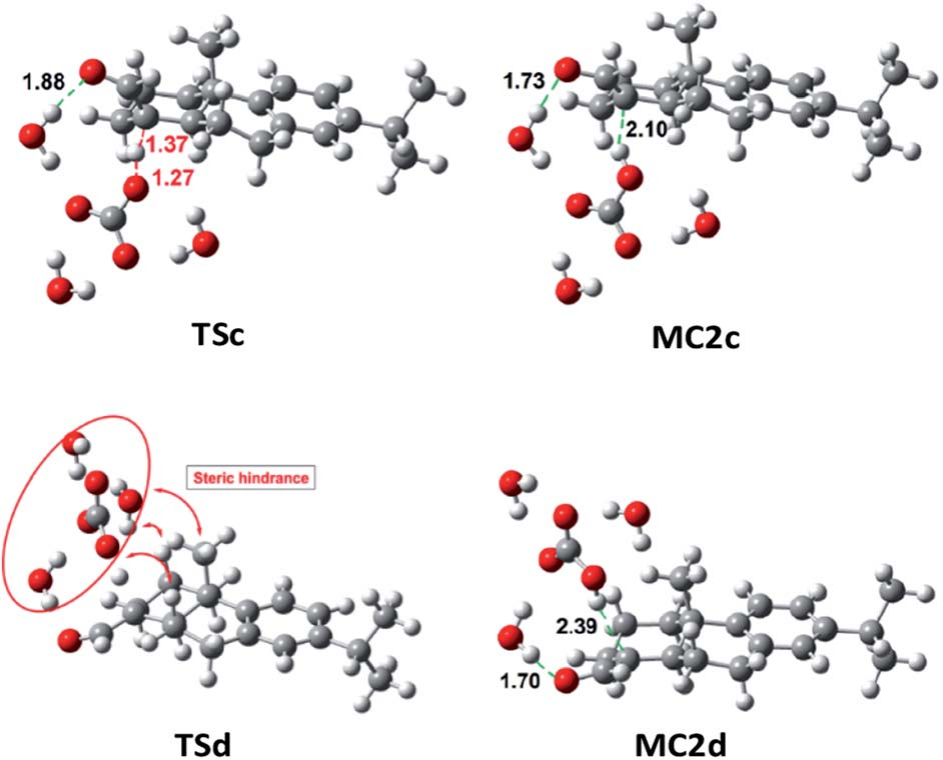
Geometries at B3LYP/6-31G** level of TSc, TSd, MC2c and MC2d involved in the enolate formation of 7a and 7b with CO_3_^2−^, in water.

On the other side, the use of CO_3_^2−^ as base changes the scenario notably. As it is shown in Table S4,‡Fig. 3 and 4, the reaction of 7b (CHO-18α) with CO_3_^2−^ leads to MC2d through TSd. The Gibbs free energy difference is 8.1 kcal mol^−1^ but the process is still very endergonic (MC2d, Δ*G* = 3.7 kcal mol^−1^) and therefore, disfavored. However, the enolate formation of 7a (CHO-18β) is fast (TSc, Δ*G* = 6.9 kcal mol^−1^), leading to MC2c with only 0.1 kcal mol^−1^ above the energy of MC1c. This allows a high concentration of the complex MC2c and therefore a high concentration of the corresponding enolate. In the soft basic media of CO_3_^2−^*vs.* OH^−^, the Cannizzaro reaction would be less important and, therefore, less decomposition of formaldehyde will occur. These two circumstances explain the experimental finding that only the epimer 7a reacts in a carbonate media (CO_3_^2−^). As it can be seen in Fig. 4, both the transition state and the molecular complex resulting from the enolate formation of aldehyde 7b with CO_3_^2−^ (TSd and MC2d, respectively), have a high steric hindrance with H-2β, H-6β and methyl 20. This is the main cause of the increase in energy of these species. This circumstance is even worse in the real situation since in the simulation only three water molecules have been considered and, in fact, there are more water molecules solvating the base.

The use of a more real model would have several consequences: (a) on considering a higher number of explicit water molecules would imply the stabilization of the reactant base and the MC1 complex due to H-bonding as compared to the transition state, which raises the barrier height and hinders reaction.^18^

(b) It is known that steric hindrance, *i.e.* S_N_2 reactions, increases the energy of the transition state.^19^ In our case, this effect would be similar especially for the steric characteristics of TSd, which would hinder the removal of the proton by the base.

(c) If we consider the effect of the solvated cation the steric effects would be even higher.^20^

As an overall result, it can be deduced that in a more real computational model the reactants will be stabilized in comparison with the TS. Therefore, including more explicit water molecules would probably lead to slower reactions,^18*a*^ which is in agreement with the deuterium exchange experiment.

## Conclusions

In summary, the first stereoselective synthesis of both C-18 and C-19 oxygenated abietane diterpenoids, in particular, bodinieric acids A and B, has been completed using an aldol reaction as key step enabling the synthesis of additional congeners of this new group of natural products. Those metabolites were isolated very recently and had not been previously prepared synthetically. Our synthesized materials established and confirmed the originally proposed absolute stereochemistry of each natural product since the structure of the starting material (−)-abietic acid is already known by single-crystal X-ray determination.^21^ The key aldol reaction was very sensitive to steric hindrance. This fact has been studied by deuterium exchange experiments and DFT methods. Further application of the intermediates obtained and biological evaluation of these synthetic natural products and related analogues are underway and will be reported in due course.

## Experimental section

### General experimental procedures

The melting points were measured with a Büchi M-560 apparatus. Optical rotations were measured using a 10 cm cell in a Jasco P-2000 polarimeter in DCM unless otherwise stated. NMR spectra were recorded on a 300 MHz spectrometer (^1^H: 300 MHz, ^13^C: 75 MHz) and referenced to the solvent peak at 7.26 ppm (^1^H) and 77.00 ppm (^13^C) for CDCl_3_ and 2.05 ppm (^1^H) and 29.84 ppm (^13^C) for acetone-*d*_6_. All spectra were recorded in CDCl_3_ as solvent unless otherwise stated. Complete assignments of ^13^C NMR multiplicities were made on the basis of DEPT experiments and further assignment of signals with the help of COSY and HSQC experiments. *J* values are given in Hz. MS data were acquired on a QTOF spectrometer. Reactions were monitored by TLC using Merck silica gel 60 F_254_ (0.25 mm-thick) plates. Compounds on TLC plates were detected under UV light at 254 nm and visualized by immersion in a 10% sulfuric acid solution and heating with a heat gun. Purifications were performed by flash chromatography on Merck silica gel (230–400 mesh). Commercial reagent grade solvents and chemicals were used as purchased unless otherwise noted. Combined organic extracts were washed with brine, dried over anhydrous MgSO_4_, filtered, and concentrated under reduced pressure. The starting material, methyl dehydroabietate (10), was obtained following our reported protocol^8^ with the following modifications: abietic acid (1, >70%, TCI) was methylated under standard conditions with dimethyl sulfate (1.2 equiv.) and K_2_CO_3_ (1.3 equiv.) in acetone (0.33 M in abietic acid, 30 g scale). The resulting methyl abietate was aromatized with 10% Pd/C catalyst (1% of ester mass), heating at 240 °C for 3 h. Next, compound 10 was reduced with LiAlH_4_ (5 equiv.) in THF (0.23 M in methyl dehydroabietate, 8 g scale) and subsequently the crude product was oxidized with PCC (1.5 equiv.) in DCM (0.24 M in dehydroabietinol, 5 g scale) to give dehydroabietinal 11 (quantitative) which was used in the next step without further purification. The carbon numbering of all synthetic compounds corresponds to that of natural products.

### Computational methods

All calculations were carried out with the Gaussian 09 suite of programs.^14^ Initially, density functional theory^22^ calculations (DFT) have carried out using the B3LYP^23^ exchange-correlation functionals, together with the standard 6-31G** basis set, in gas phase.^24^ Subsequently, the inclusion of solvent effects have been considered by using a relatively simple self-consistent reaction field (SCRF) method^25^ based on the polarizable continuum model (PCM).^26^ Geometries have been fully optimized with PCM. As solvents we have used H_2_O. In the calculations, three water molecules have been explicitly included. The values of enthalpies, entropies and free energies in H_2_O were calculated with the standard statistical thermodynamics at 298.15 K.^24^ The stationary points were characterized by frequency computations in order to verify that TSs have one and only one imaginary frequency. The intrinsic reaction coordinate (IRC) paths^27^ were traced in order to check the energy profiles connecting each TS to the two associated minima of the proposed mechanisms using the second order González–Schlegel integration method.^28^

#### 18-Norabieta-8,11,13-trien-4-yl formate (12)

A suspension of *m*-CPBA (75%, 10.0 g, 0.043 mol, 2.5 equiv.) and Na_2_HPO_4_ (6.7 g, 0.047 mol, 2.7 equiv.) in DCM (60 mL) was added to a solution of crude aldehyde 11 (5.0 g, 0.017 mol) in DCM (120 mL) and heated at reflux for 4 h. Then, the mixture was cooled to rt and diluted with Et_2_O (150 mL), washed with saturated aqueous NaHCO_3_ (3 × 80 mL), brine (2 × 50 mL), dried, and concentrated. The resulting oily yellow residue was chromatographed on silica eluting with *n*-hexane–EtOAc (9 : 1) to give 3.7 g (73%) of formate 12 as a yellowish oil: [*α*]^25^_D_ +9.5 (c 1.0, DCM) (lit.,^29^ +27.9 (c 1.0, CHCl_3_); ^1^H NMR (300 MHz) *δ* 8.08 (1H, s, OC*H*O), 7.17 (1H, d, *J* = 8.1), 7.01 (1H, dd, *J* = 8.1, 2.1), 6.91 (1H, s), 2.93 (2H, m), 2.84 (1H, m), 2.64 (1H, m), 2.25 (1H, m), 1.59 (3H, s), 1.23 (3H, d, *J* = 6.9), 1.23 (3H, d, *J* = 6.9)), 1.20 (3H, s), 0.89 (2H, m); ^13^C NMR (75 MHz) *δ*_C_ 160.5 (d), 145.9 (s), 134.6 (s), 127.0 (d), 124.5 (d), 124.1 (d), 87.1 (s), 49.7 (d), 38.4 (s), 38.0 (t), 37.5 (t), 33.5 (d), 30.1 (t), 24.8 (q), 24.0 (q), 24.0 (q), 20.0 (t), 19.9 (q), 18.4 (t); HRMS (ESI) *m*/*z* 323.1975 [M + Na]^+^, calcd for C_20_H_28_O_2_Na: 323.1987.

#### 19-Norabieta-4(18),8,11,13-tetraene (9)

A yellowish solution of formate 12 (3.7 g, 0.012 mmol) in 2,4,6-collidine (16 mL) was heated at reflux for 7 h. During this time, the solution became dark orange then cooled to rt and diluted with Et_2_O (110 mL). The mixture was washed with 10% HCl (3 × 30 mL) and brine (3 × 30 mL), dried and concentrated. The resulting dark orange-red oil residue was dissolved in *n*-hexane and few drops of DCM. This solution was chromatographed on a short pad of silica eluting with *n*-hexane to give 2.75 g of olefins 9 (87%, exocyclic Δ^4(18)^*ca.* 69% purity estimated by ^1^H NMR), containing the three corresponding regioisomers in an inseparable mixture by standard flash chromatography *ca.* 5 : 1 : 1 (4,18-; 3,4-; 4,5-). The ^1^H and ^13^C NMR data for the major exocyclic isomer are:^9*a*^^1^H NMR (300 MHz) *δ* 7.22 (1H, d, *J* = 8.1), 7.01 (1H, dd, *J* = 8.1, 2.2), 6.93 (1H, s), 4.85 (1H, d, *J* = 1.8), 4.60 (1H, d, *J* = 1.8), 2.90–2.77 (3H, m), 1.24 (6H, d, *J* = 7.2), 1.00 (3H, s); ^13^C NMR (75 MHz) *δ*_C_ 150.7 (s), 145.7 (s), 144.6 (s), 134.8 (s), 127.1 (d), 125.3 (d), 123.9 (d), 106.3 (t), 47.9 (d), 39.2 (s), 38.4 (t), 36.4 (t), 33.5 (d), 30.0 (t), 24.0 (q), 24.0 (q), 23.8 (t), 22.8 (q), 21.4 (t).

#### 4α,19-Epoxide-18-norabieta-8,11,13-triene (8)

A solution of the olefins 9 (760 mg, 3.0 mmol) in DCM (70 mL) was treated with an excess of *m*-CPBA (75%, 1.2 g, 5.2 mmol, 1.7 equiv.) and stirred at rt for 3 h 30 min. The reaction mixture was quenched with 5% aqueous Na_2_S_2_O_3_ (20 mL) and washed with a solution at 50% made with saturated aqueous NaHCO_3_ (3 × 20 mL), brine, dried and concentrated. The resulting crude epoxide (810 mg) was isolated as a yellowish oil and was used in the next step without further purification (it contained about 10% of 19-norabieta-4(18),8,11,13-tetraen-3α-ol).^30^^1^H NMR (300 MHz) *δ* 7.21 (1H, d, *J* = 8.1), 7.01 (1H, dd, *J* = 8.1, 2.1), 6.92 (1H, s), 2.90–2.79 (4H, m), 2.65 (1H, d, *J* = 4.6), 2.28 (1H, m), 1.23 (6H, d, *J* = 6), 1.14 (3H, s).

#### Rearrangement of 4α,19-epoxide-18-norabieta-8,11,13-triene (8) (mixture aldehydes 7a,b)

Boron trifluoride etherate (400 μL, *ca.* 2 equiv.) was added at 15 °C to a solution of the previous crude 4α-19-epoxide 8 (400 mg, 1.48 mmol) in dry toluene (10 mL) under Ar atmosphere. The reaction mixture was stirred for 3 min and saturated aqueous NaHCO_3_ (1 mL) was added. Then, the mixture was diluted with Et_2_O (15 mL), washed with brine, dried and concentrated to give a dark pale oil (410 mg). The oily residue was carefully chromatographed on silica eluting with *n*-hexane–EtOAc (9 : 1) to give 245 mg (88% based on the Δ^4(18)^-isomer in the alkenes 9 mixture) of epimeric aldehydes 7a,b (NMR data *vide infra*) as a colorless oil which were used in the next step directly (aldol reaction).

#### 18-Norabieta-8,11,13-trien-19-ol (13)

To an ice-cold solution of alkenes 9 mixture (*ca.* 69%, 2.75 g, 10.8 mmol) in dry Et_2_O (60 mL) was added LiAlH_4_ (1.03 g, 27.0 mmol, 2.5 equiv.) and a solution of boron trifluoride etherate (3.4 mL, 27.0 mmol, *ca.* 2.5 equiv.) in dry Et_2_O (48 mL) during 20 min. Then, the mixture was stirred for 4 h at rt, cooled at 0 °C and treated with 5 mL of saturated Na_2_SO_4_ and some anhydrous solid Na_2_SO_4_. The solids were filtered off and the diethyl ether layer was replaced by THF (90 mL) after concentrating to leave a viscous colorless oil. The resulting solution was treated with 10% aqueous NaOH (40 mL) and 30% H_2_O_2_ (30 mL) and the biphasic mixture was stirred vigorously for 15 h. After this time, the reaction mixture was diluted with H_2_O (50 mL) and extracted with Et_2_O (3 × 30 mL). The combined organic extracts were washed with brine, dried and concentrated. The resulting oily residue was chromatographed on silica eluting with *n*-hexane–EtOAc (6 : 4) to give 2.0 g (>95%) of 13 as a colorless oil: [*α*]^25^_D_ +110.9 (c 1.0, DCM). The ^1^H NMR data were in agreement with those reported in the literature:^30^^1^H NMR (300 MHz) *δ* 7.18 (1H, d, *J* = 8.1), 7.01 (1H, dd, *J* = 8.1, 2.1), 6.90 (1H, d, *J* = 2.1), 3.75 (2H, m), 2.93–2.84 (3H, m), 2.27 (1H, br d, *J* = 11.5), 1.24 (6H, d, *J* = 6.9), 1.06 (3H, s); ^13^C NMR (75 MHz) *δ*_C_ 146.1 (s), 145.6 (s), 134.7 (s), 127.0 (d), 124.5 (d), 123.9 (d), 61.7 (t), 44.5 (d), 44.0 (d), 38.6 (t), 36.9 (s), 33.4 (d), 30.6 (t), 27.6 (t), 25.3 (t), 24.3 (q), 24.0 (q), 24.0 (q), 18.2 (t); HRMS (ESI) *m*/*z* 336.2343 [M + CH_3_CN + Na]^+^, calcd for C_21_H_31_NONa: 336.2303.

#### 18-Norabieta-8,11,13-trien-19-al (7a)

A solution of the alcohol 13 (2.0 g, 7.3 mmol) was dissolved in DCM (160 mL) and treated with PCC (3.0 g, 13.9 mmol, 1.9 equiv.). After stirring for 2 h, the mixture was filtered through a short pad of silica eluting with DCM to afford the corresponding β-aldehyde 7a (1.93 g, 97%) as a yellowish oil which was used in the next step without further purification: [*α*]^24^_D_ +178.0 (c 1.0, DCM). The ^1^H NMR data were in agreement with those reported in the literature:^7^^1^H NMR (300 MHz) *δ* 10.03 (1H, d, *J* = 1.2), 7.18 (1H, d, *J* = 8.1), 7.02 (1H, dd, *J* = 8.1, 2.1), 6.93 (1H, d, *J* = 2.1), 3.00–2.94 (2H, m), 2.84 (1H, sept., *J* = 6.9), 1.24 (3H, d, *J* = 6.9), 1.23 (3H, d, *J* = 6.9), 1.04 (3H, s); ^13^C NMR (75 MHz) *δ*_C_ 204.7 (d), 146.0 (s), 144.6 (s), 134.4 (s), 127.1 (d), 124.8 (d), 124.1 (d), 52.3 (d), 45.0 (d), 38.1 (t), 37.5 (s), 33.5 (d), 30.8 (t), 24.8 (t), 24.2 (t), 24.0 (q), 24.0 (q), 23.7 (q), 19.1 (t); HRMS (ESI) *m*/*z* 293.1921 [M + Na]^+^, calcd for C_19_H_26_ONa: 293.1881.

#### 18-Hydroxyabieta-8,11,13-trien-19-al (6)

A solution of the aldehyde 7a (1.91 g, 7.0 mmol) was dissolved in a 1 : 1 MeOH–DCM mixture (120 mL) and treated with excess 37% aqueous HCHO (25 mL, 0.336 mol, 48 equiv.) and Na_2_CO_3_ (400 mg, 3.8 mmol, 0.54 equiv.) under an Ar atmosphere. After being stirred for 5 h, the mixture was diluted with DCM (120 mL) and washed with a mixture of 30 mL of water and 30 mL of brine, dried and concentrated. The crude oily residue was chromatographed on silica eluting with *n*-hexane–EtOAc (7 : 3) to give 1.5 g (70%) of aldol 6 as a colorless solid which solidified (white solid) upon standing at 5 °C: mp 113–115 °C; [*α*]^24^_D_ +76.3 (c 1.0, DCM). ^1^H NMR (300 MHz) *δ* 9.97 (1H, d, *J* = 1.2), 7.18 (1H, d, *J* = 8.4), 7.02 (1H, dd, *J* = 8.4, 2.1), 6.91 (1H, d, *J* = 2.1), 3.96 (1H, d, *J* = 10.8), 3.56 (1H, d, *J* = 10.8), 3.00–2.90 (2H, m), 2.83 (1H, sept., *J* = 6.9), 2.34 (2H, m), 2.40–1.60 (6H, m), 1.42 (1H, ddd, *J* = 12.6, 12.1, 5.1), 1.23 (6H, d, *J* = 6.9), 1.09 (3H, s); ^13^C NMR (75 MHz) *δ*_C_ 205.9 (d), 146.1 (s), 144.8 (s), 134.2 (s), 126.9 (d), 124.8 (d), 124.1 (d), 67.2 (t), 54.0 (s), 47.2 (d), 38.0 (t), 37.5 (s), 33.5 (d), 30.7 (t), 29.0 (t), 24.7 (q), 23.9 (q), 23.9 (q), 19.1 (t), 18.6 (t); HRMS (ESI) *m*/*z* 301.2170 [M + Na]^+^, calcd for C_20_H_29_O_2_: 301.2168.

When this procedure was first carried out with the *ca.* 1 : 1 mixture of aldehydes 7a,b, as starting material, we recovered unreacted aldehyde 7b (19-norabieta-8,11,13-trien-18-al, *R*_f_ = 0.50 in *n*-hexane–EtOAc (7 : 3)), which eluted prior to the aldol product 6, as a semisolid: ^1^H NMR (300 MHz) *δ* 9.54 (1H, d, *J* = 4.5), 7.21 (1H, d, *J* = 8.1), 7.01 (1H, br d, *J* = 8.4), 6.91 (1H, d, *J* = 2.1), 2.90–2.75 (3H, m), 1.23 (6H, d, *J* = 6.9), 1.12 (3H, s); ^13^C NMR (75 MHz) *δ*_C_ 205.2 (d), 146.1 (s), 144.4 (s), 134.6 (s), 127.1 (d), 124.4 (d), 123.9 (d), 51.2 (d), 41.6 (d), 37.1 (t), 36.0 (s), 33.5 (d), 29.0 (t), 26.4 (t), 24.0 (q), 24.0 (q), 23.0 (t), 22.7 (q), 20.6 (t).

#### 18-Hydroxyabieta-8,11,13-trien-19-oic acid (4, bodinieric acid B or callicapoic acid M4)

To a stirred solution of aldol 6 (500 mg, 1.67 mmol) and 2-methyl-2-butene (5 mL, 47.2 mmol) in THF (5 mL) and *tert*-BuOH (16 mL) at rt was added NaClO_2_ (80%, 1.20 g, 10.6 mmol, 6 equiv.) and NaH_2_PO_4_ (1.20 g, 10.0 mmol, 6 equiv.) in H_2_O (7 mL). The reaction mixture was capped with a rubber septa with a needle as outlet and stirred for 4 h. Then, the solvent was removed under vacuum (bath temperature 40 °C) and the resulting watery residue was diluted with EtOAc (80 mL) and 10% HCl (40 mL) and brine (20 mL). The phases were separated and the aqueous layer was extracted with EtOAc (3 × 20 mL) and the combined organic extracts were washed with brine (20 mL), dried and concentrated. The resulting pale oil was chromatographed on silica eluting with DCM–MeOH (9 : 1) to give 432 mg (82%) of acid 4 as an amorphous white solid: [*α*]^22^_D_ +104.9 (c 0.10, MeOH) (lit.,^3*a*^ +57.3 (c 0.10, MeOH)) (lit.,^3*b*^ +22.2 (c 0.10, MeOH)). The ^1^H and ^13^C NMR data were in agreement with those of the natural product in both acetone-*d*_6_ (ref. 3*a*) and CDCl_3_ (ref. 3*b*): ^1^H NMR (300 MHz, CD_3_COCD_3_) *δ* 7.21 (1H, d, *J* = 8.1), 6.97 (1H, dd, *J* = 8.1, 2.1), 6.86 (1H, d, *J* = 2.1), 3.87 (1H, d, *J* = 10.2), 3.66 (1H, d, *J* = 10.2), 2.90–2.70 (3H, m), 2.31 (2H, m), 1.78 (1H, m), 1.65 (1H, m), 1.19 (6H, d, *J* = 6.9), 1.15 (3H, s); ^13^C NMR (75 MHz, CD_3_COCD_3_) *δ*_C_ 177.4 (s), 146.7 (s), 146.3 (s), 135.7 (s), 127.5 (d), 126.2 (d), 124.6 (d), 70.2 (t), 50.6 (s), 47.5 (d), 40.1 (t), 38.8 (s), 34.3 (d), 32.5 (t), 32.3 (t), 24.4 (q), 24.3 (q), 24.0 (q), 21.7 (t), 20.4 (t); ^1^H NMR (300 MHz, CDCl_3_) *δ* 7.18 (1H, d, *J* = 8.1), 6.99 (1H, dd, *J* = 8.4, 2.1), 6.89 (1H, br s), 4.17 (1H, d, *J* = 10.2), 3.50 (1H, d, *J* = 10.8), 2.90–2.70 (3H, m), 2.45 (1H, br d, *J* = 12.9), 2.29 (1H, br d, *J* = 12.9), 2.10–2.00 (3H, m), 1.72 (2H, m), 1.22 (6H, d, *J* = 6.9), 1.15 (3H, s); ^13^C NMR (75 MHz, CDCl_3_) *δ*_C_ 181.3 (s), 145.8 (s), 145.3 (s), 134.6 (s), 126.8 (d), 125.3 (d), 124.0 (d), 71.4 (t), 49.9 (s), 47.7 (d), 38.9 (t), 38.1 (s), 33.4 (d), 31.9 (t), 31.5 (t), 23.9 (q), 23.9 (q), 23.4 (q), 20.8 (t), 19.3 (t); HRMS (ESI) *m*/*z* 317.2111 [M + H]^+^, calcd for C_20_H_29_O_3_: 317.2117.

#### 17-Nor-18-hydroxy-15-oxoabieta-8,11,13-triene-19-oic acid (3, bodinieric acid A)

A solution of hydroxyacid 4 (75 mg, 0.237 mmol) and dichloro dicyano quinone (62 mg, 0.273, 1.15 equiv.) in benzene was refluxed for 2 h under an Ar atmosphere. The, the mixture was cooled and filtered through a short pad of Celite 512 washing with 3.5 mL of fresh benzene and concentrated to give the crude alkene-acid 14 (70 mg, *ca.* 3 : 2 mixture with starting material based on ^1^H NMR integration) as a pale-brown semisolid which was used in the next step without further purification (same *R*_f_). The ^1^H and ^13^C NMR data (from the mixture) were in agreement with those of the natural product (14, 18-hydroxy-8,11,13,15-abietatetraen-19-oic acid, callicapoic acid M3) in CDCl_3_,^3*b*^ with the exception for signal reported at 8.25 ppm that we believe contains a typrographical error and it should be 7.25 ppm: ^1^H NMR (300 MHz, CDCl_3_) *δ* 7.25 (1H, d, *J* = 8.1), 7.18 (1H, d, *J* = 8.1), 7.13 (1H, br s), 5.32 (1H, s), 5.02 (1H, s), 4.13 (1H, d, *J* = 10.5), 3.55 (1H, d, *J* = 10.5), 2.90–2.70 (3H, m), 2.43 (1H, br d, *J* = 12.3), 2.30 (1H, m), 2.11 (3H, s), 1.73 (2H, m), 1.16 (3H, s); ^13^C NMR (75 MHz, CDCl_3_) *δ*_C_ 181.0 (s), 147.2 (s), 142.9 (s), 138.4 (s), 134.7 (s), 126.0 (d), 125.3 (d), 123.1 (d), 111.7 (t), 71.2 (t), 49.9 (s), 47.5 (d), 38.8 (t), 38.2 (s), 31.8 (t), 31.5 (t), 23.3 (q), 21.7 (q), 20.8 (t), 19.3 (t).

The crude alkene-acid 14 (70 mg, 0.142 mmol, *ca.* 3 : 2 mixture) obtained above was dissolved in DMF (720 μL, 0.2 M) and OsO_4_ (20 μL, 2.5% in *tert*-BuOH, 0.01 equiv.) was added and stirred for 5 min. Oxone (178 mg, 0.58 mmol, 4 equiv.) was added (the solution darkened) in one portion and the reaction was stirred at rt for 3 h 30 min. Then, 10% aqueous Na_2_SO_3_ (1 mL) and Et_2_O (7 mL) were added and stirred for 1 h. After separation of layers, the aqueous phase was extracted with 2 mL of Et_2_O and the combined organic extracts were washed with 1 N HCl, brine, dried and concentrated. The resulting pale oily residue was chromatographed on silica eluting with *n*-hexane–acetone (5 : 5) to give 13 mg of unreacted impurity (isopropyl moiety) contained in the starting alkene-acid (isopropenyl moiety), followed by 17.0 mg (38% for the two steps, 53% brsm) of bodinieric acid A (3) as an amorphous white solid: [*α*]^22^_D_ +125 (c 0.5, MeOH) (lit.,^3*a*^ +121 (c 0.52, MeOH)). The ^1^H and ^13^C NMR data were in agreement with those of the natural product in acetone-*d*_6_:^3*a*^^1^H NMR (300 MHz, CD_3_COCD_3_) *δ* 7.71 (1H, dd, *J* = 8.1, 2.1), 7.66 (1H, d, *J* = 2.1), 7.45 (1H, d, *J* = 8.4), 3.85 (1H, d, *J* = 10.2), 3.69 (1H, d, *J* = 10.2), 2.52 (3H, s), 2.35 (2H, m), 1.87 (2H, m), 1.66 (1H, m), 1.19 (3H, s); ^13^C NMR (75 MHz, CD_3_COCD_3_) *δ*_C_ 197.7 (s), 177.3 (s), 154.6 (s), 136.5 (s), 135.6 (s), 130.0 (d), 126.7 (d), 126.3 (d), 69.9 (t), 50.6 (s), 46.9 (d), 39.7 (t), 39.5 (s), 32.3 (t), 32.1 (t), 26.6 (q), 23.7 (q), 21.4 (t), 20.3 (t); HRMS (ESI) *m*/*z* 315.1599 [M − H]^+^, calcd for C_19_H_23_O_4_: 315.1596.

### Deuterium exchange experiment of a 1 : 1 mixture of aldehydes 7a,b with base in deuterated solvent

A 1 : 1 mixture of aldehydes 7a/7b (64 mg, 0.237 mmol) was dissolved in 1 : 1 DCM : MeOD (5 mL) and 1 mL of D_2_O was added followed by Na_2_CO_3_ (15 mg). After being stirred for 3 minutes an aliquot (2 mL) was taken from the reaction mixture and diluted with 4 mL of DCM in a separation funnel. Then, 2 mL of D_2_O with a bit of sodium chloride was added, agitated and the resulting phases were separated. The organic layer was dried on MgSO_4_ and concentrated. Another aliquot (2 mL) was taken after 15 minutes of reaction time and processed similarly. The remaining 2 mL of reaction mixture after 60 minutes of reaction time were processed accordingly. Thus, we obtained three residues of 20, 21, and 21 mg, respectively, as colorless oils which were then studied spectroscopically by NMR (see Fig. S9–S11‡).

## Conflicts of interest

There are no conflicts to declare.

## Supplementary Material

RA-010-D0RA02711A-s001

## References

[cit1] González M. A. (2015). Nat. Prod. Rep..

[cit2] González M. A. (2015). Tetrahedron.

[cit3] Gao J.-B., Yang S.-J., Yan Z.-R., Zhang X.-J., Pu D.-B., Wang L.-X., Li X.-L., Zhang R.-H., Xiao W.-L. (2018). J. Nat. Prod..

[cit4] Xiao W. , Zhang R., Gao J., Yang S., Zhang X., Pu D. and Li X., patent number CN 108129295, application number CN 2018-10028568, 2018

[cit5] Arnó M., González M. A., Marín M. L., Zaragozá R. J. (2000). J. Org. Chem..

[cit6] Ziegler F. E., Wallace O. B. (1995). J. Org. Chem..

[cit7] Pelletier S. W., Herald D. L. (1971). J. Chem. Soc. D.

[cit8] González M. A., Perez-Guaita D., Correa-Royero J., Zapata B., Agudelo L., Mesa-Arango A., Betancur-Galvis L. (2010). Eur. J. Med. Chem..

[cit9] Huffman J. W. (1970). J. Org. Chem..

[cit10] Barrero A. F., Alvarez-Manzaneda E. J., Alvarez-Manzaneda R., Chahboun R., Meneses R., Aparicio M. (1999). Synlett.

[cit11] Burghstahler A. W., Marx J. N. (1969). J. Org. Chem..

[cit12] In the original paper of Wang and co-workers (ref. 3*b*) for natural callicapoic acid M4 (4), the observed optical rotation ([*α*]_D_ = +22.2, *c* = 0.10, MeOH) is different from the reported value ([*α*]_D_ = +57.3, *c* = 0.10, MeOH) for bodinieric acid B (4) by Zhang and co-workers (ref. 3*a*), and also different of the value for our synthetic 4 ([*α*]_D_= +104.9, *c* = 0.10, MeOH). Our synthesized material 4 was used for the synthesis of bodinieric acid A (3) and we obtained a value for our synthetic 3 ([*α*]_D_= +125.0, *c* = 0.5, MeOH) which is in excellent agreement with the optical rotation ([*α*]_D_ = +121.0, *c* = 0.52, MeOH) reported by Zhang and co-workers (ref. 3*a*)

[cit13] Travis B. R., Narayan R. S., Borhan B. (2002). J. Am. Chem. Soc..

[cit14] Frisch M. J. , et al., Gaussian 09, Revision A.02, Gaussian, Inc., Wallingford, CT, 2009

[cit15] Hermida-Ramón J. M., Karlström G. (2003). J. Phys. Chem. A.

[cit16] Winkelman J. G. M., Voorwinde O. K., Ottens M., Beenackers A. A. C. M., Janssen L. P. B. M. (2002). Chem. Eng. Sci..

[cit17] Smith M. B. , and March J., March's Advanced Organic Chemistry: Reactions, Mechanisms, and Structure, 6th edn, Wiley, New Jersey, 2007, pp. 1863–1865

[cit18] Bohme D. K., Mackay G. I. (1981). J. Am. Chem. Soc..

[cit19] Liu X., Zhang J., Yang L., Hase W. L. (2018). J. Am. Chem. Soc..

[cit20] Raiteri P., Demichelis R., Gale J. D. (2015). J. Phys. Chem. C.

[cit21] González M. A., Gil-Gimeno M. J., Blake A. J. (2006). Acta Crystallogr., Sect. E: Struct. Rep. Online.

[cit22] (a) Parr R. G. and Yang W., Density Functional Theory of Atoms and Molecules, Oxford University Press, New York, 1989

[cit23] Becke A. D. (1993). J. Chem. Phys..

[cit24] Hehre W. J. , Radom L., von R. Schleyer P. and Pople J. A., Ab initio Molecular Orbital Theory, Wiley, New York, 1986

[cit25] Fukui K. (1970). J. Phys. Chem..

[cit26] González C., Schlegel H. B. (1990). J. Phys. Chem..

[cit27] (b) Simkin B. Y. , and Sheikhet I., Quantum Chemical and Statistical Theory of Solutions-A Computational Approach, Ellis Horwood, London, 1995

[cit28] Cances E., Mennunci B., Tomasi J. A. (1997). J. Chem. Phys..

[cit29] Caputo R., Previtera L., Monaco P., Mangoni L. (1974). Tetrahedron.

[cit30] Tagat J. R., Nazareno D. V., Puar M. S., McCombie S. W., Ganguly A. K. (1994). Bioorg. Med. Chem. Lett..

