# Supplementary material for: Synthesis of bodinieric acids A and B, both C-18 and C-19-functionalized abietane diterpenoids: DFT study of the key aldol reaction
Source: RSC Adv. 2020 Apr 16;10(25):15015–22. doi: 10.1039/d0ra02711a (PMC9052302; doi:10.1039/d0ra02711a)
Supplement: RA-010-D0RA02711A-s001 [file RA-010-D0RA02711A-s001.pdf]

Electronic Supplementary Material (ESI) for RSC Advances.
This journal is © The Royal Society of Chemistry 2020

Supporting Information

Synthesis of bodinieric acids A and B, both C-18 and C-19-funtionalized abietane diterpenoids: DFT study of the key aldol reaction.

Ramón J. Zaragozá[a] and Miguel A. González-Cardenete*[b]*

[a] Departamento de Química Orgánica, Universidad de Valencia, Dr. Moliner 50, 46100 Burjassot, Valencia, Spain

[b] Instituto de Tecnología Química (UPV-CSIC), Universitat Politècnica de Valencia-Consejo Superior de Investigaciones Científicas, Avda de los Naranjos s/n, 46022 Valencia, Spain

migoncar@itq.upv.es

TABLE OF CONTENTS

**Scheme S1.** Synthesis of Bodinieric acids A and B from abietic acid	S2
Copies of ^{1}H, ^{13}C, DEPT135 NMR spectra	S3–34
Copies of COSY and HSQC for **3** and **4**	S35-42
Conformational study and ^{13}C estimation data for **3**	S43-46
**Figure S1**. Different conformations that the groups present in bodinieric acid A (**3**) can display A/B -CO_2H group, a/b/c –CH_2OH group and x/y –$COCH_3$ group.	S43
**Figure S2**. Geometries at B3LYP/6-31G** level of the conformations of the bodinieric acid A (**3**), in acetone (solvent used in NMR).	S44
**Table S1**. B3LYP/6-31G** total energies (*E*, au) and Δ*E* (Kcal/mol) of the conformations of the bodinieric acid A (**3**), in acetone	S45
**Table S2**. Comparison of ^{13}C data for natural and synthetic **3** and calculated values	S46
**Table S3**. Comparison of ^{13}C data for natural and synthetic **4**	S47
**Table S4**. B3LYP/6-31G** total energies (*E*, *H* and *G*, au), *S* (Cal/mol-kelvin, 298.15 K) and relative energies (Δ*G*, Kcal/mol) of species involved in the enolization of **7a** and **7b** with HO^- and CO_3^{2-}, in water.	S48
**Figures S3** to **S6**. IRCs	S49
**Figure S7**. Geometries at B3LYP/6-31G** level of species involved in the enolization of **7a** and **7b** with HO^-, in water.	S50
**Figure S8**. Geometries at B3LYP/6-31G** level of species involved in the enolization of **7a** and **7b** with CO_3^{2-}, in water.	S51
XYZ matrices B3LYP/6-31G**	S52-67
Exchange experiment of a 1:1 mixture of aldehydes **7** with base in deuterated solvent	S68
**Figures S9-S11**. ^{1}H and ^{13}C spectra zoom in the exchange experiment after 0’, 5‘, 15’ and 60’ of reaction	S69-71

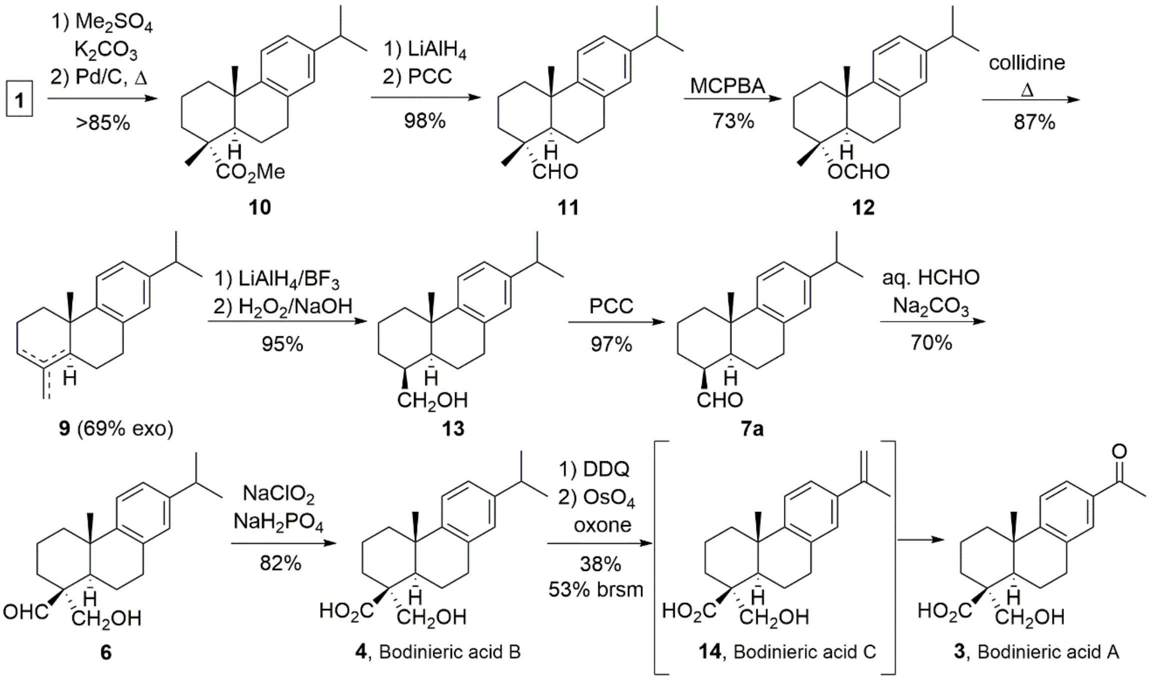

Scheme S1. Synthesis of bodinieric acids A and B from abietic acid.

Compound 12

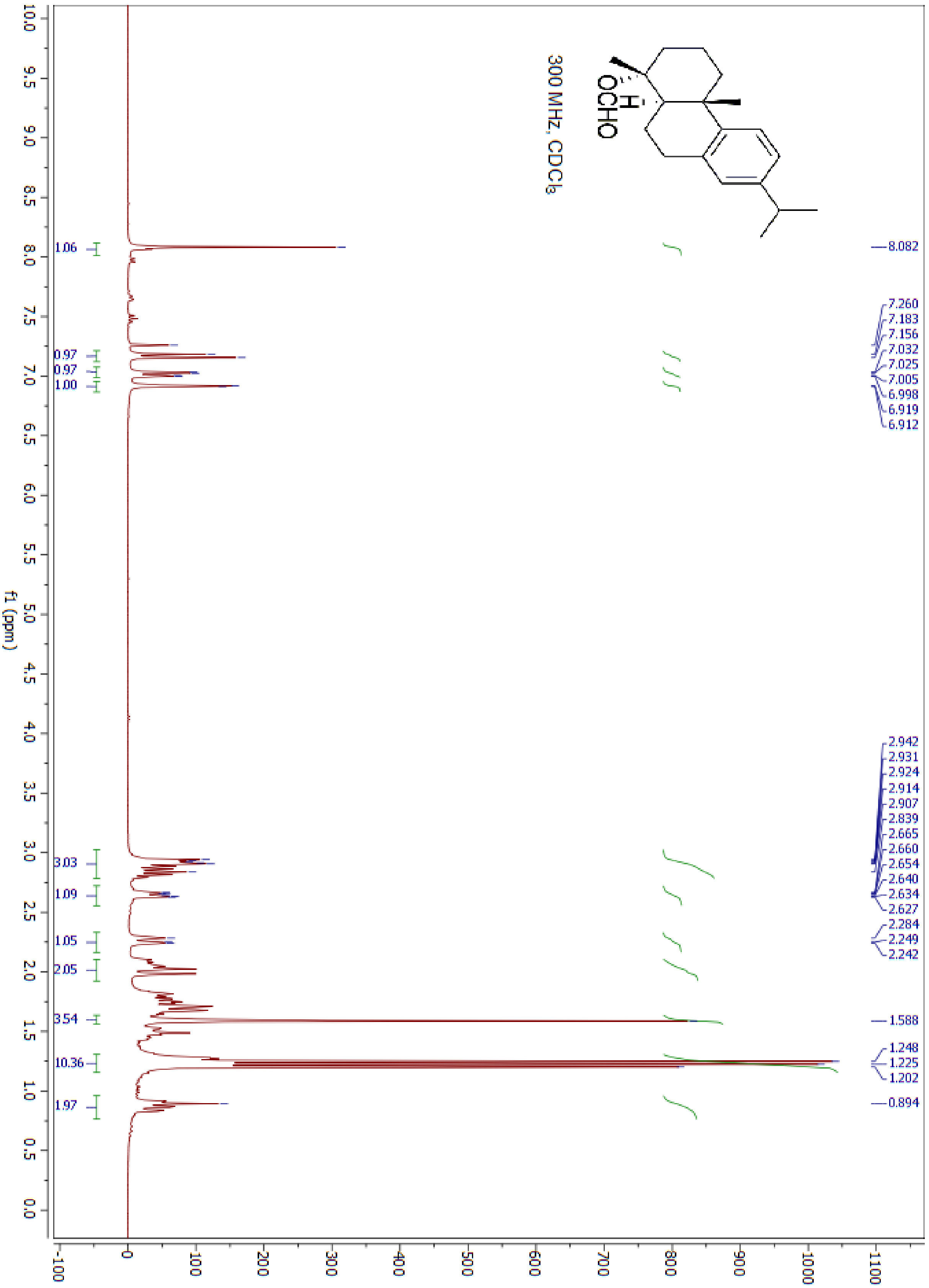
300 MHz, CDCl3
OCHO
H
8.082
7.260
7.183
7.156
7.032
7.025
7.005
6.998
6.919
6.912
2.942
2.931
2.924
2.914
2.907
2.839
2.665
2.660
2.654
2.640
2.634
2.627
2.284
2.249
2.242
1.588
1.248
1.225
1.202
0.894
1.06
0.97
0.97
1.00
3.03
1.09
1.05
2.05
3.54
10.36
1.97
f1 (ppm)

Compound 12

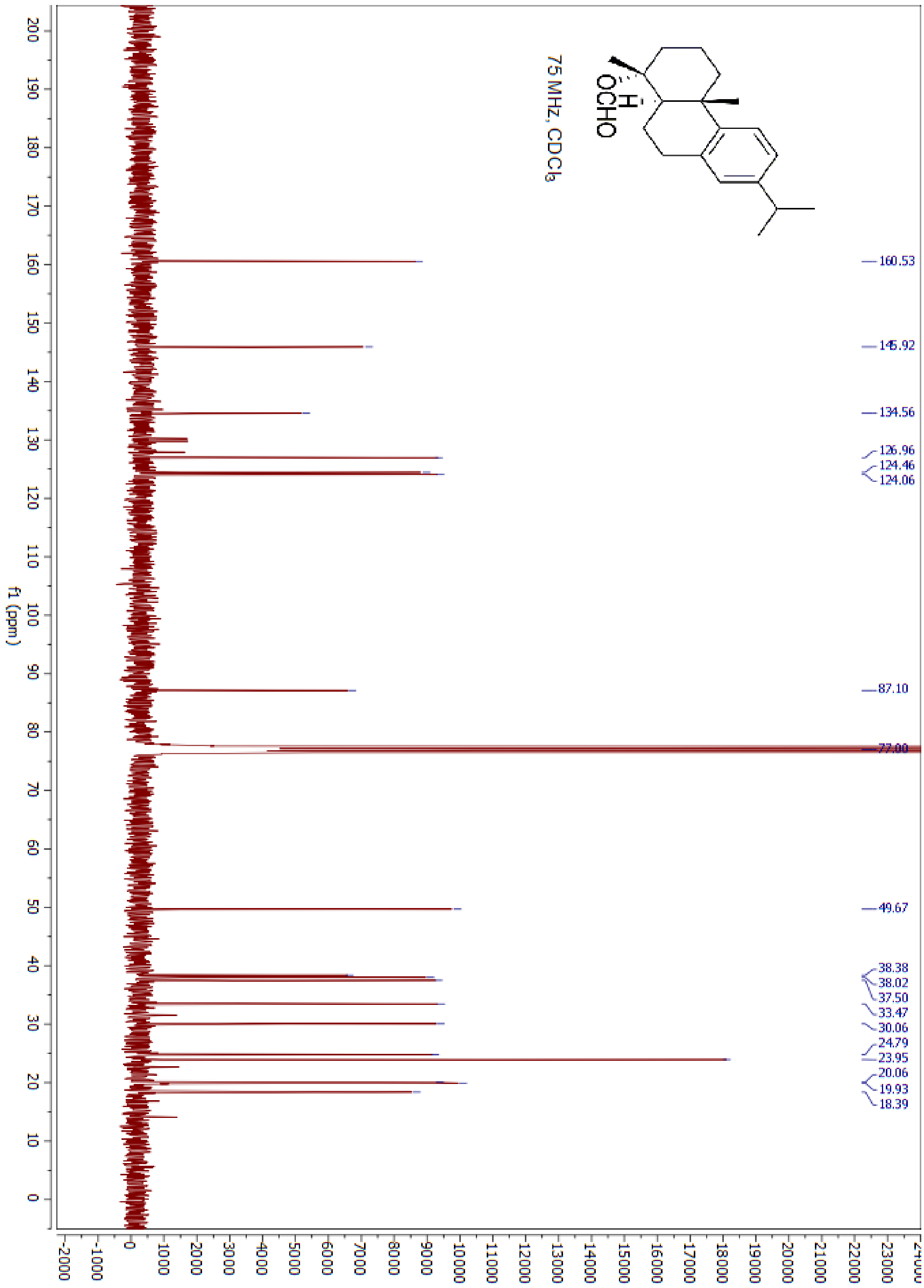
75 MHz, CDCl3
OCHO
H
160.53
145.92
134.56
126.96
124.46
124.06
87.10
77.00
49.67
38.38
38.02
37.50
33.47
30.06
24.79
23.95
20.06
19.93
18.39
f1 (ppm)

Compound 12

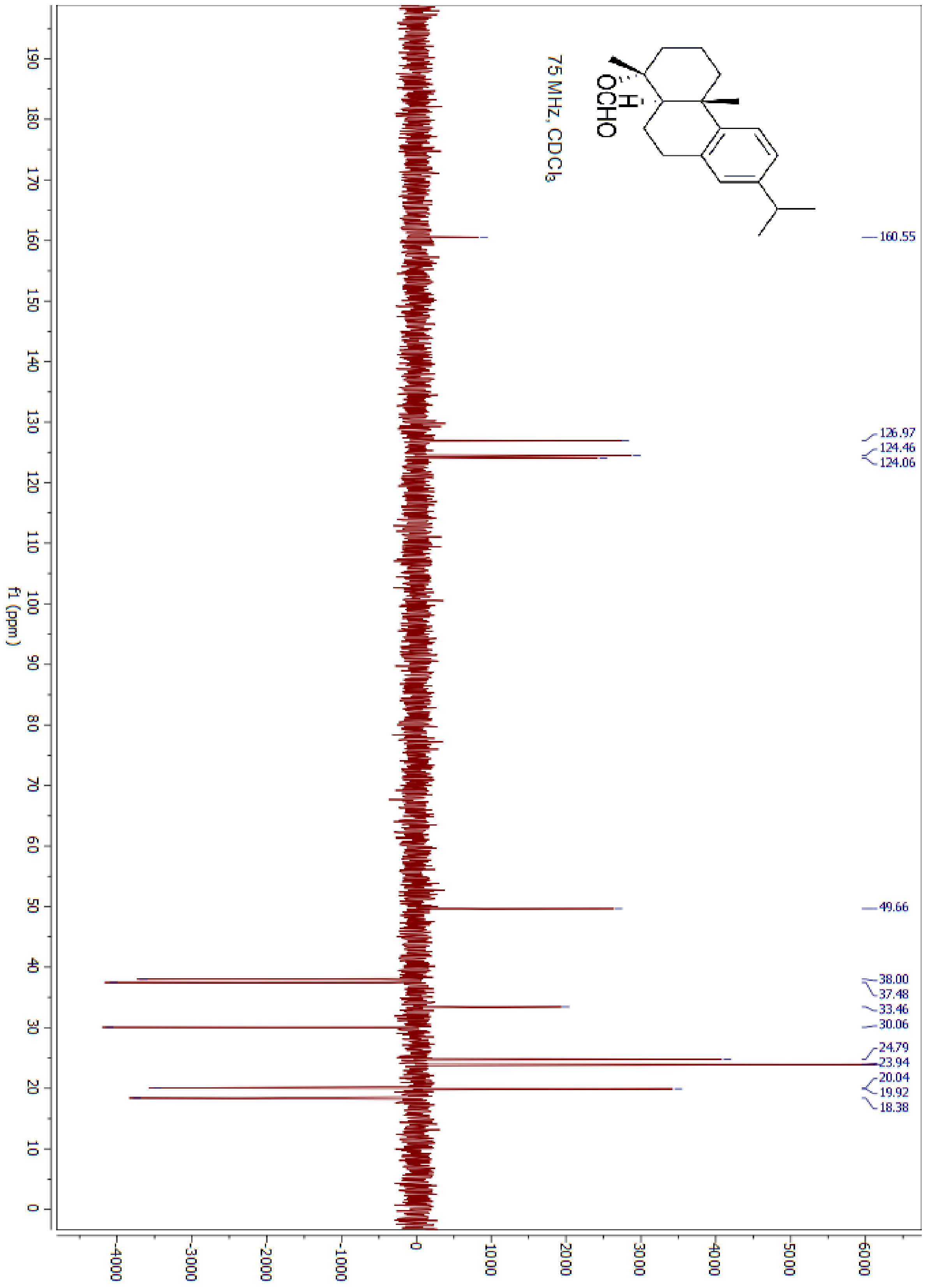

75 MHz, CDCl3
OCHO
H
160.55
126.97
124.46
124.06
49.66
38.00
37.48
33.46
30.06
24.79
23.94
20.04
19.92
18.38
f1 (ppm)

Compound 9

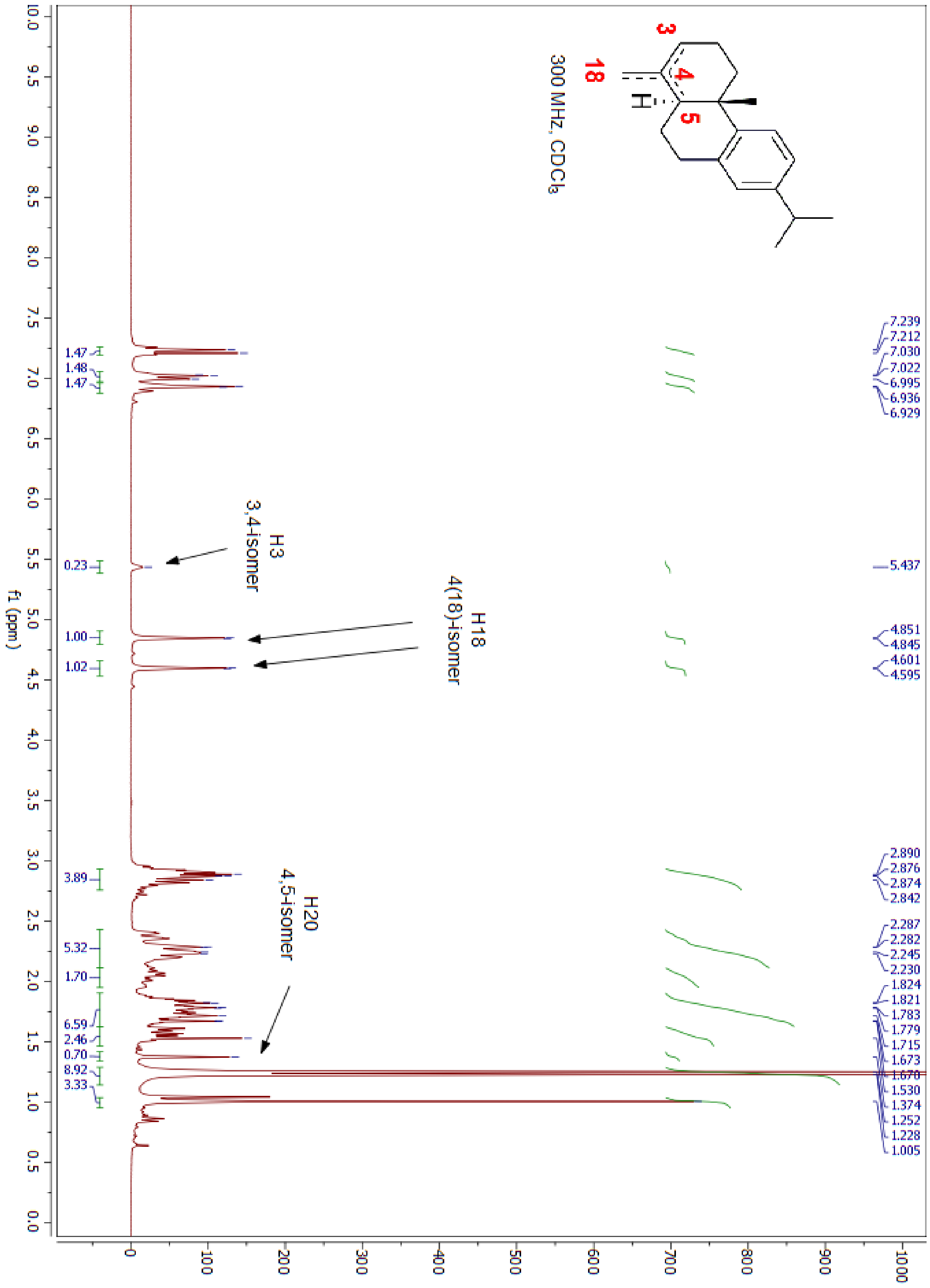
300 MHz, CDCl3
3
4
5
18
H
H3
3,4-isomer
H18
4(18)-isomer
H20
4,5-isomer
f1 (ppm)
7.239
7.212
7.030
7.022
6.995
6.936
6.929
5.437
4.851
4.845
4.601
4.595
2.890
2.876
2.874
2.842
2.287
2.282
2.245
2.230
1.824
1.821
1.783
1.779
1.715
1.673
1.670
1.530
1.374
1.252
1.228
1.005
1.47
1.48
1.47
0.23
1.00
1.02
3.89
5.32
1.70
6.59
2.46
0.70
8.92
3.33

Compound 9

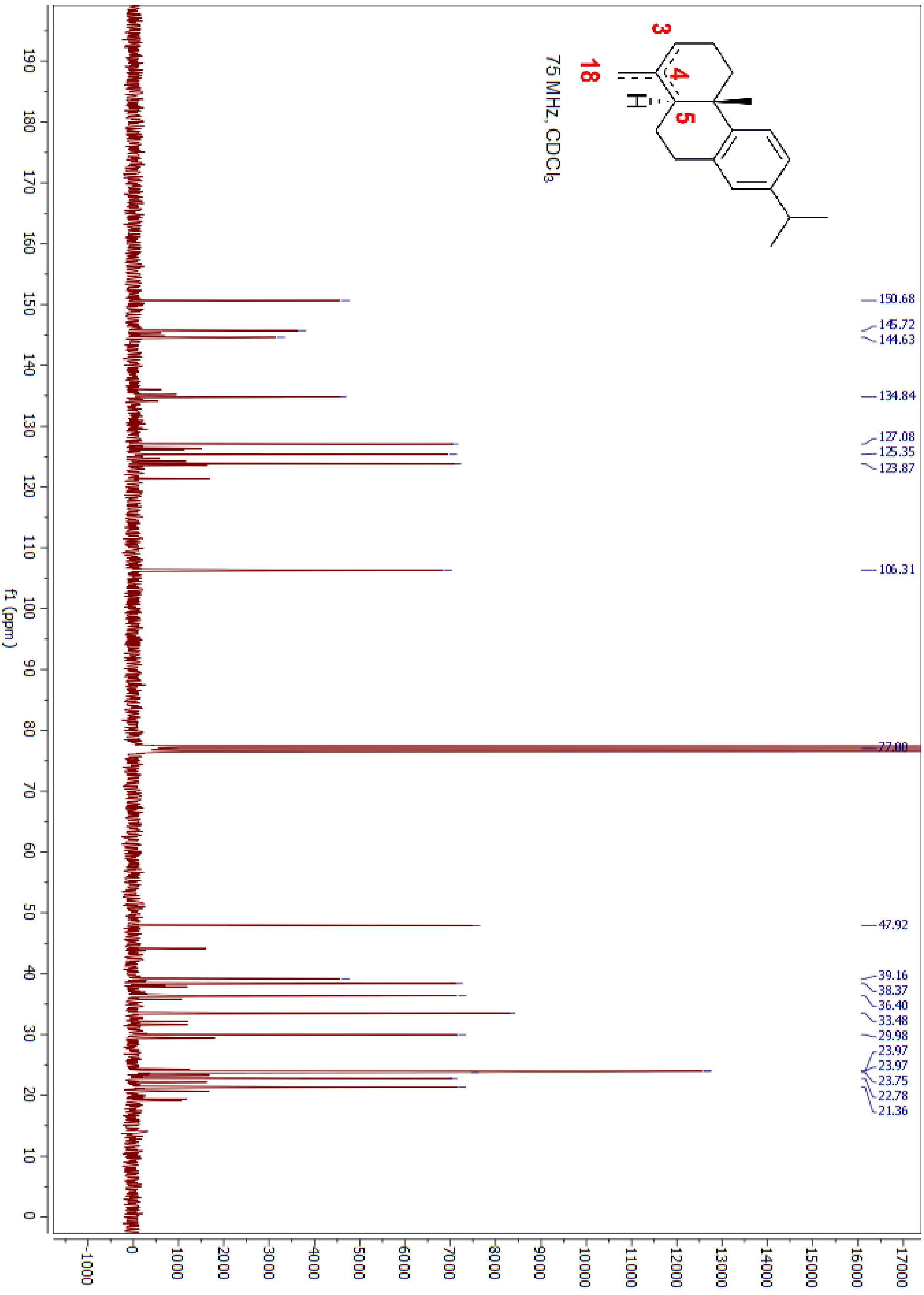

3
4
5
18
H
75 MHz, CDCl3
150.68
145.72
144.63
134.84
127.08
125.35
123.87
106.31
77.00
47.92
39.16
38.37
36.40
33.48
29.98
23.97
23.97
23.75
22.78
21.36
f1 (ppm)

Compound 9

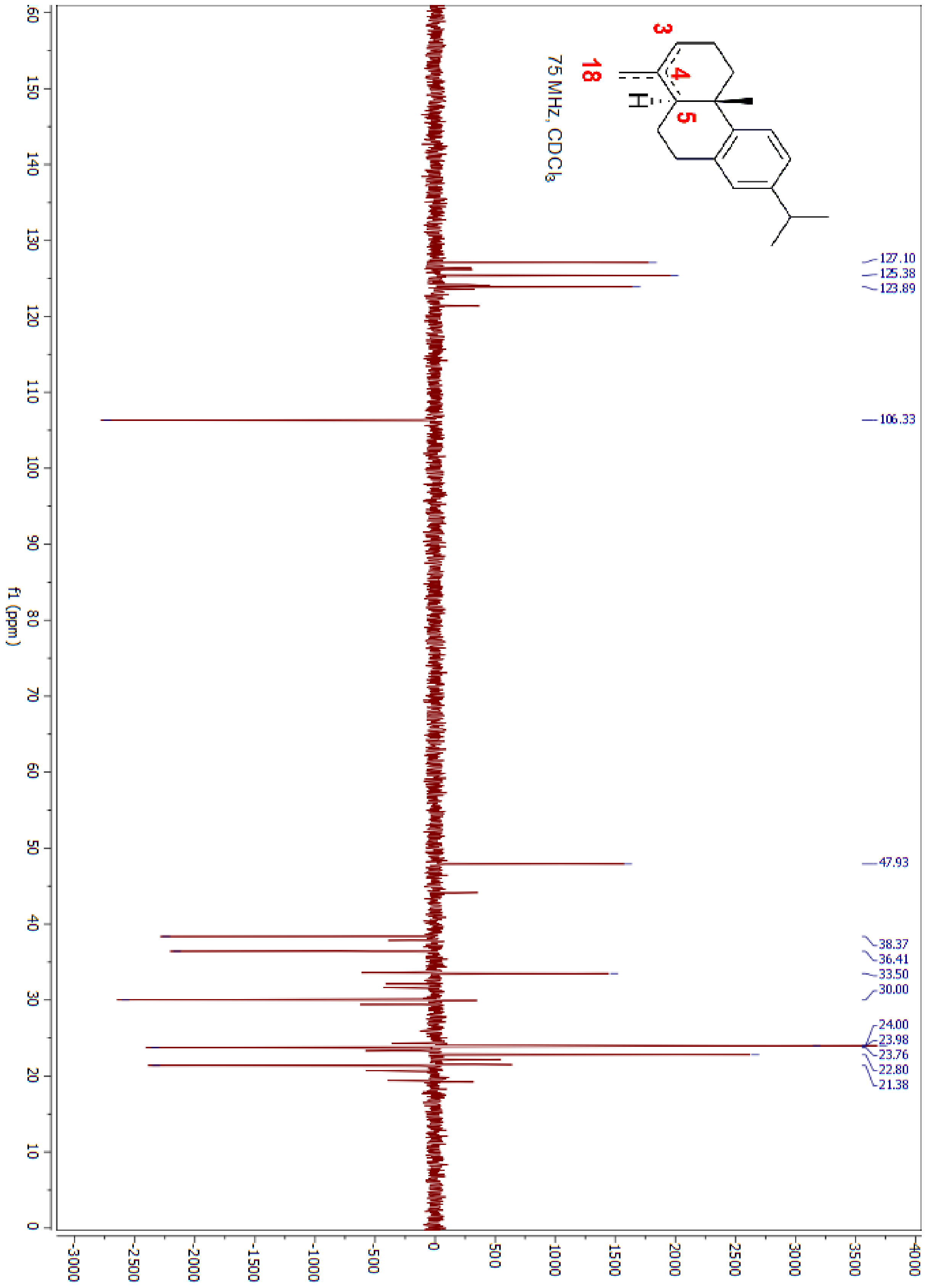

75 MHz, CDCl3
3
4
5
18
127.10
125.38
123.89
106.33
47.93
38.37
36.41
33.50
30.00
24.00
23.98
23.76
22.80
21.38
f1 (ppm)

Compound 8

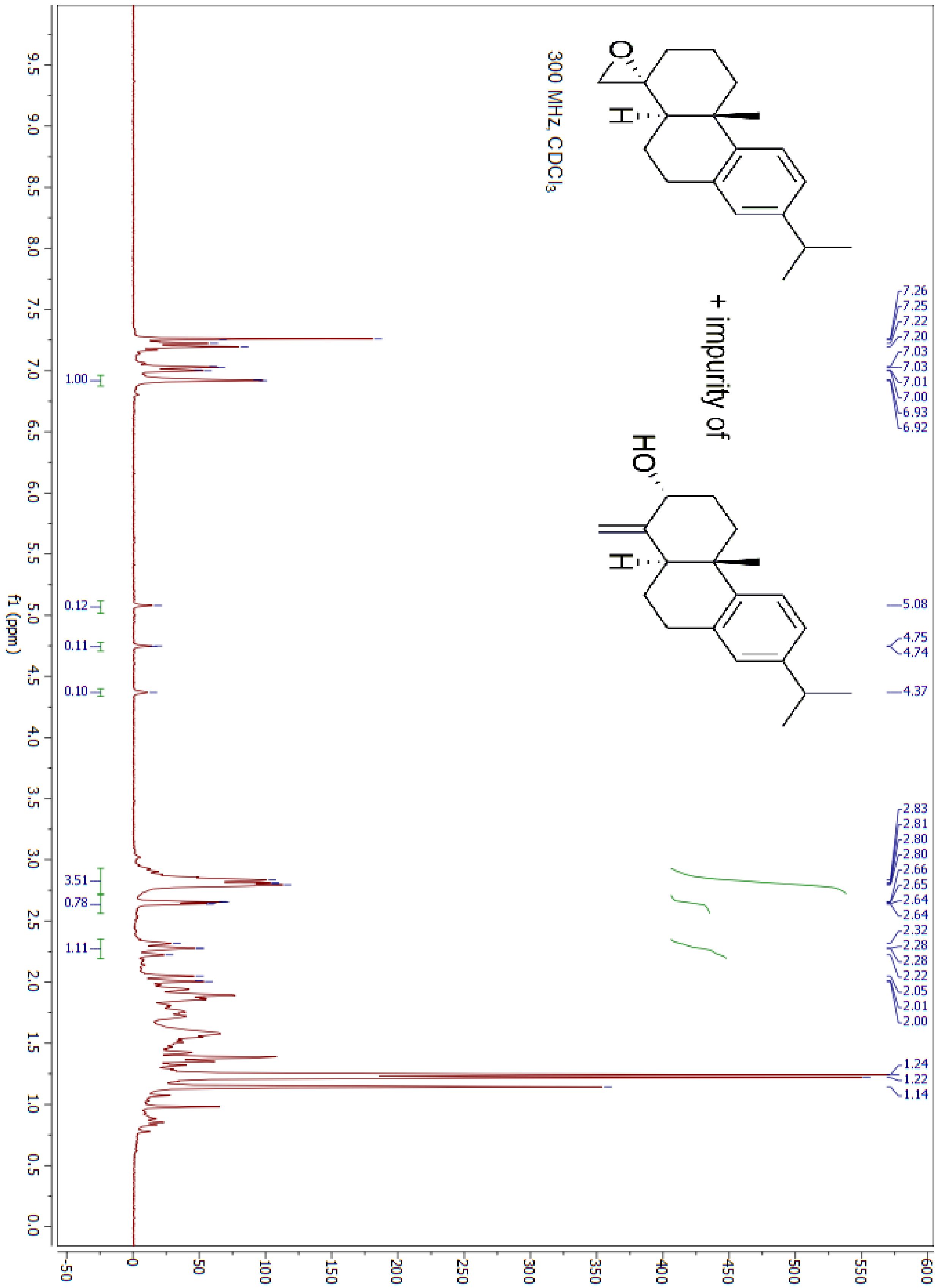

Compounds 7 (ca. 1:1 α/β aldehyde)

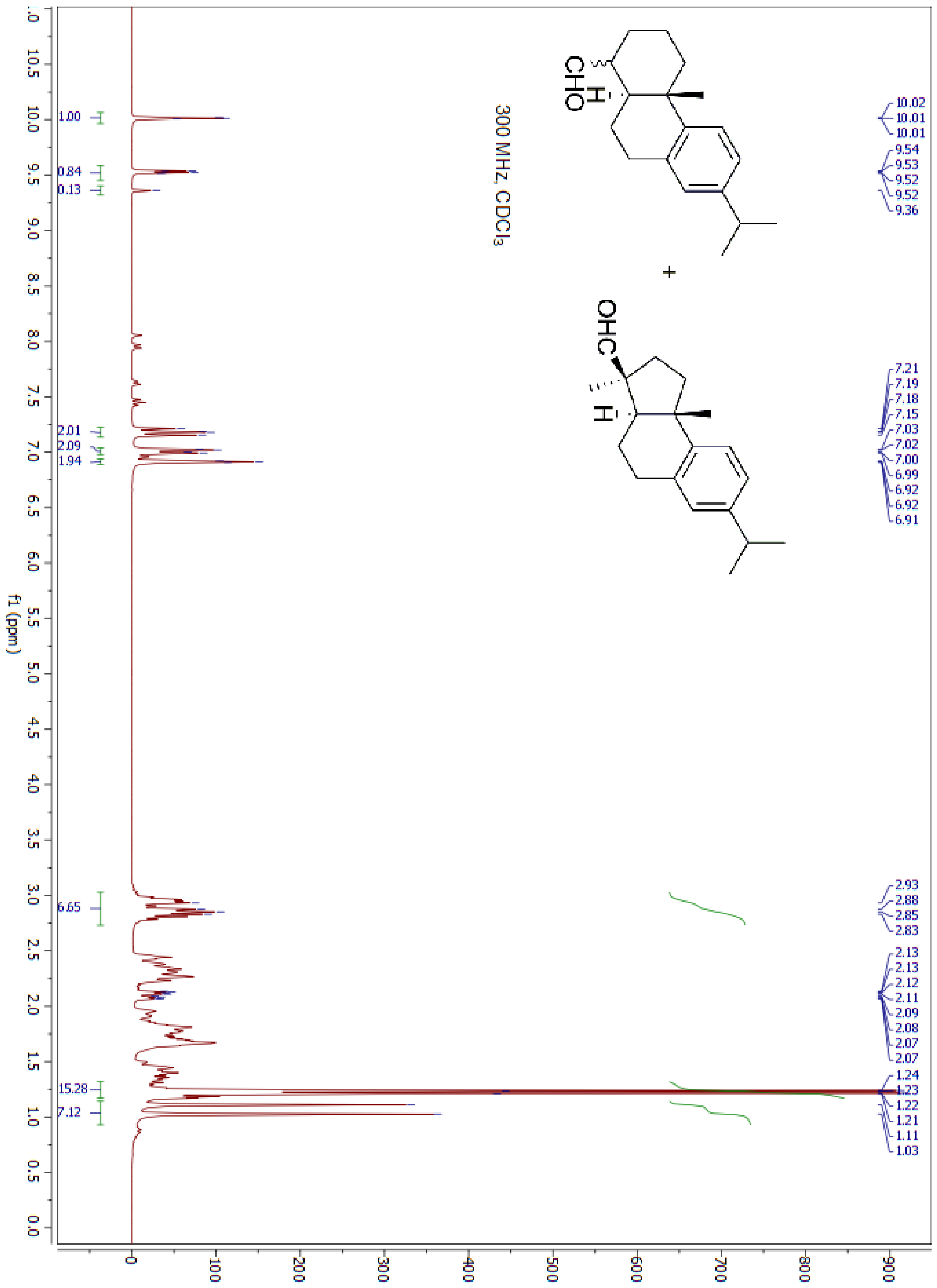

Compound 13

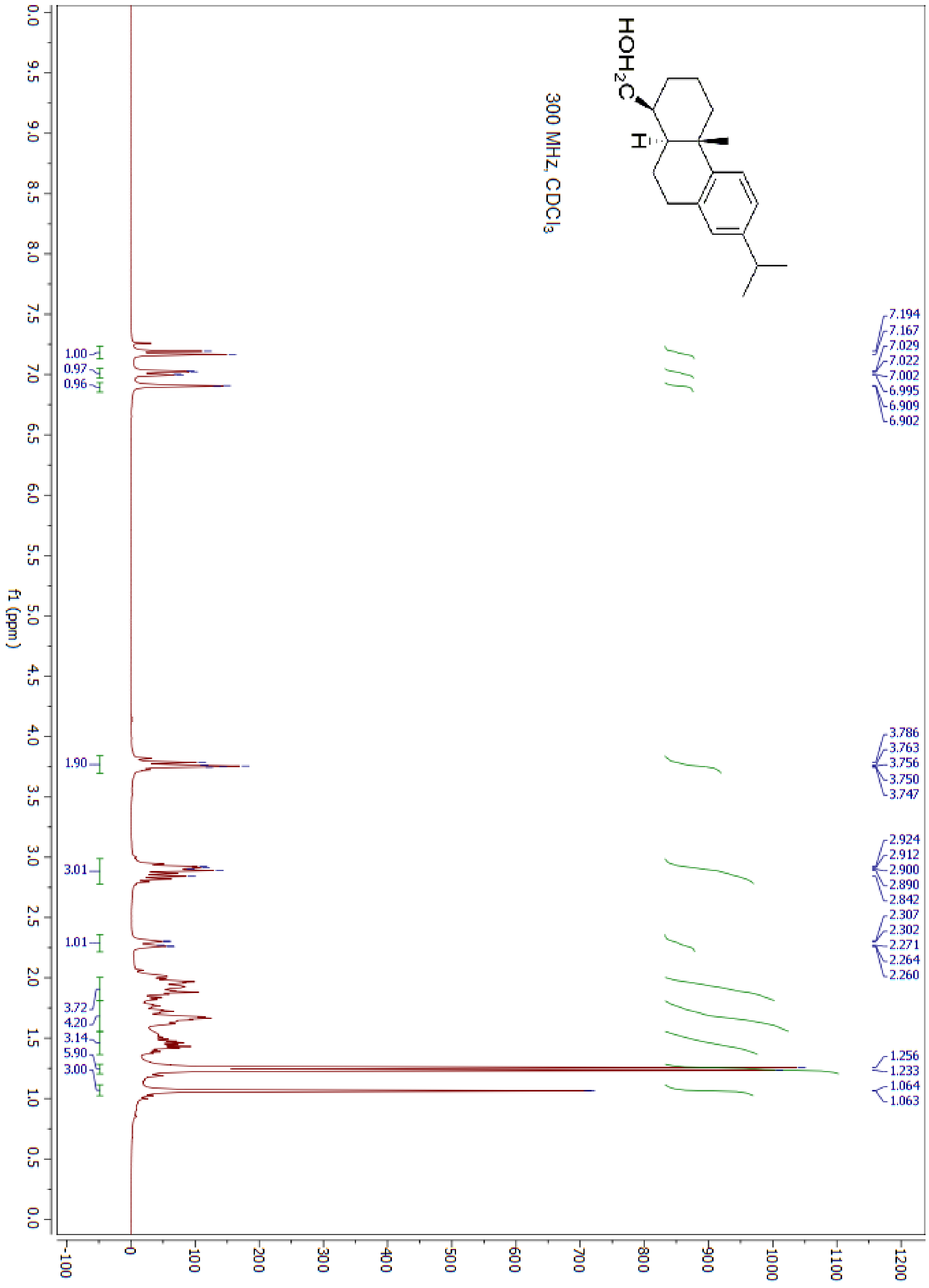
300 MHz, CDCl3
HOH2C
H
f1 (ppm)
7.194
7.167
7.029
7.022
7.002
6.995
6.909
6.902
3.786
3.763
3.756
3.750
3.747
2.924
2.912
2.900
2.890
2.842
2.307
2.302
2.271
2.264
2.260
1.256
1.233
1.064
1.063
1.00
0.97
0.96
1.90
3.01
1.01
3.72
4.20
3.14
5.90
3.00

Compound 13

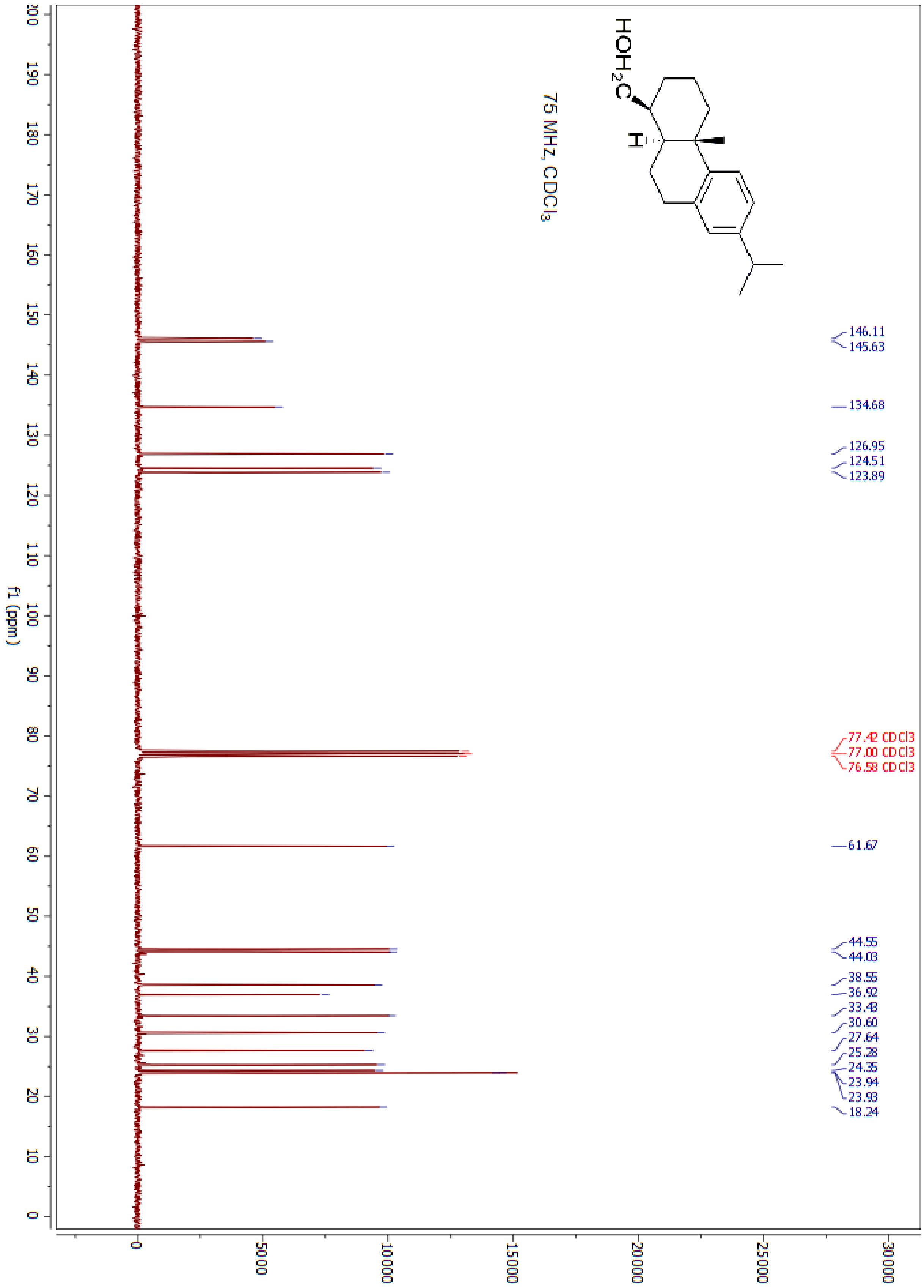

75 MHz, CDCl3
HOH2C
H
146.11
145.63
134.68
126.95
124.51
123.89
77.42 CDCl3
77.00 CDCl3
76.58 CDCl3
61.67
44.55
44.03
38.55
36.92
33.43
30.60
27.64
25.28
24.35
23.94
23.93
18.24
f1 (ppm)

Compound 13

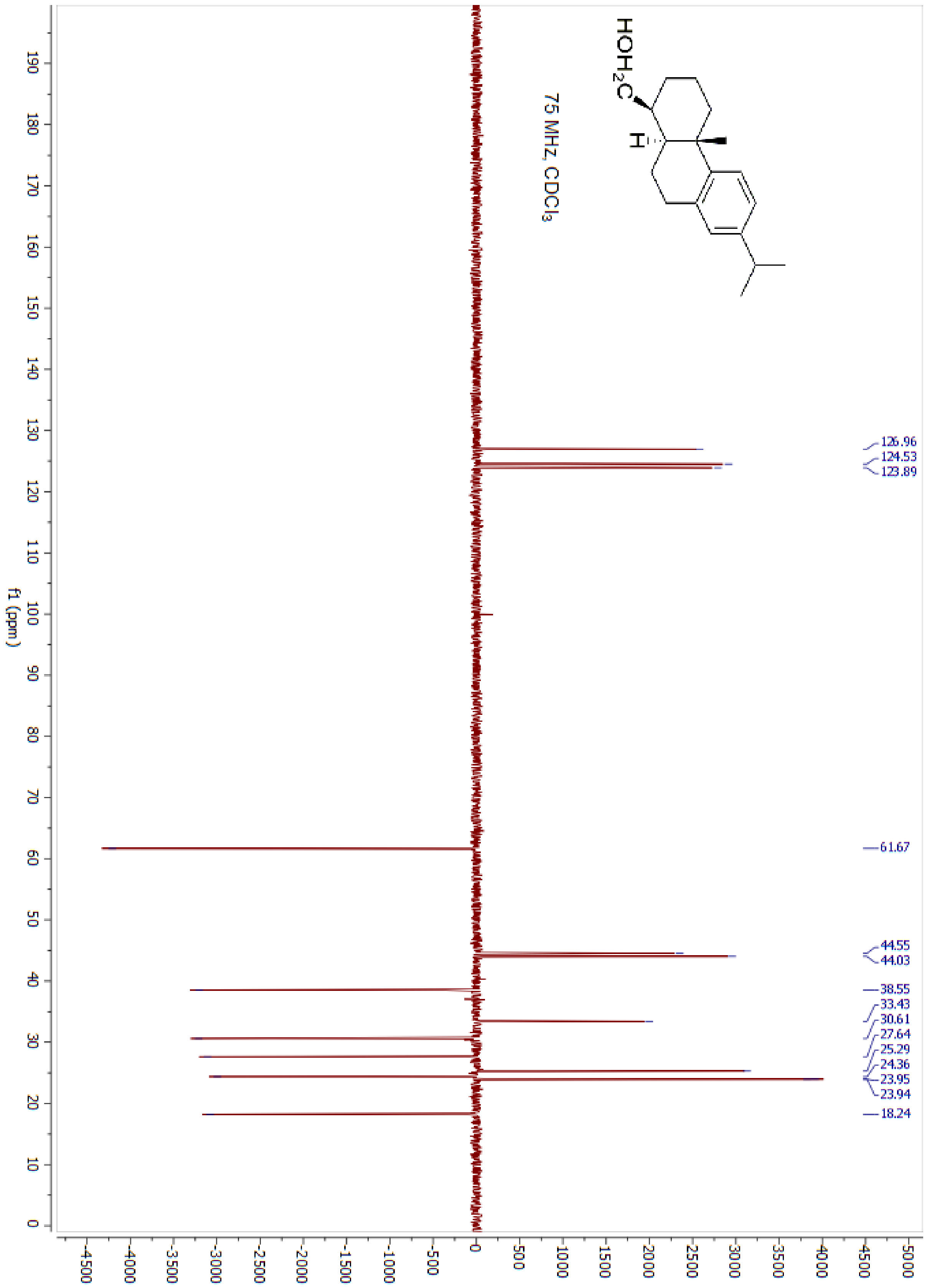
HOH2C
H
75 MHz, CDCl3
126.96
124.53
123.89
61.67
44.55
44.03
38.55
33.43
30.61
27.64
25.29
24.36
23.95
23.94
18.24
f1 (ppm)

Compound 7a

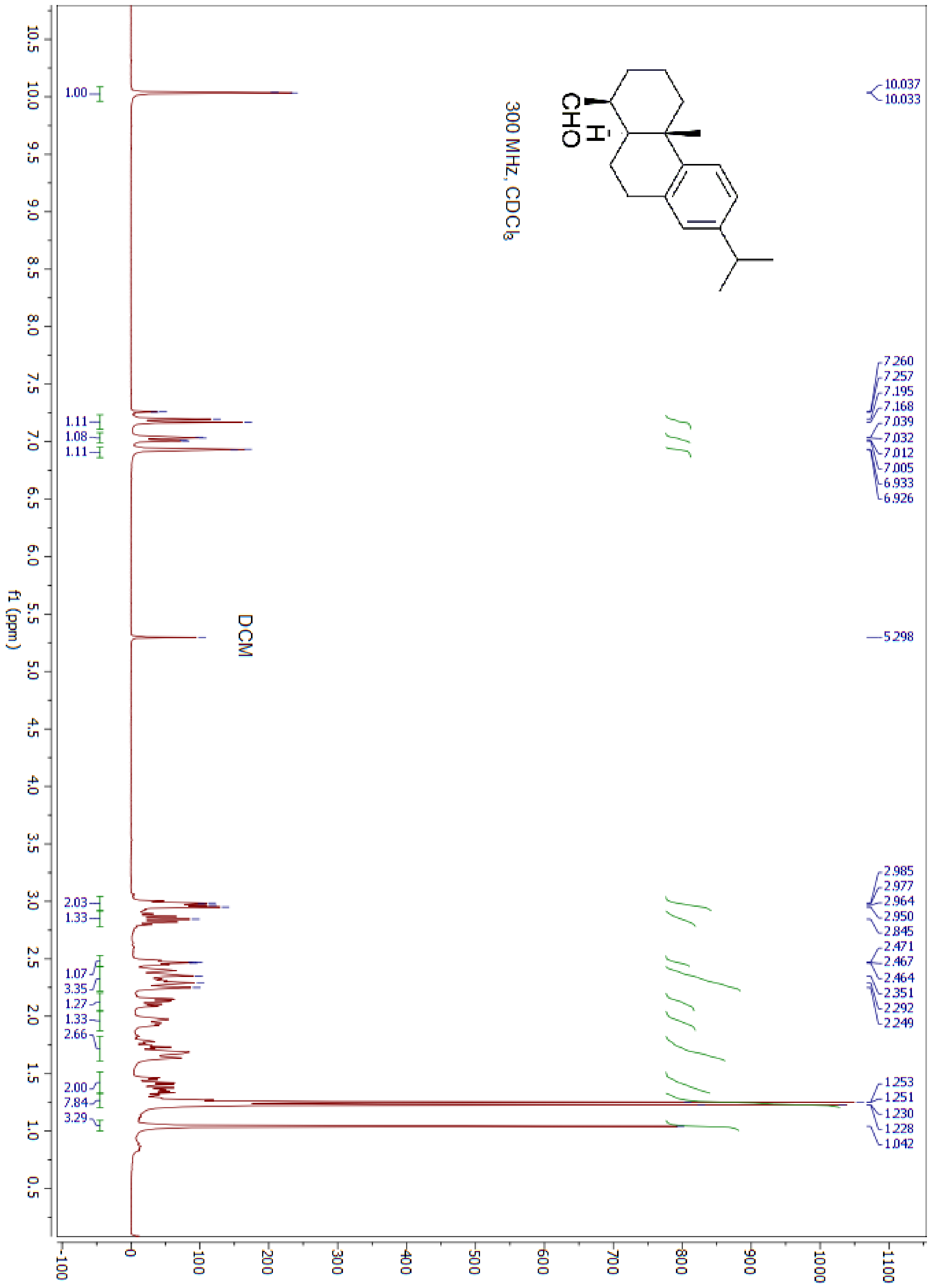

300 MHz, CDCl3
CHO
H
DCM
f1 (ppm)
10.037
10.033
7.260
7.257
7.195
7.168
7.039
7.032
7.012
7.005
6.933
6.926
5.298
2.985
2.977
2.964
2.950
2.845
2.471
2.467
2.464
2.351
2.292
2.249
1.253
1.251
1.230
1.228
1.042
1.00
1.11
1.08
1.11
2.03
1.33
1.07
3.35
1.27
1.33
2.66
2.00
7.84
3.29

Compound 7a

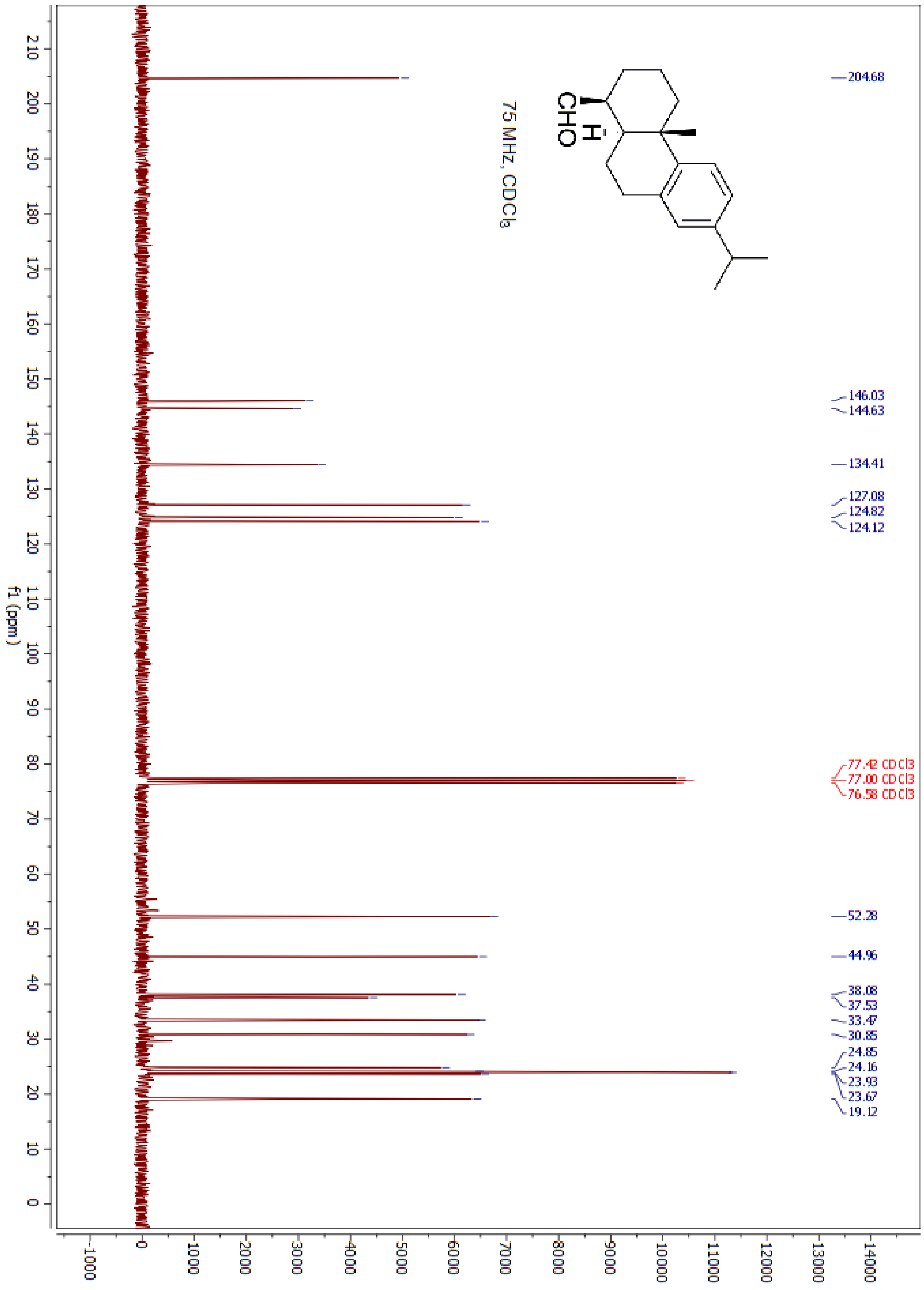

75 MHz, CDCl3
CHO
H
204.68
146.03
144.63
134.41
127.08
124.82
124.12
77.42 CDCl3
77.00 CDCl3
76.58 CDCl3
52.28
44.96
38.08
37.53
33.47
30.85
24.85
24.16
23.93
23.67
19.12
f1 (ppm)

Compound 7a

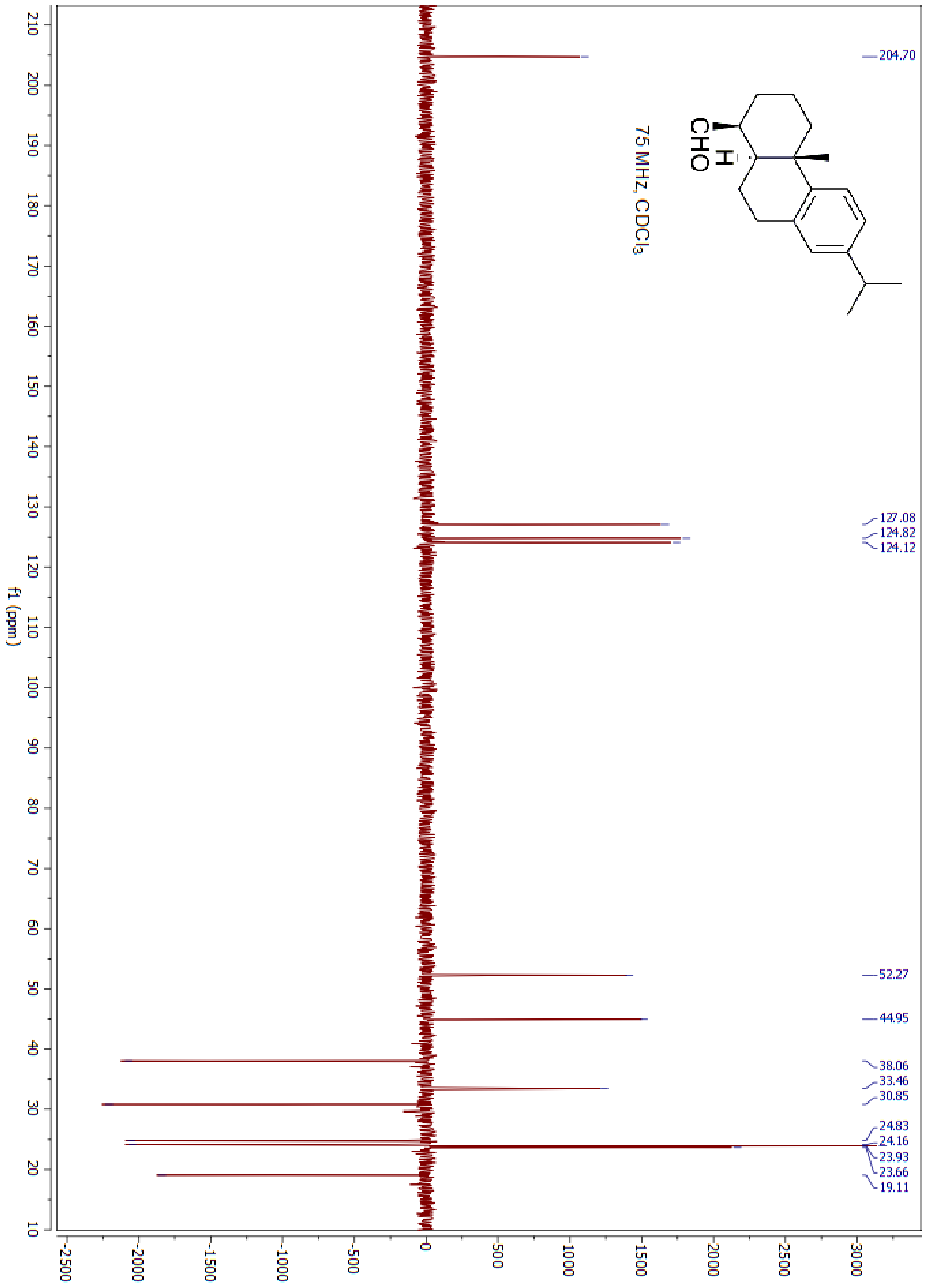

75 MHz, CDCl3
CHO
H
204.70
127.08
124.82
124.12
52.27
44.95
38.06
33.46
30.85
24.83
24.16
23.93
23.66
19.11
f1 (ppm)

Compound 6

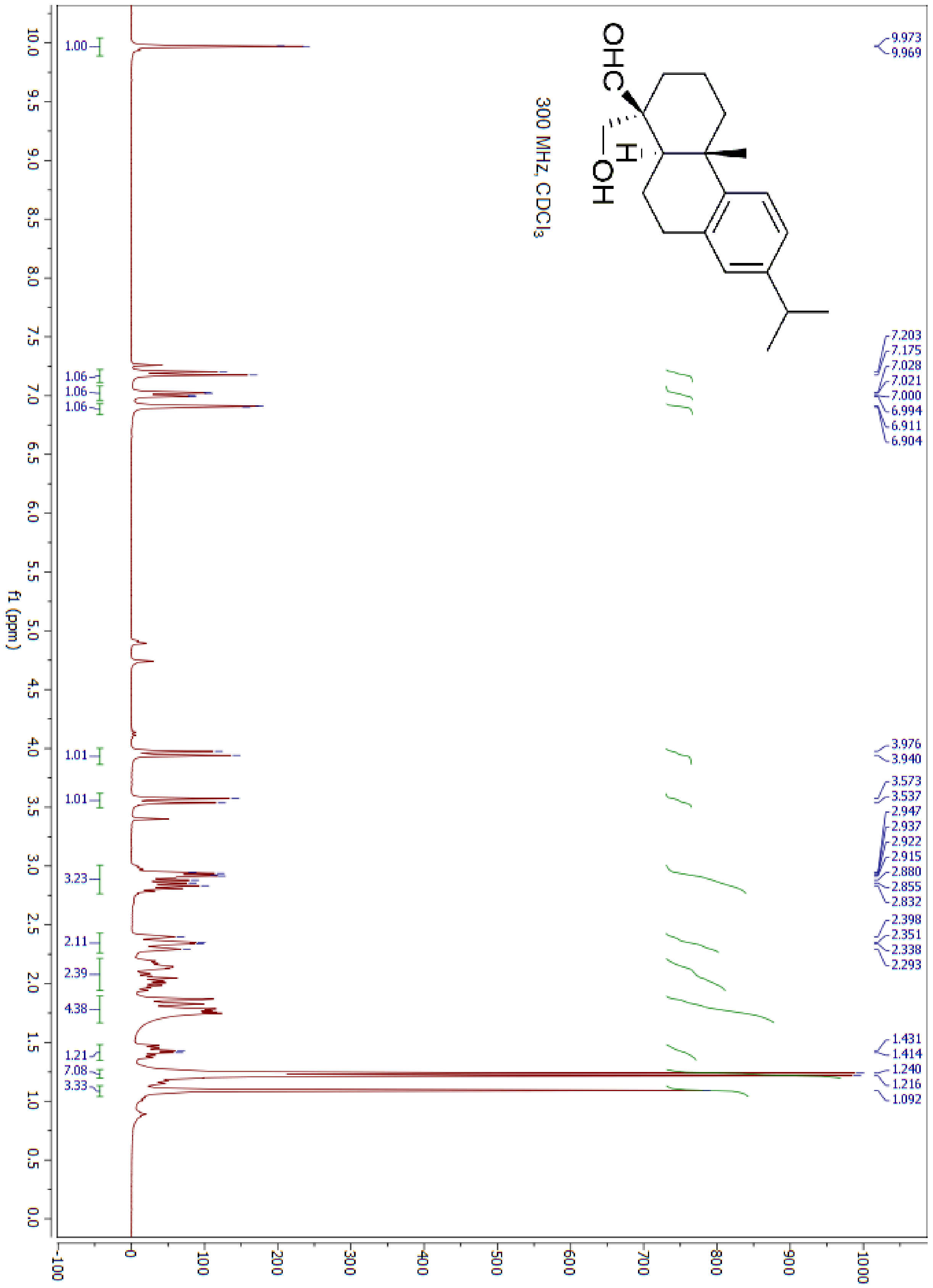
300 MHz, CDCl3
OHC
H
OH
f1 (ppm)
9.973
9.969
7.203
7.175
7.028
7.021
7.000
6.994
6.911
6.904
3.976
3.940
3.573
3.537
2.947
2.937
2.922
2.915
2.880
2.855
2.832
2.398
2.351
2.338
2.293
1.431
1.414
1.240
1.216
1.092
1.00
1.06
1.06
1.06
1.01
1.01
3.23
2.11
2.39
4.38
1.21
7.08
3.33

Compound 6

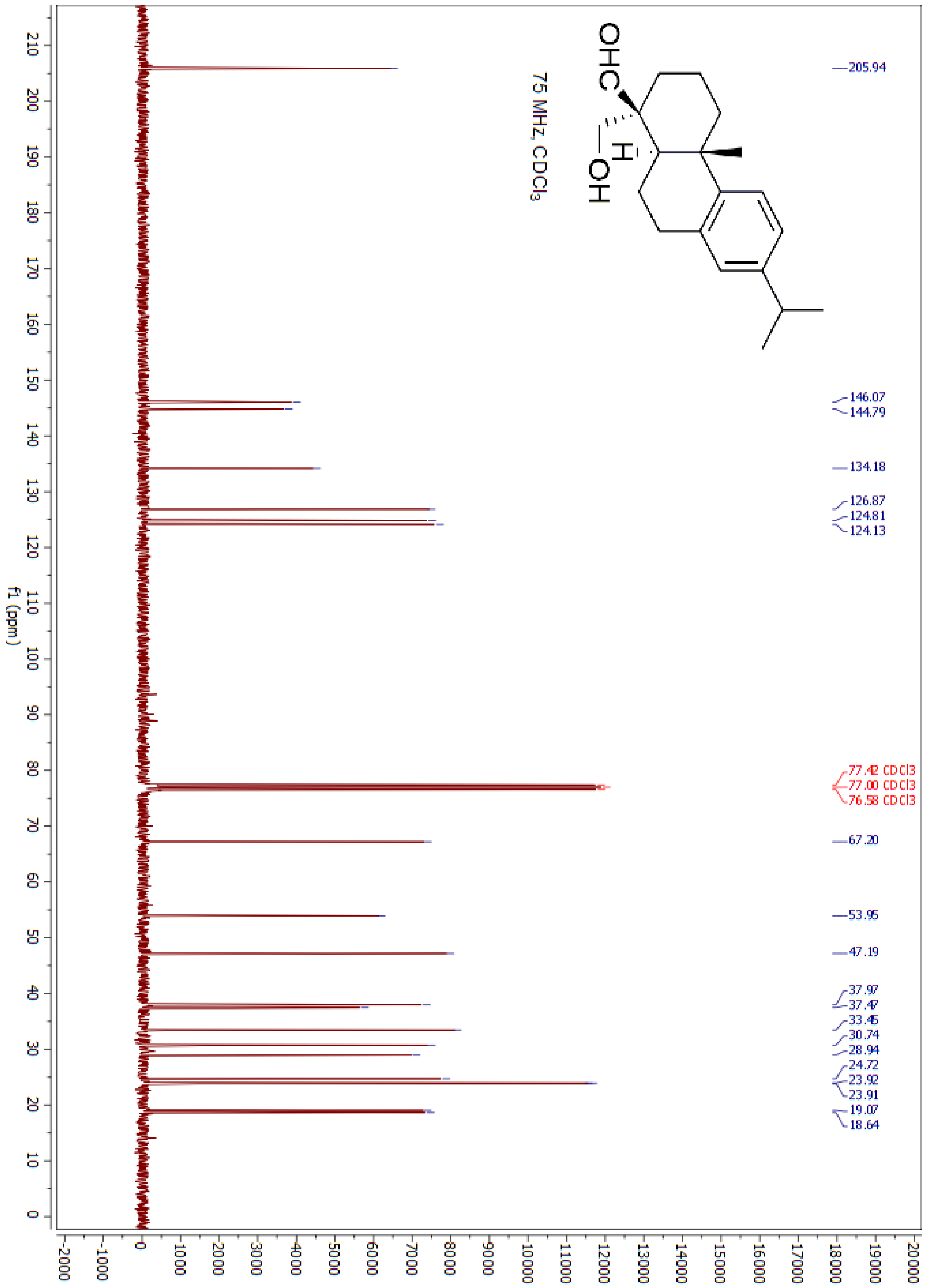
75 MHz, CDCl3
OHC
H
OH
205.94
146.07
144.79
134.18
126.87
124.81
124.13
77.42 CDCl3
77.00 CDCl3
76.58 CDCl3
67.20
53.95
47.19
37.97
37.47
33.45
30.74
28.94
24.72
23.92
23.91
19.07
18.64
f1 (ppm)

Compound 6

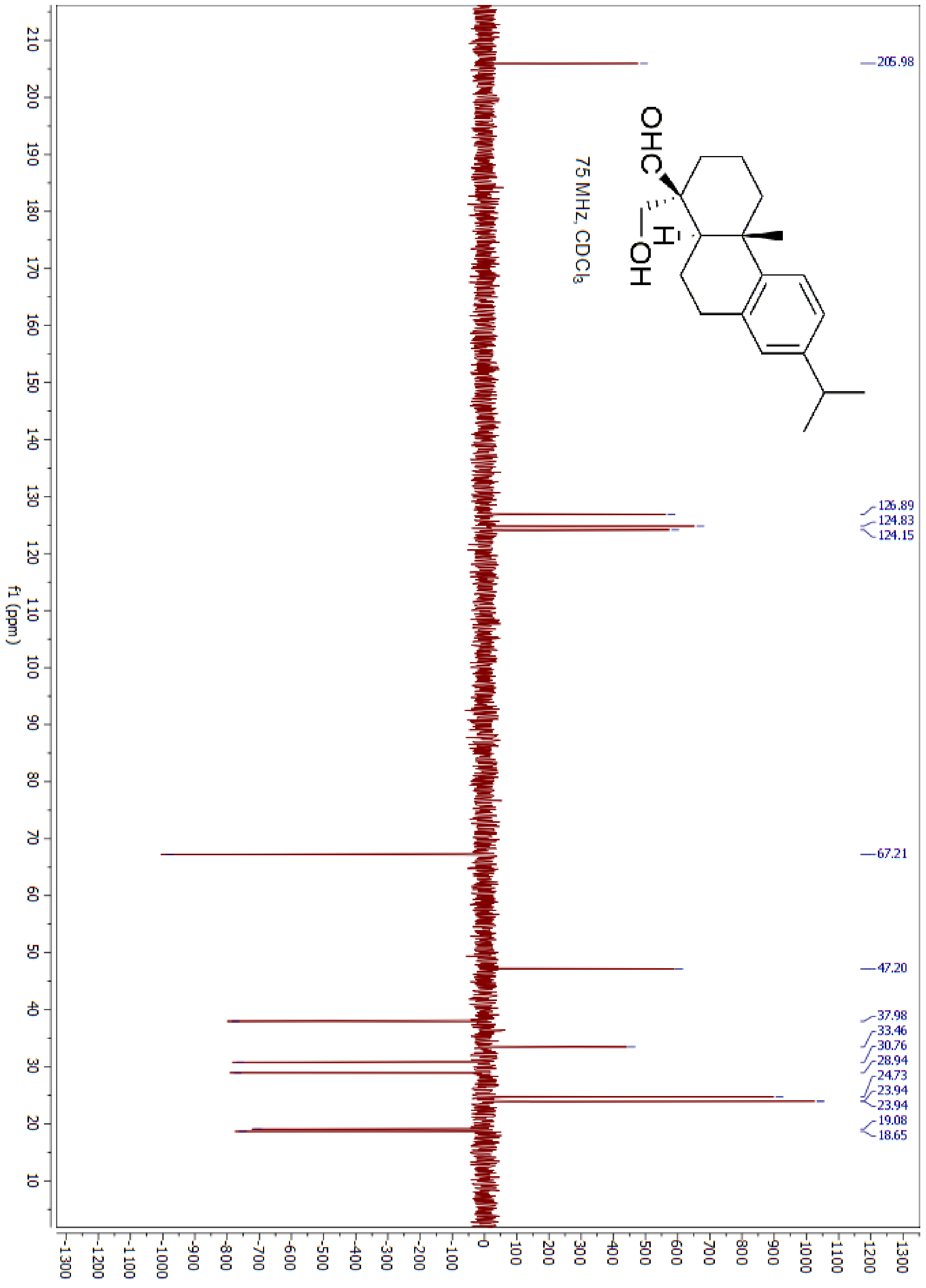

75 MHz, CDCl3
OHC
H
OH
205.98
126.89
124.83
124.15
67.21
47.20
37.98
33.46
30.76
28.94
24.73
23.94
23.94
19.08
18.65
f1 (ppm)

Compound 7b

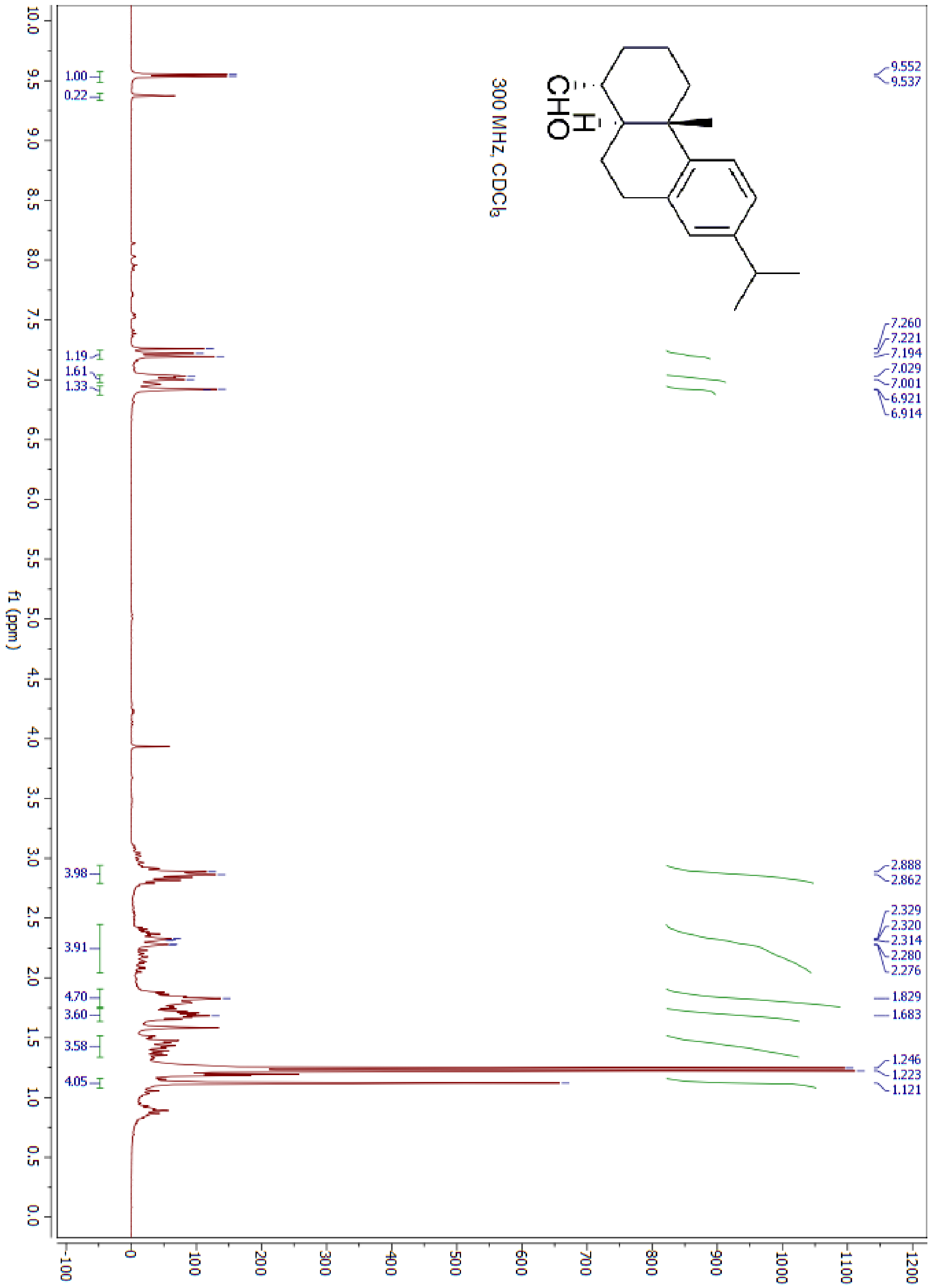
300 MHz, CDCl3
CHO
H
9.552
9.537
7.260
7.221
7.194
7.029
7.001
6.921
6.914
2.888
2.862
2.329
2.320
2.314
2.280
2.276
1.829
1.683
1.246
1.223
1.121
1.00
0.22
1.19
1.61
1.33
3.98
3.91
4.70
3.60
3.58
4.05
f1 (ppm)

Compound 7b

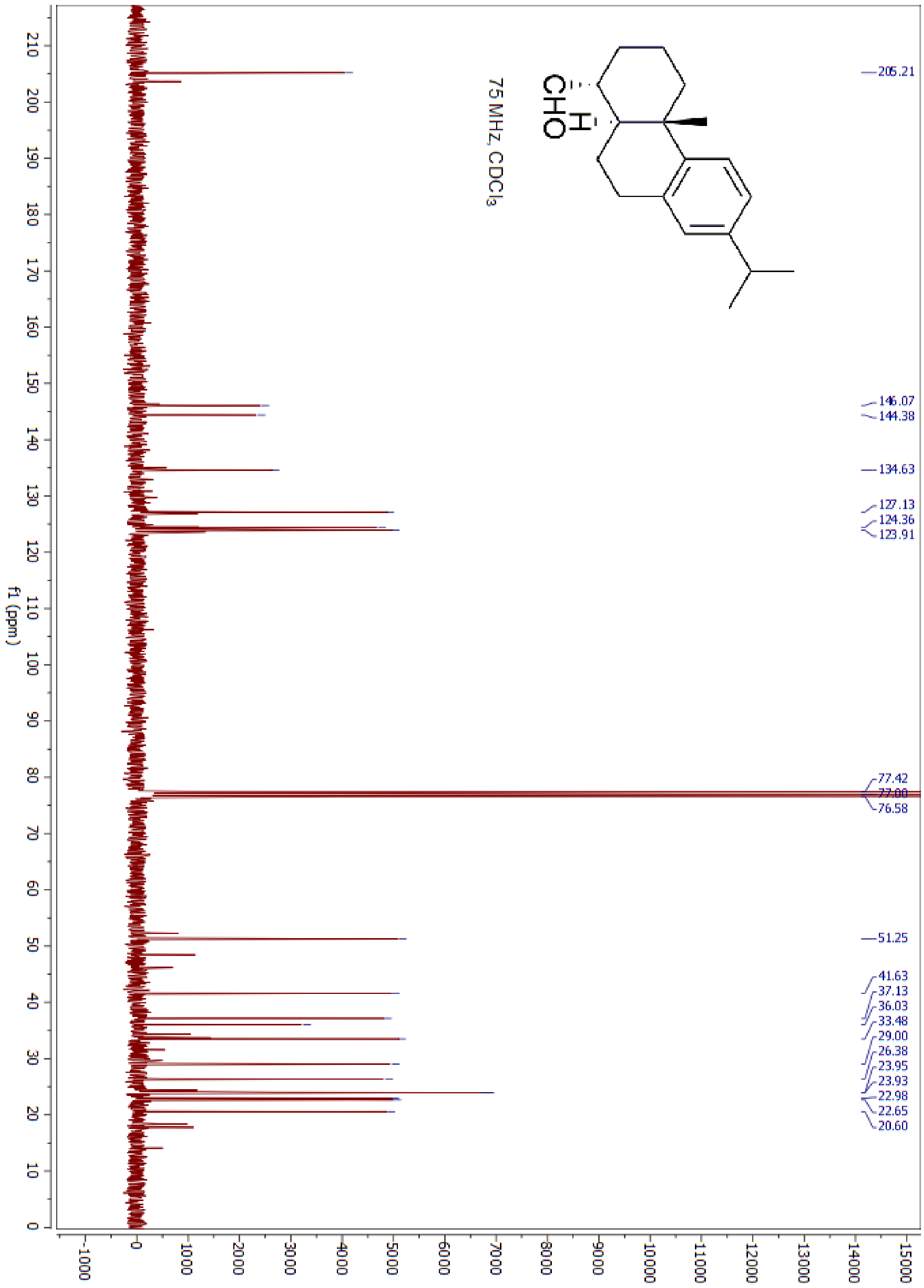

75 MHz, CDCl3
CHO
H
205.21
146.07
144.38
134.63
127.13
124.36
123.91
77.42
77.00
76.58
51.25
41.63
37.13
36.03
33.48
29.00
26.38
23.95
23.93
22.98
22.65
20.60
f1 (ppm)

Compound 7b

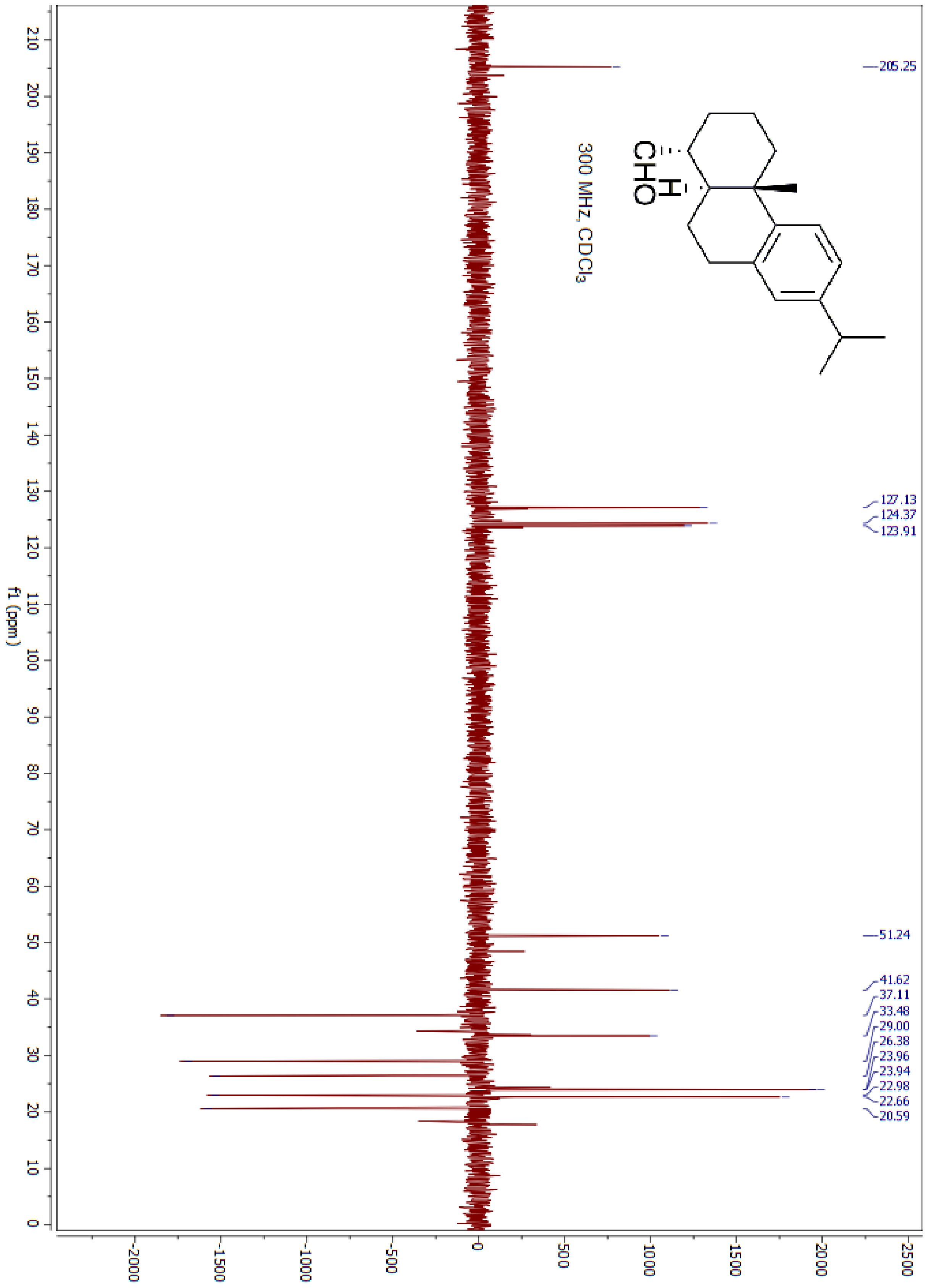

CHO
H
300 MHz, CDCl3
205.25
127.13
124.37
123.91
51.24
41.62
37.11
33.48
29.00
26.38
23.96
23.94
22.98
22.66
20.59
f1 (ppm)

Compound 4 (acetone-d6)

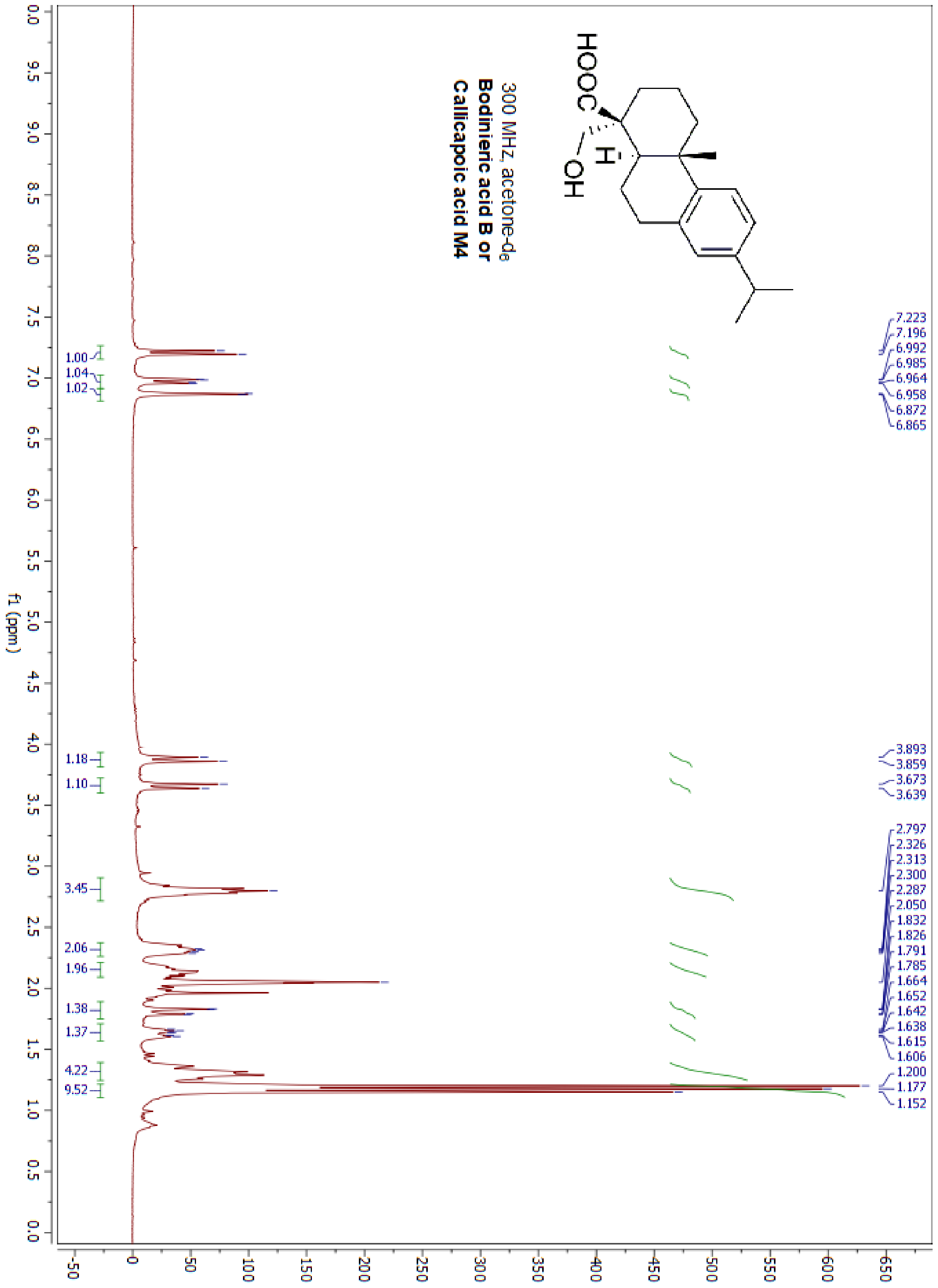

Compound 4 (acetone-d6)

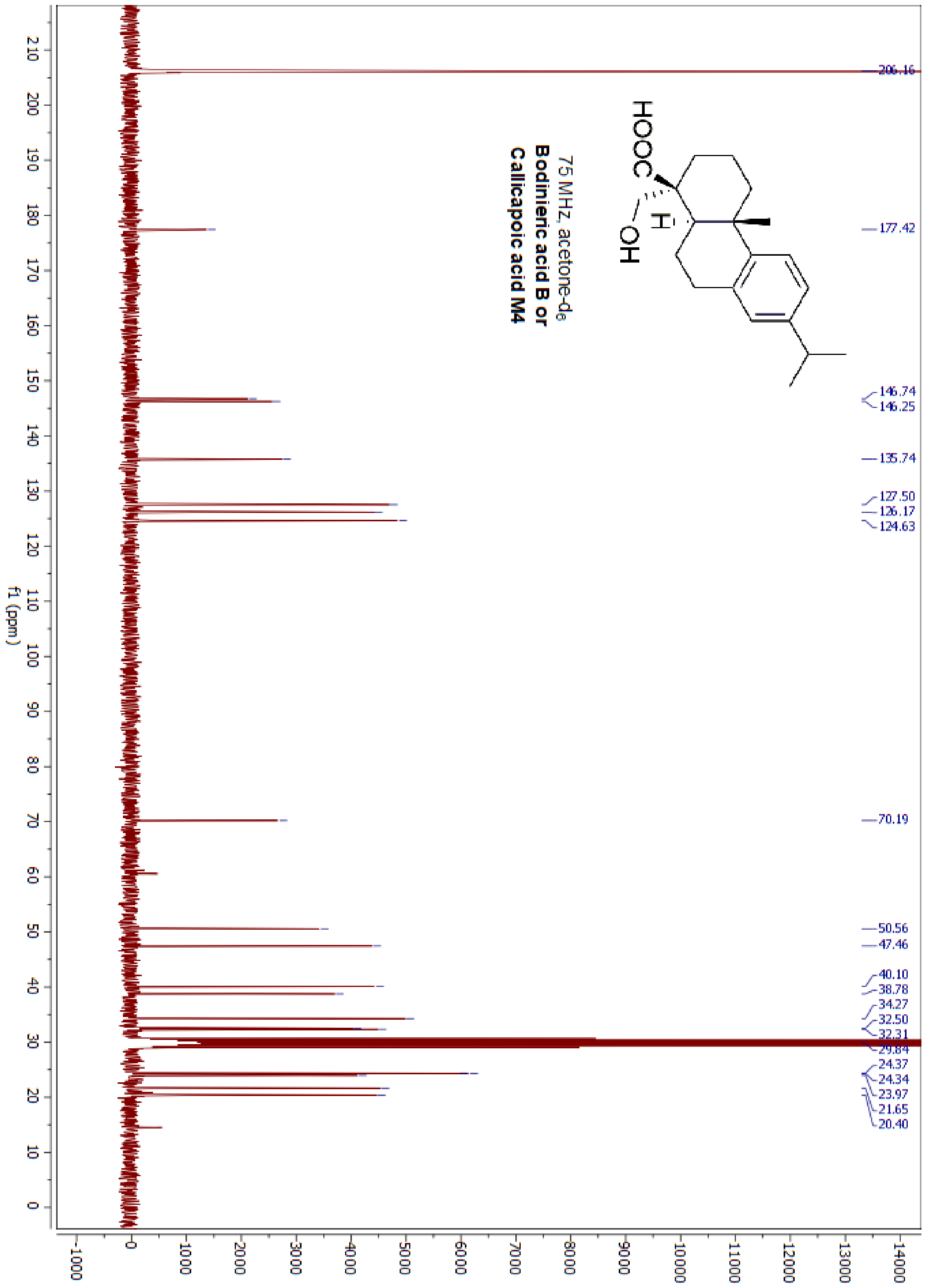

Compound 4 (acetone-d6)

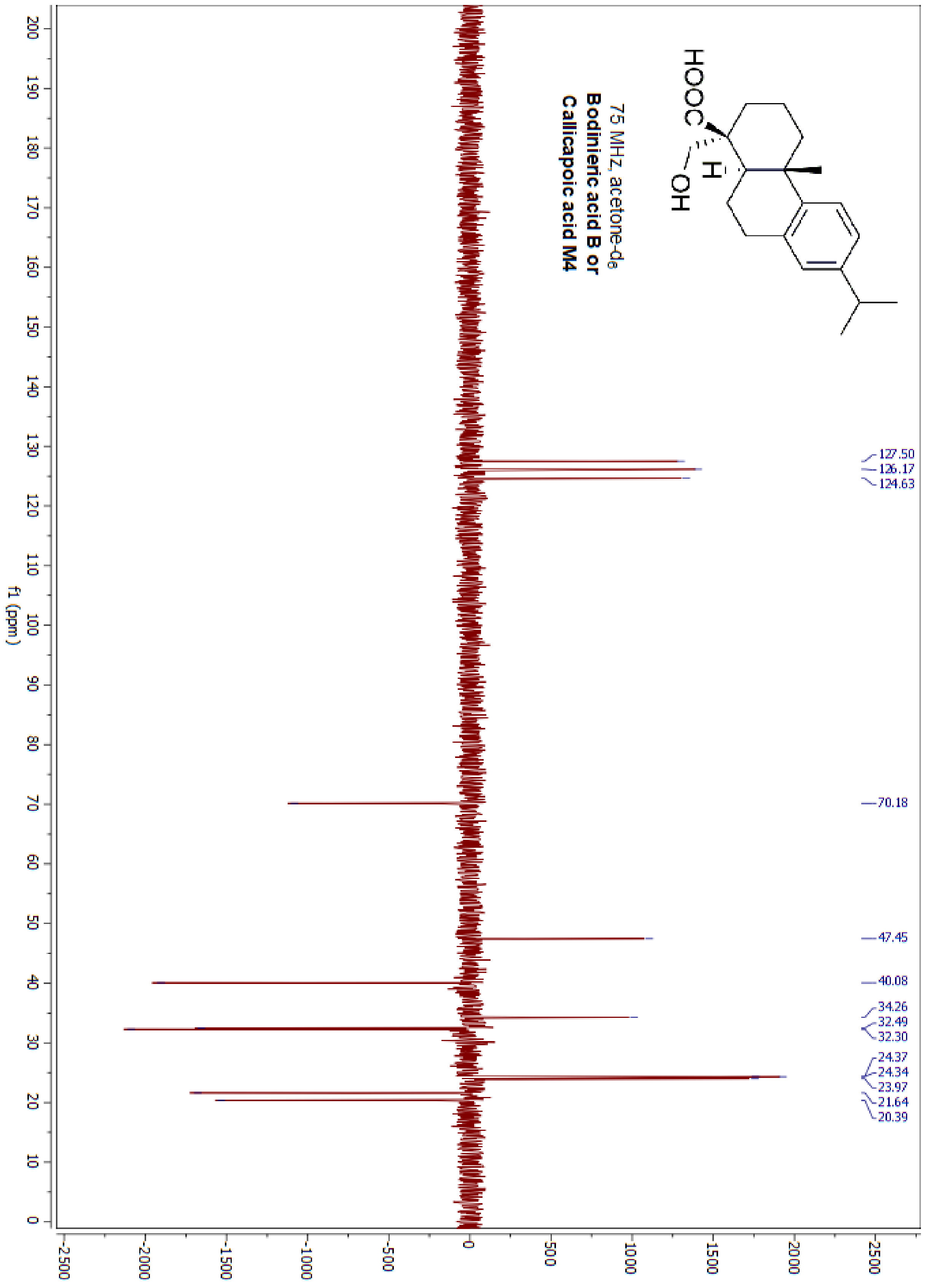

Compound 4 ($CDCl_3$)

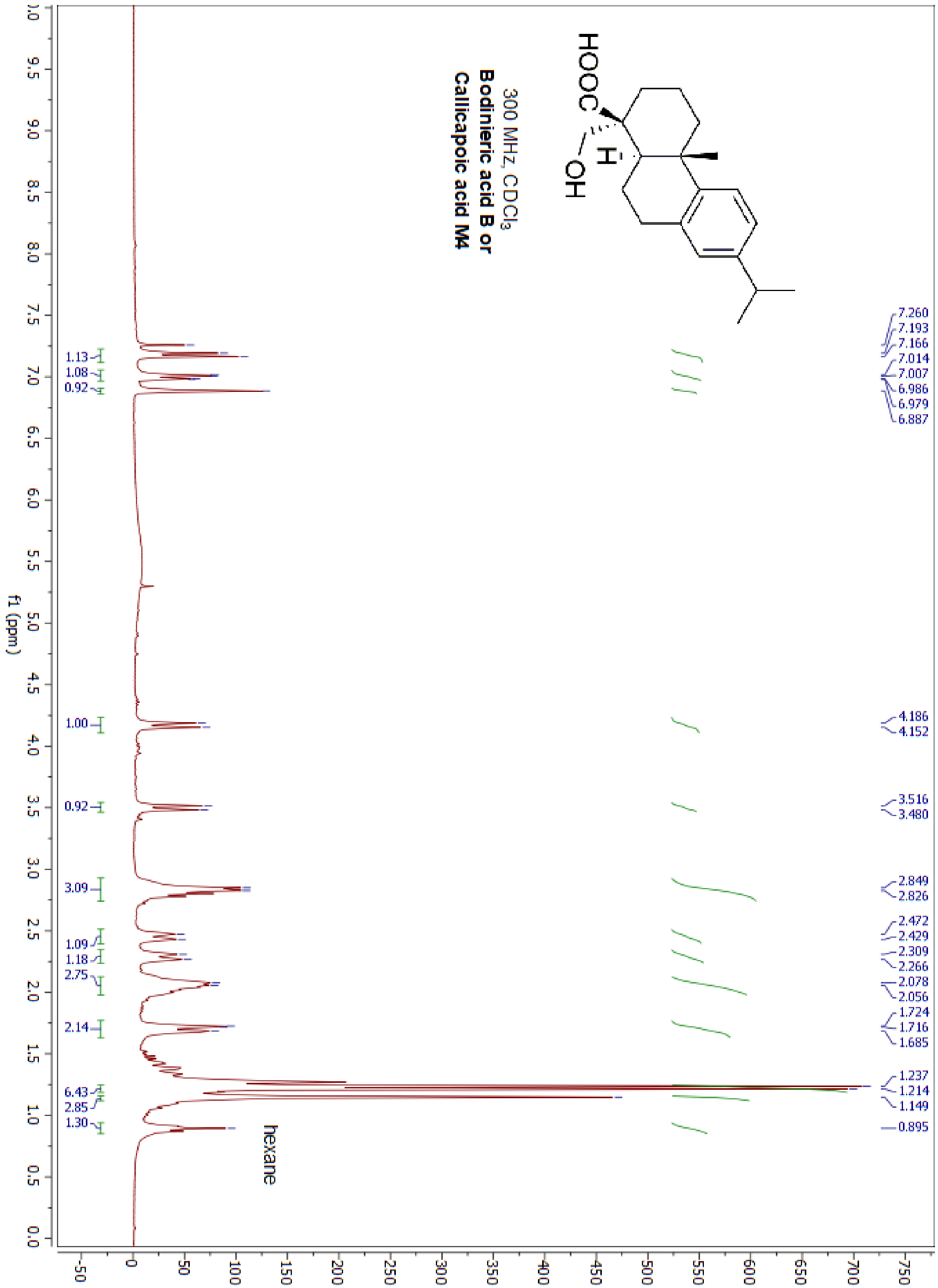

Compound 4 ($CDCl_3$)

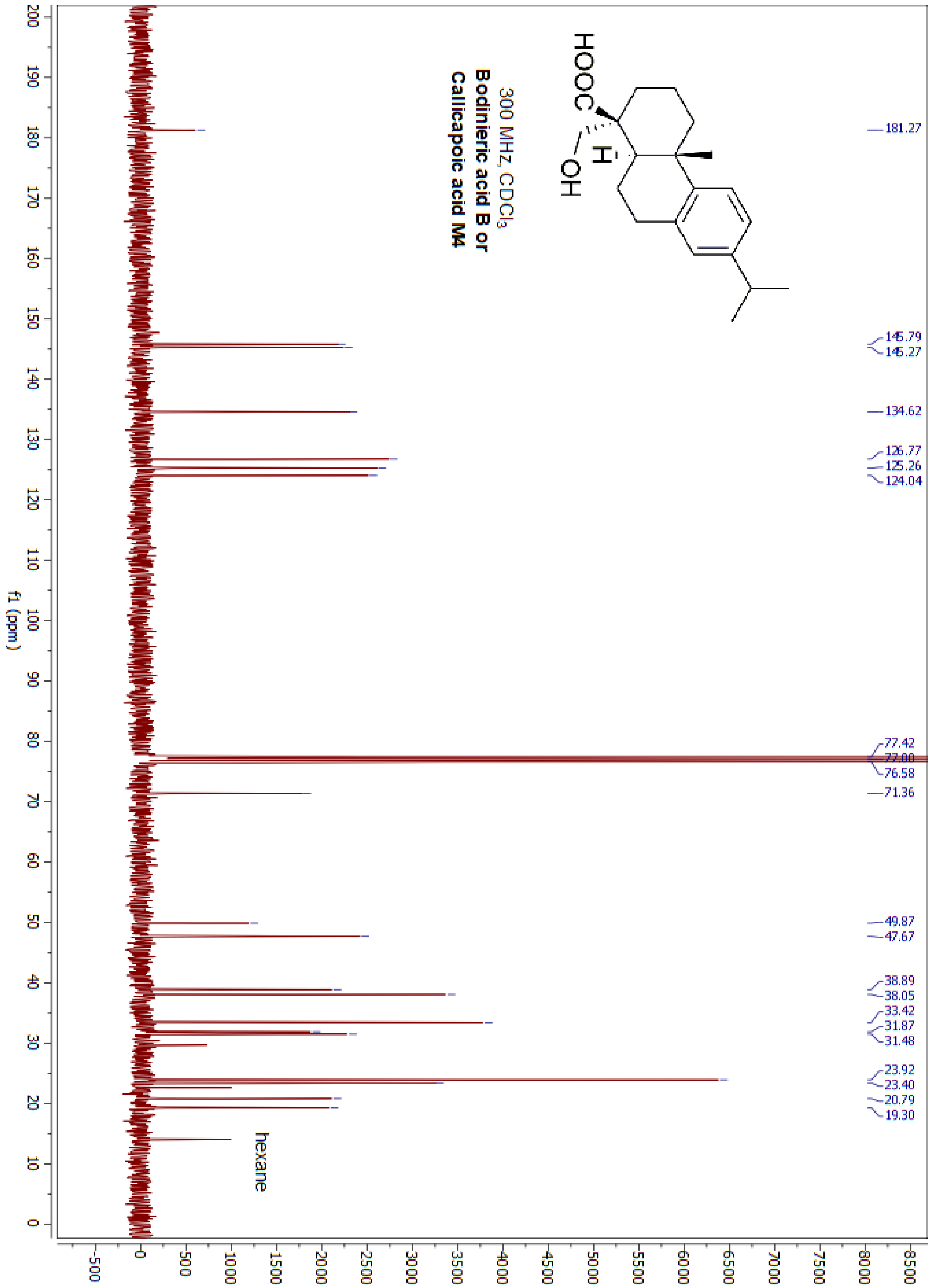

Compound 4 ($CDCl_3$)

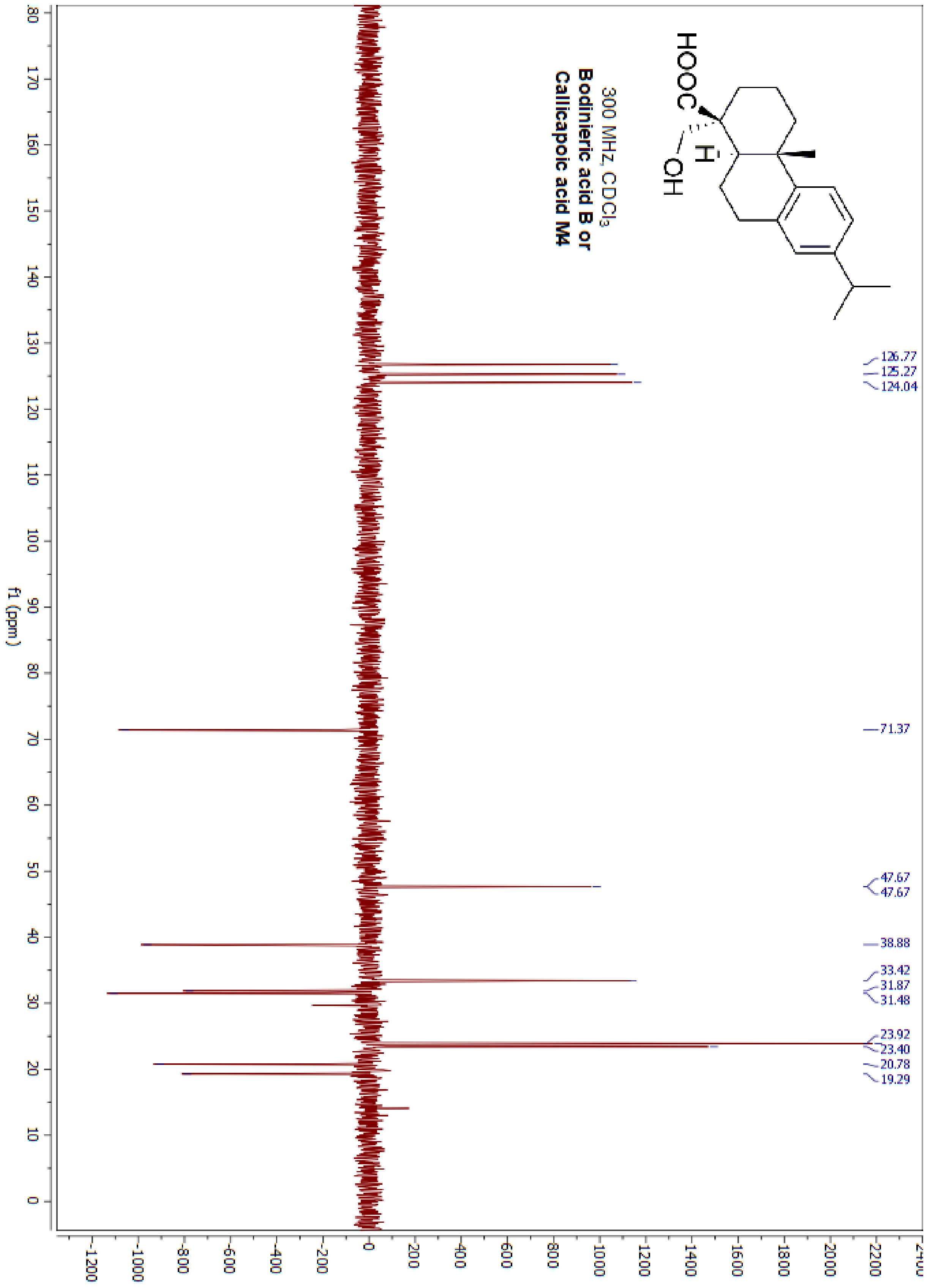

Compound 14

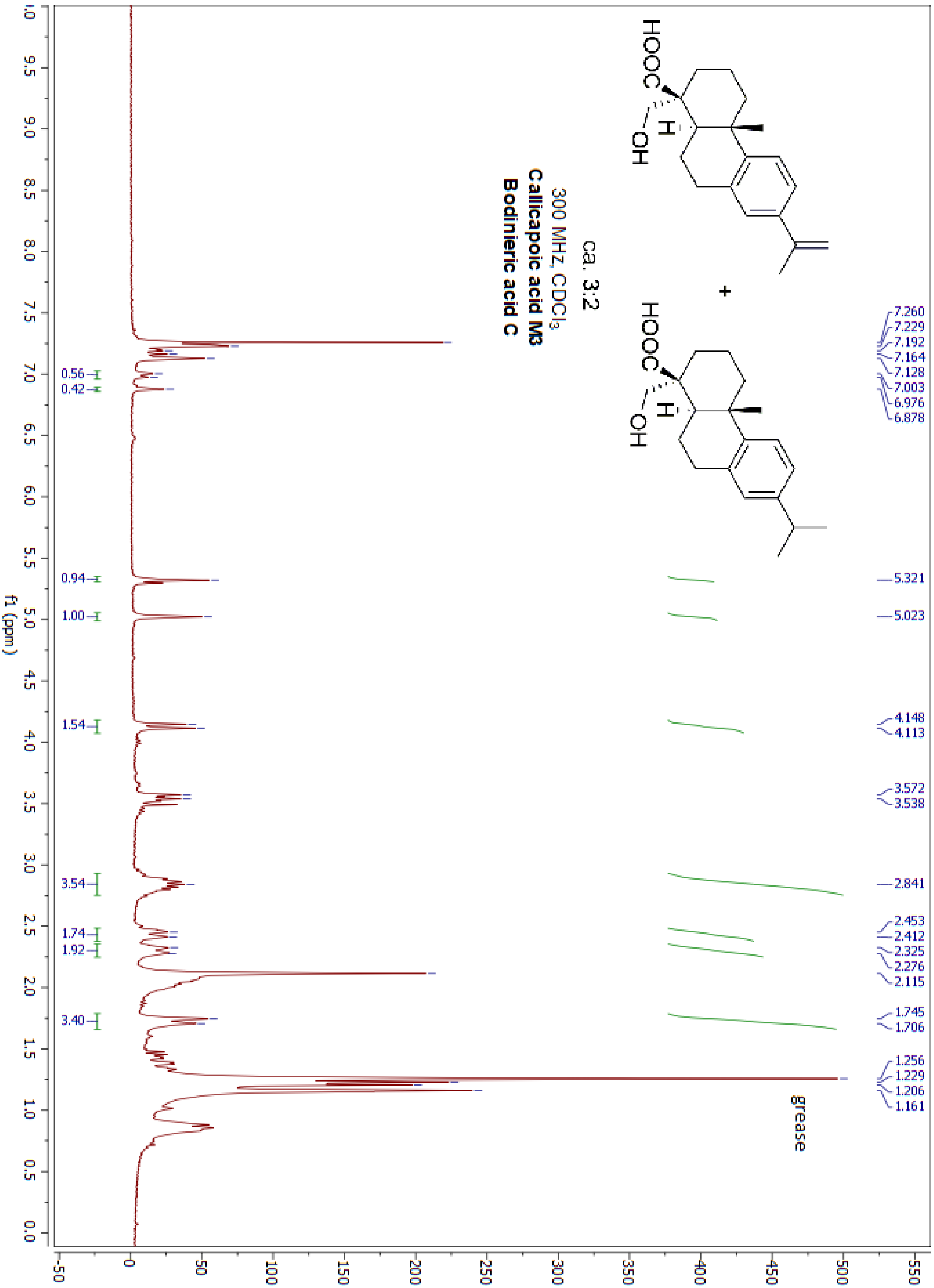
ca. 3:2
300 MHz, CDCl3
Callicapoic acid M3
Bodinieric acid C
grease
f1 (ppm)

Compound 14

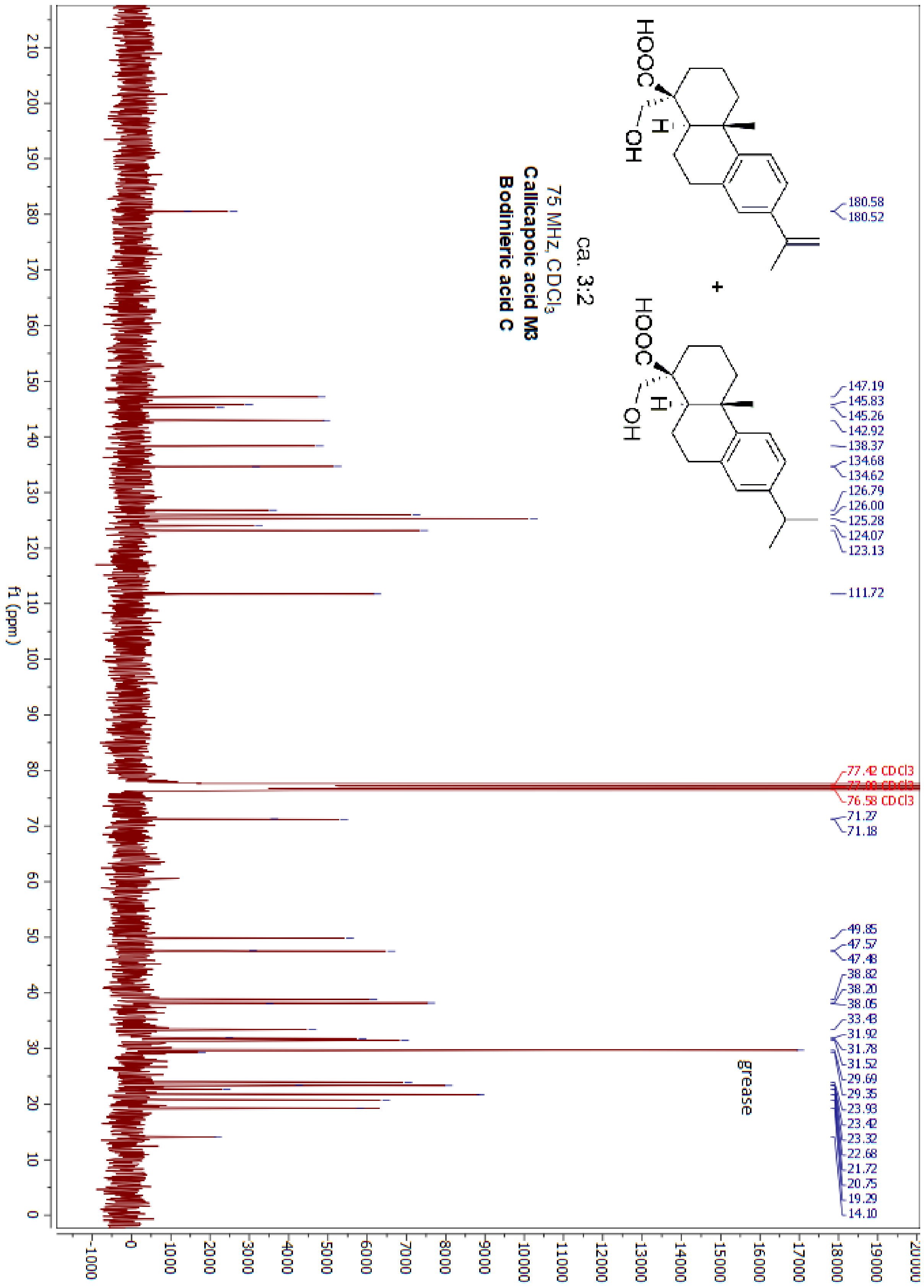

ca. 3:2
75 MHz, CDCl3
Callicapoic acid M3
Bodinieric acid C
grease
HOOC
OH
H
180.58
180.52
147.19
145.83
145.26
142.92
138.37
134.68
134.62
126.79
126.00
125.28
124.07
123.13
111.72
77.42 CDCl3
77.00 CDCl3
76.58 CDCl3
71.27
71.18
49.85
47.57
47.48
38.82
38.20
38.05
33.43
31.92
31.78
31.52
29.69
29.35
23.93
23.42
23.32
22.68
21.72
20.75
19.29
14.10
f1 (ppm)

Compound 14

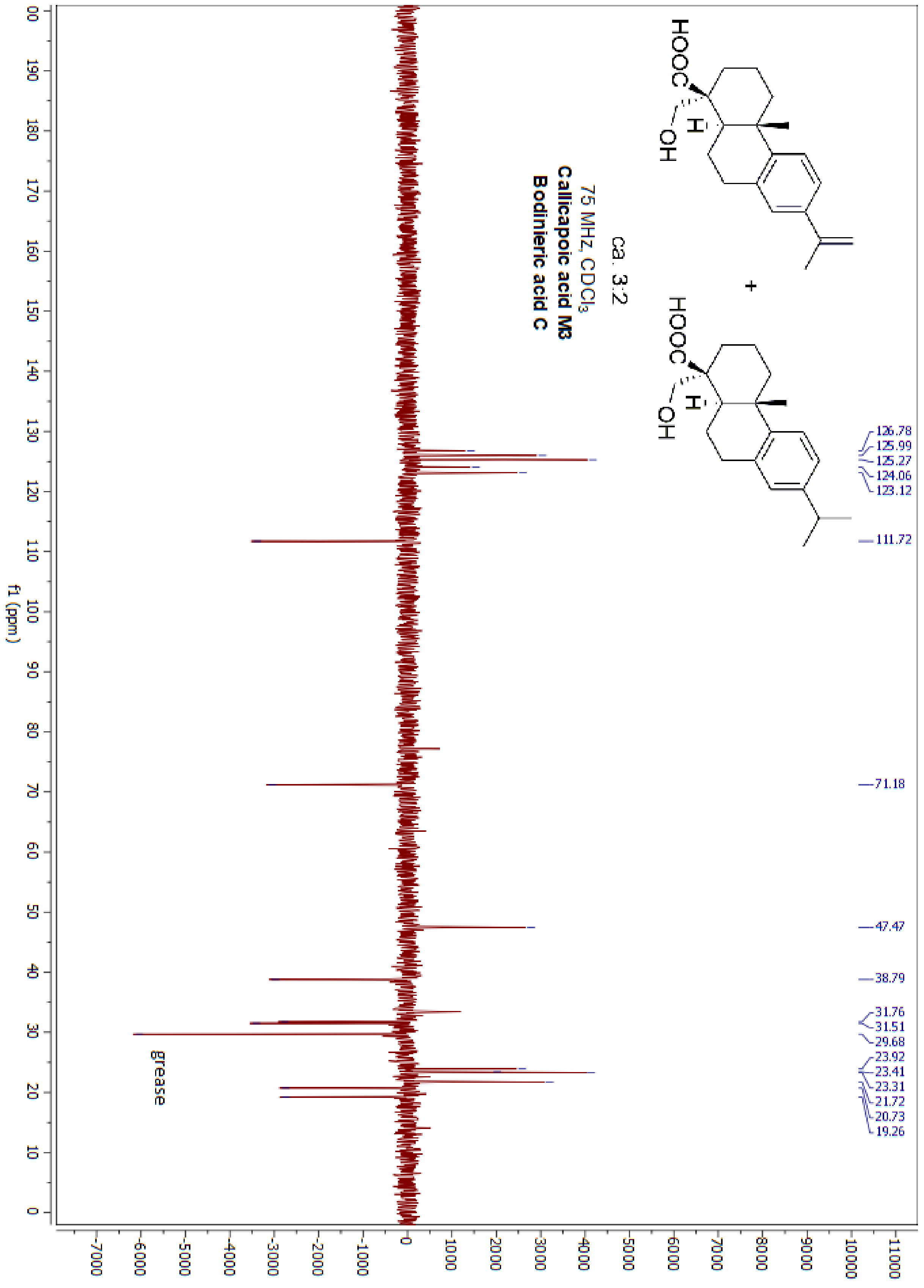

ca. 3:2
75 MHz, CDCl3
Callicapoic acid M3
Bodinieric acid C
+
HOOC
OH
H
grease
126.78
125.99
125.27
124.06
123.12
111.72
71.18
47.47
38.79
31.76
31.51
29.68
23.92
23.41
23.31
21.72
20.73
19.26
f1 (ppm)

Compound 3

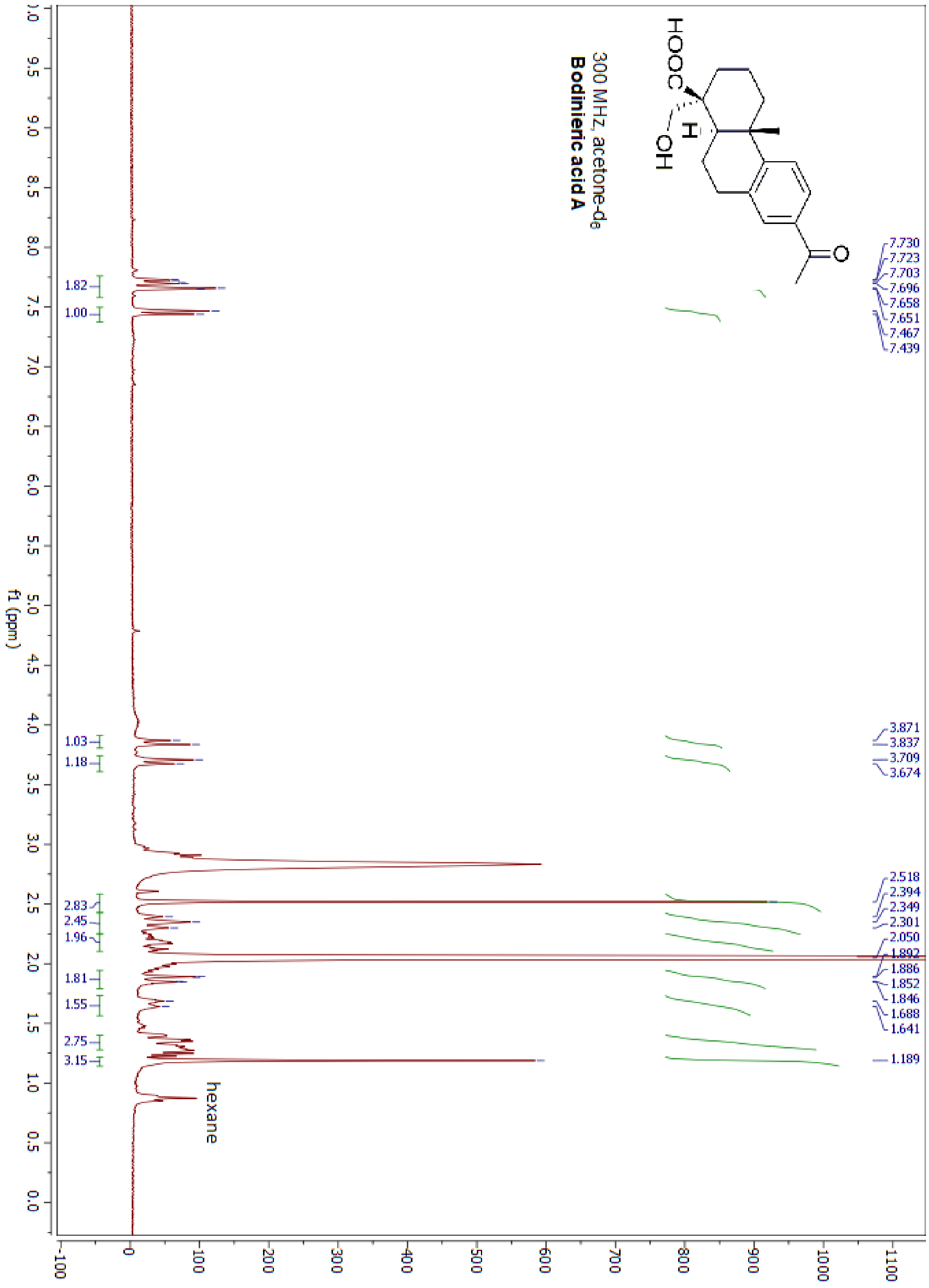

300 MHz, acetone-d6
Bodinieric acid A
HOOC
OH
H
O
hexane
7.730
7.723
7.703
7.696
7.658
7.651
7.467
7.439
3.871
3.837
3.709
3.674
2.518
2.394
2.349
2.301
2.050
1.892
1.886
1.852
1.846
1.688
1.641
1.189
1.82
1.00
1.03
1.18
2.83
2.45
1.96
1.81
1.55
2.75
3.15
f1 (ppm)

Compound 3

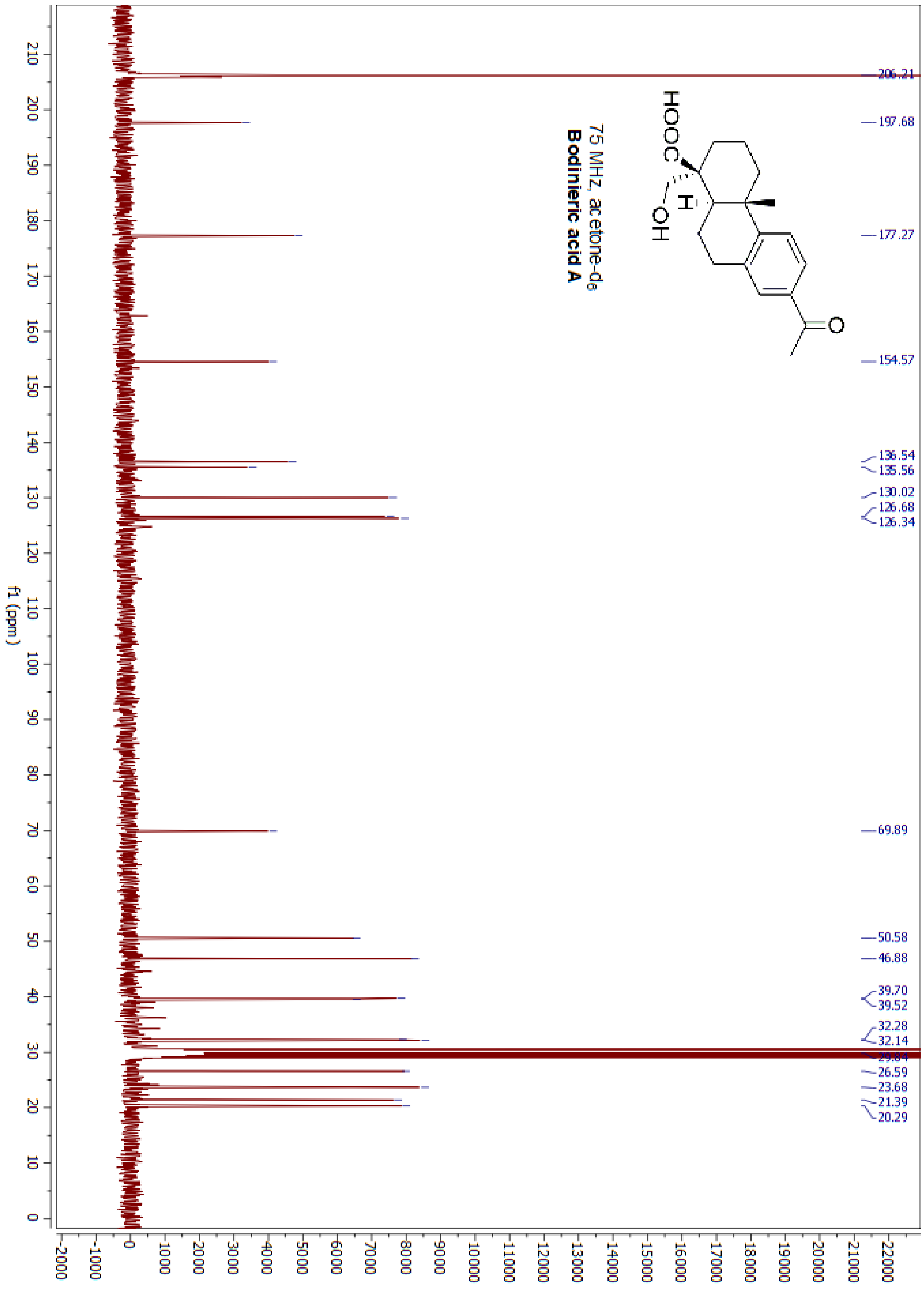
75 MHz, acetone-d6
Bodinieric acid A
HOOC
OH
H
O
206.21
197.68
177.27
154.57
136.54
135.56
130.02
126.68
126.34
69.89
50.58
46.88
39.70
39.52
32.28
32.14
29.84
26.59
23.68
21.39
20.29
f1 (ppm)

Compound 3

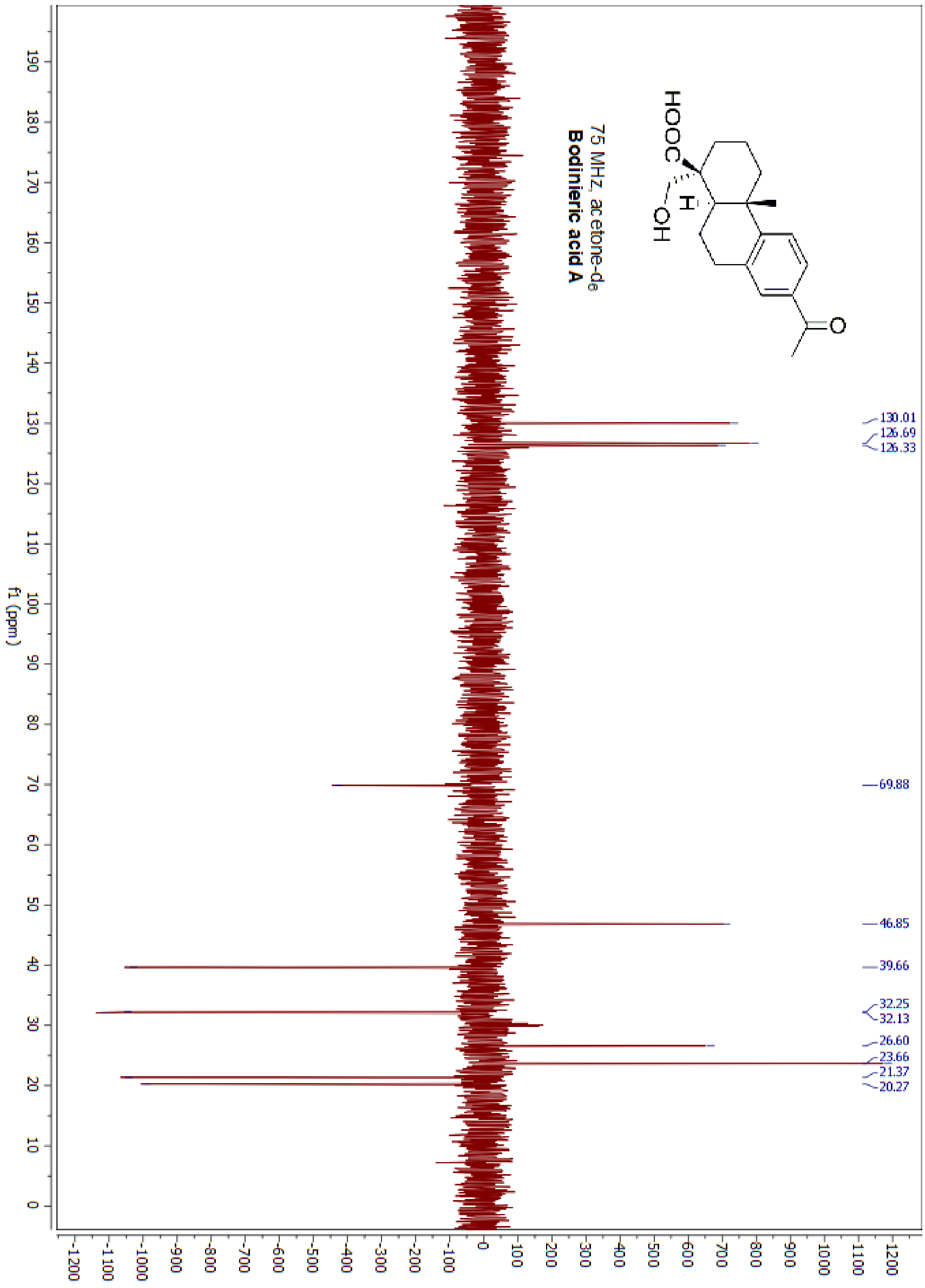
75 MHz, acetone-d6
Bodinieric acid A
HOOC
OH
H
O
130.01
126.69
126.33
69.88
46.85
39.66
32.25
32.13
26.60
23.66
21.37
20.27
f1 (ppm)

Compound 3 (COSY, acetone-d6)

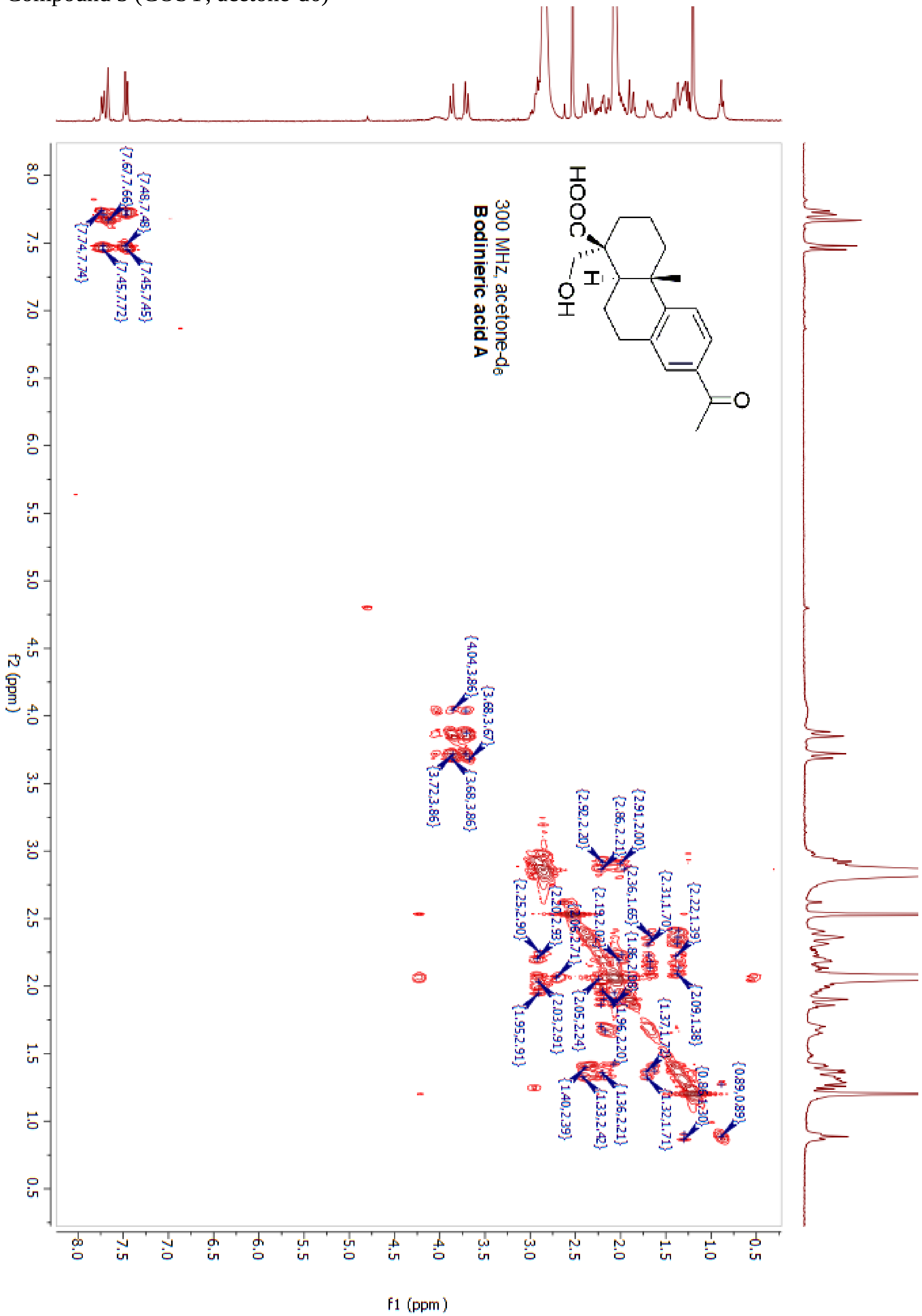

Compound 3 (COSY zoom)

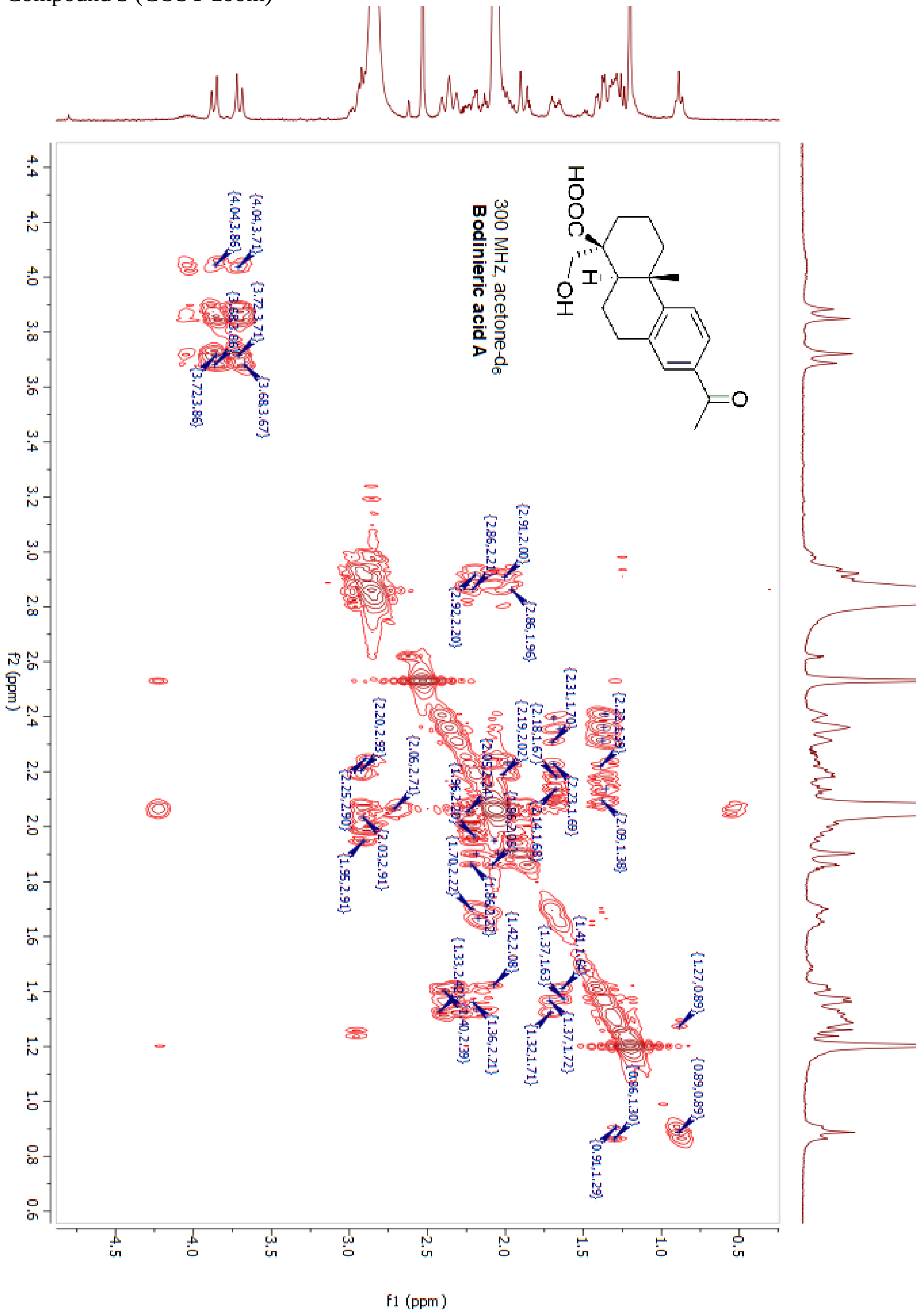

Compound 3 (HSQC, acetone-d6)

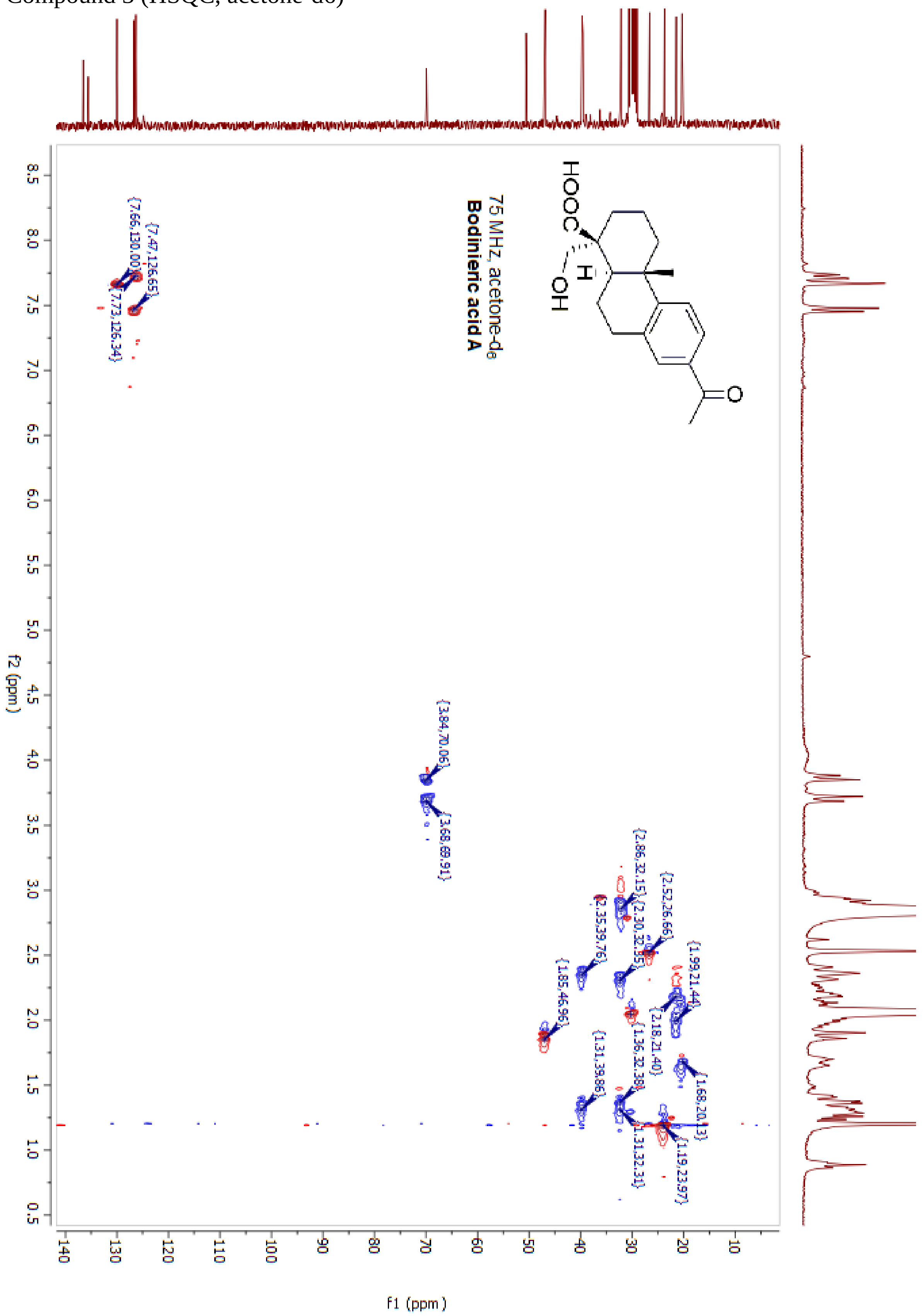

Compound 3 (HSQC zoom)

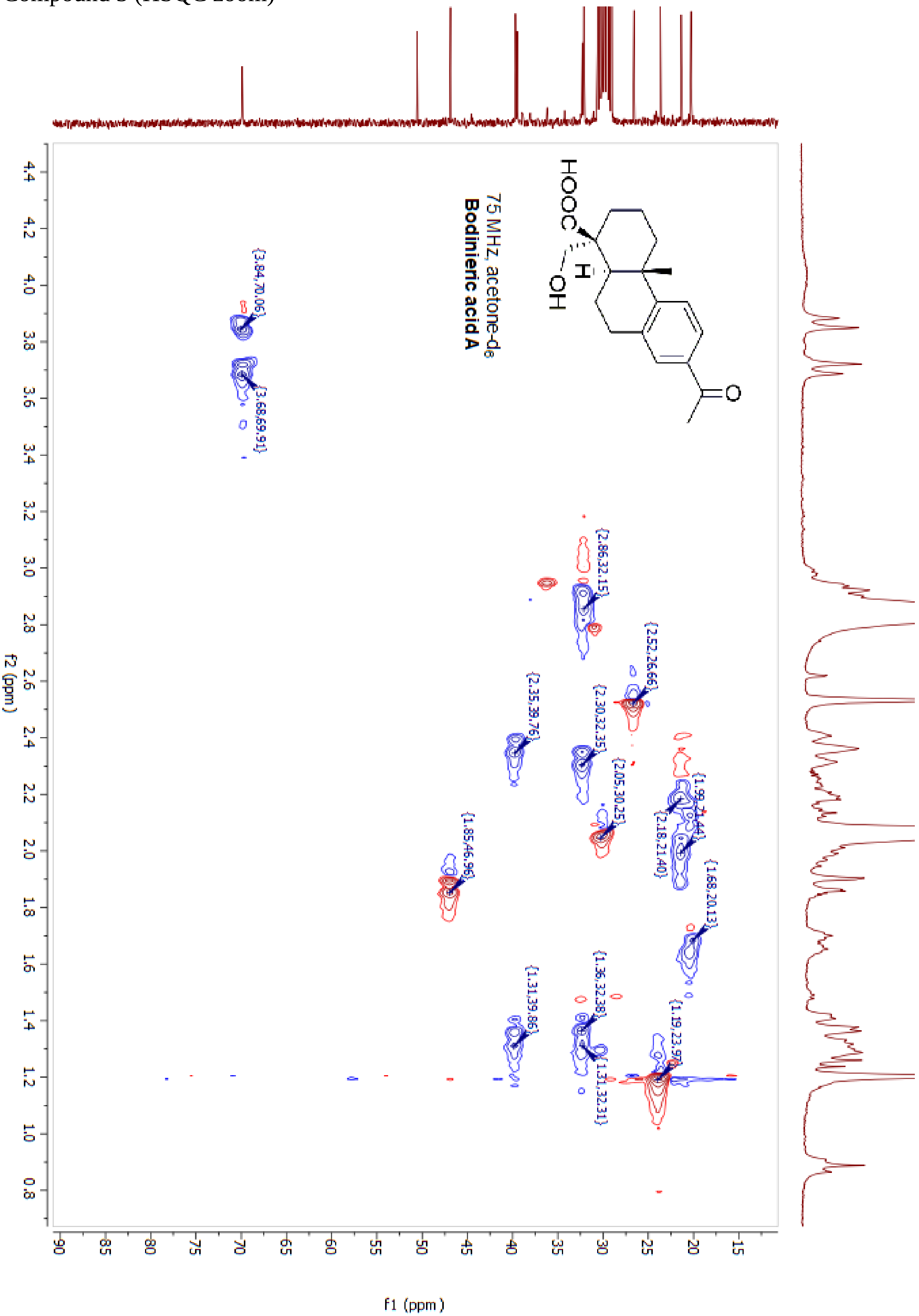

Compound 4 (COSY, acetone-d6)

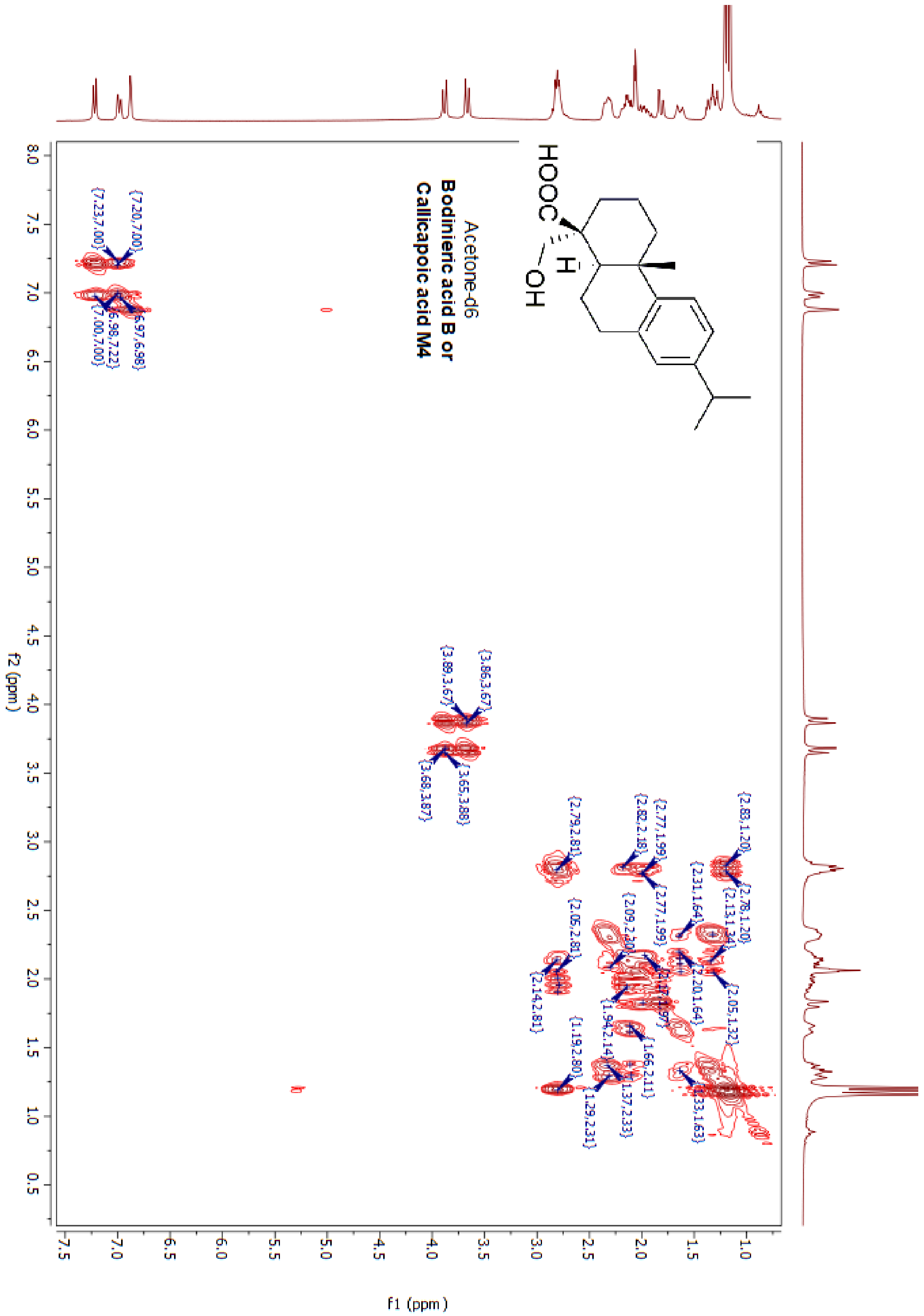

Compound 4 (HSQC, acetone-d6)

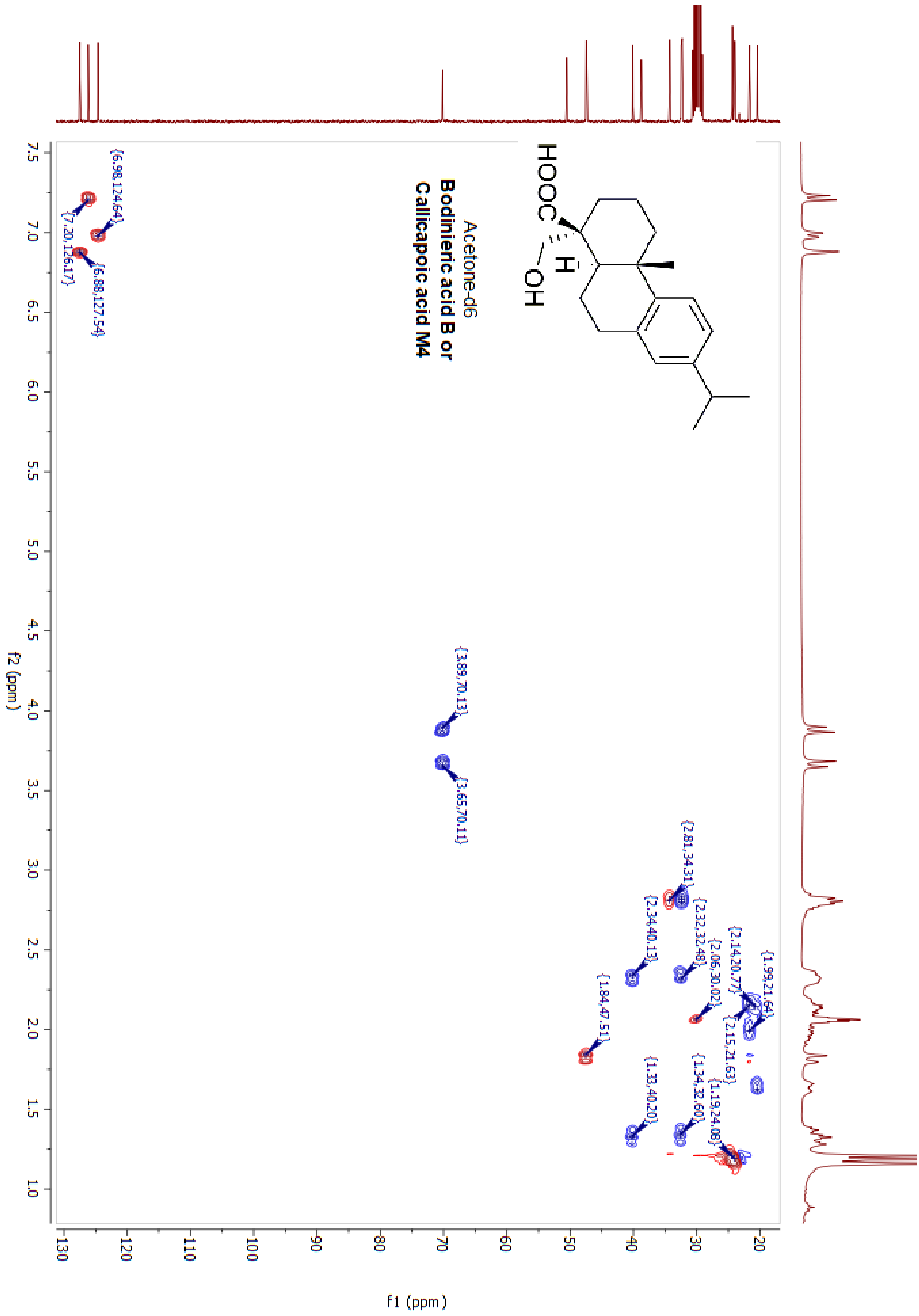

Compound 4 (COSY, $CDCl_3$)

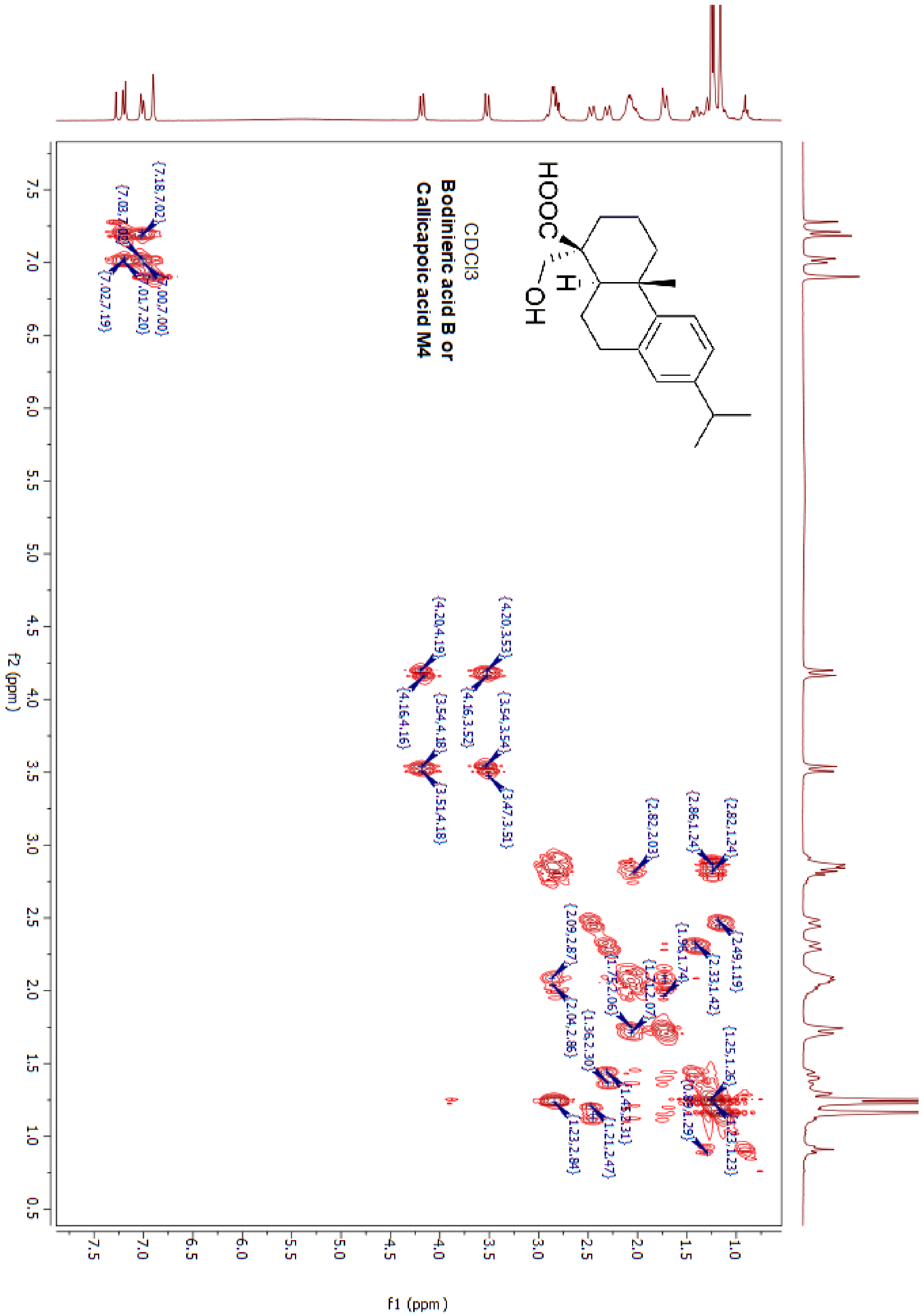

Compound 4 (HSQC, $CDCl_3$)

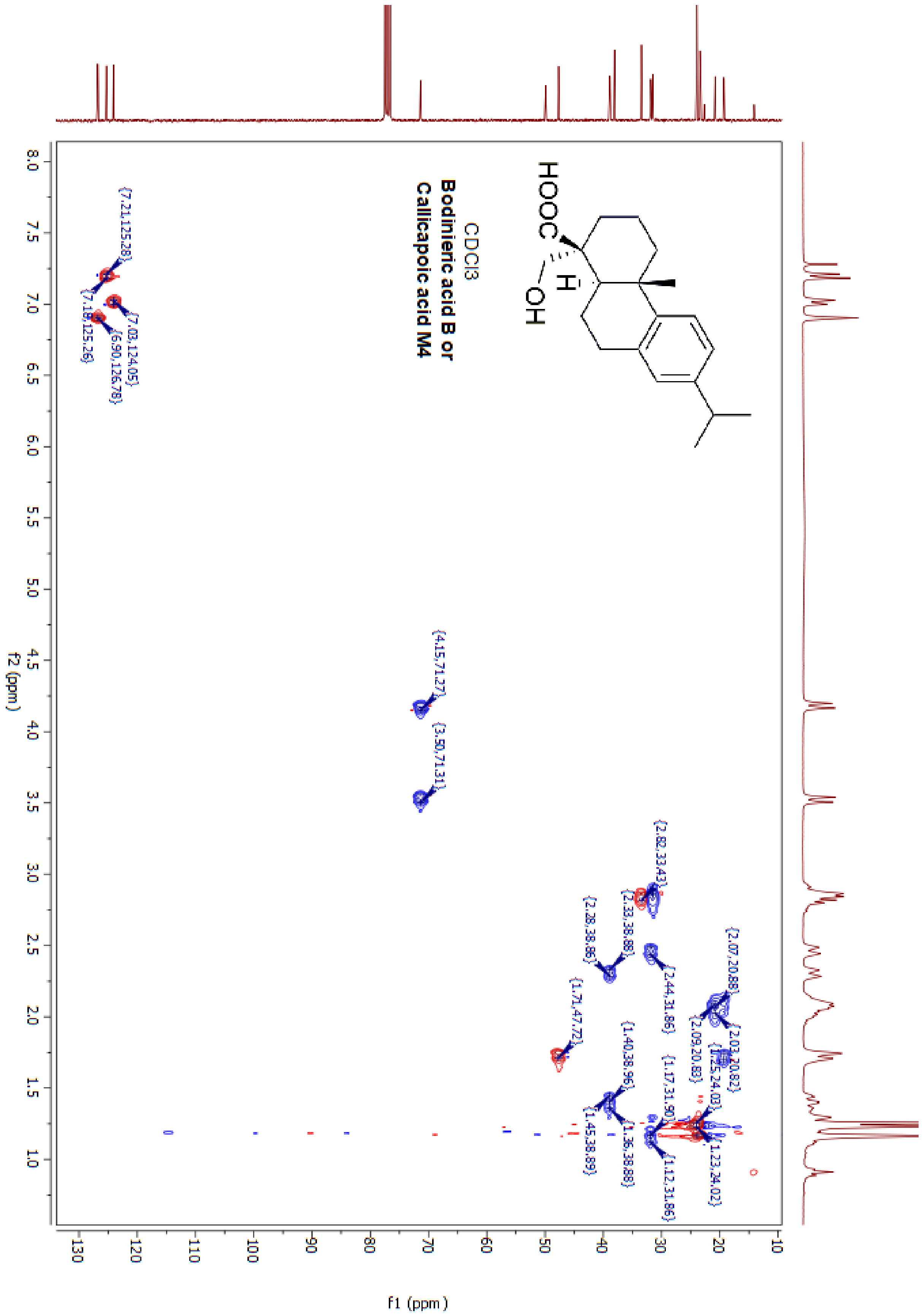

Conformational study and ^{13}C data of boninieric acid A (3).

A conformational study of bodinieric acid A (**3**) has been carried out. This molecules can display different conformations due to the rotation of the functional groups present in its structure (-CO_2H, -CH_2OH, -$COCH_3$). See figures S1 –S2 and Table S1-2.

The carboxylic group (–CO_2H) has two main conformations (A and B) which minimize its interaction with the methyl C20. The hydroxymethyl group can display three conformations (a, b and c) and finally, the acetyl group presents two conformations (x and y) with the carbonyl group in parallel with the aromatic ring.

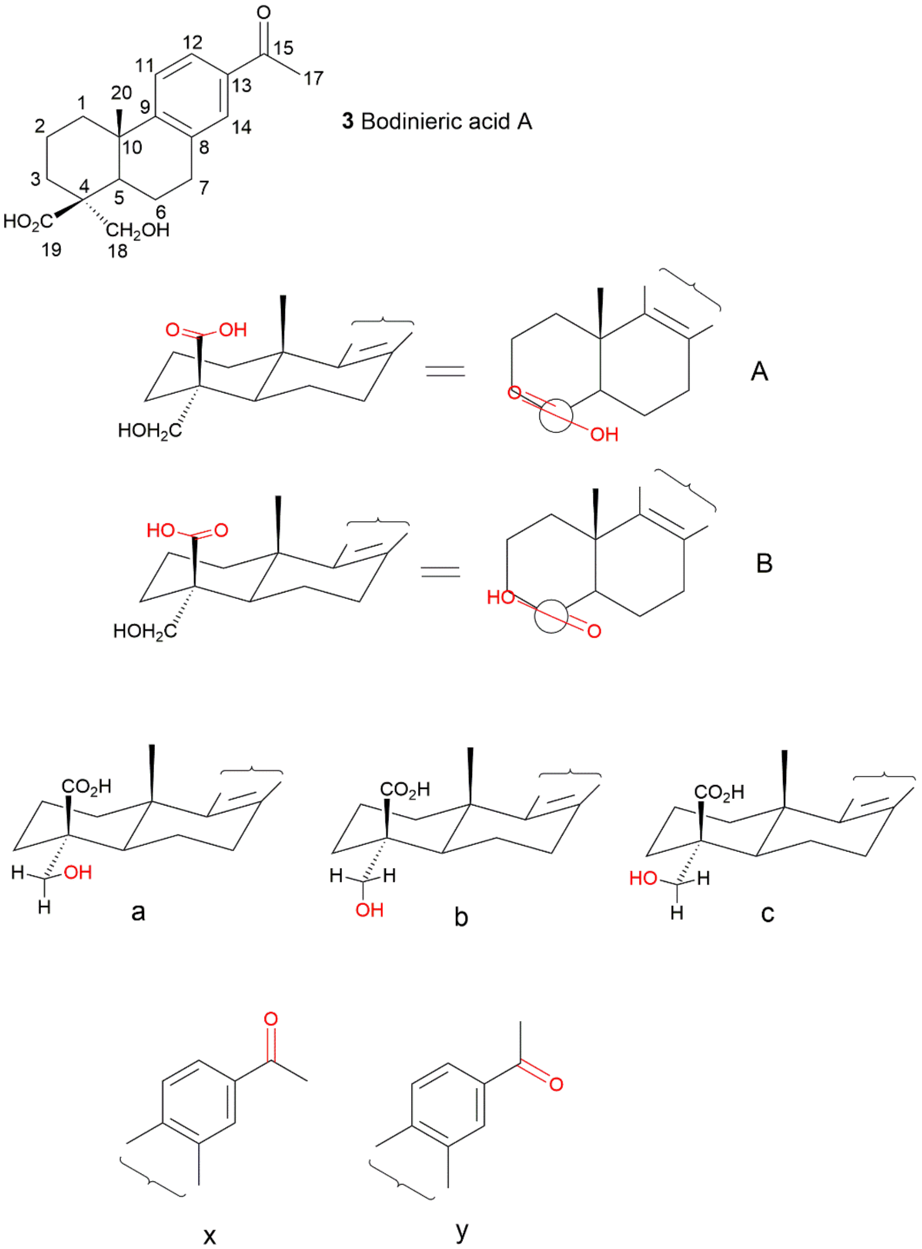

.

Figure S1. Different conformations that the groups present in bodinieric acid A (**3**) can display A/B -CO_2H group, a/b/c –CH_2OH group and x/y –$COCH_3$ group.

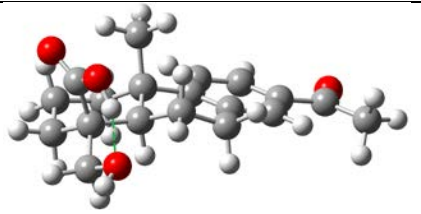	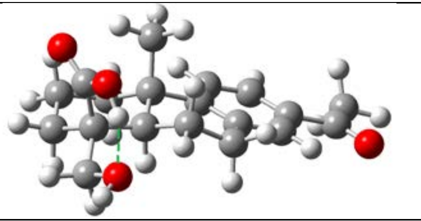	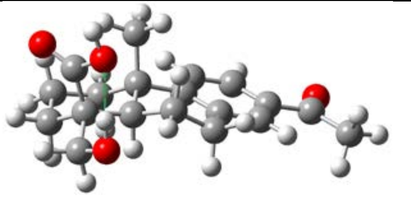
**3Aax-1**	**3Aay-1**	**3Aax-2**
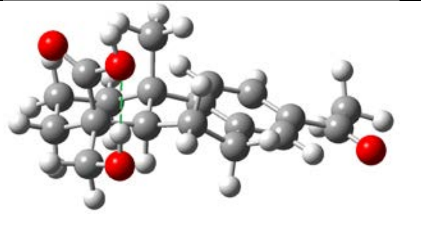	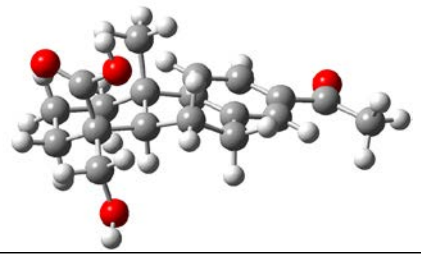	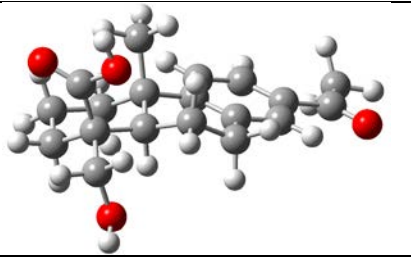
**3Aay-2**	**3Abx**	**3Aby**
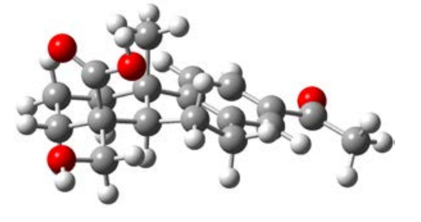	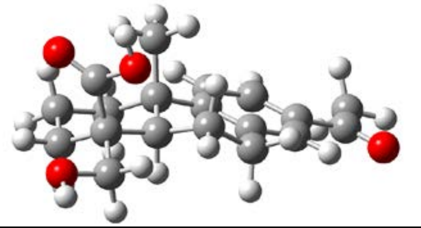	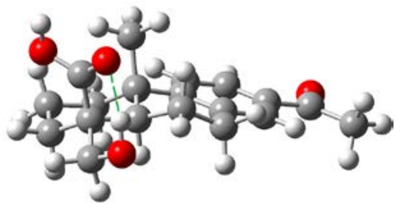
**3Acx**	**3Acy**	**3Bax**
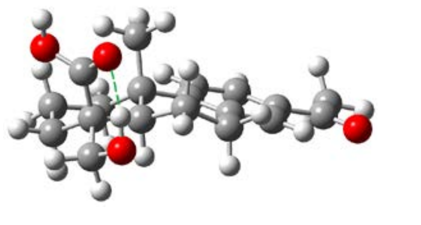	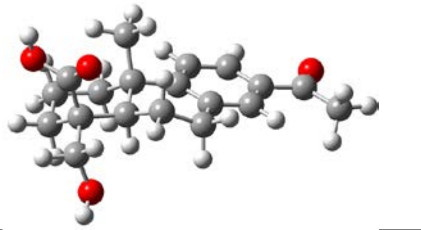	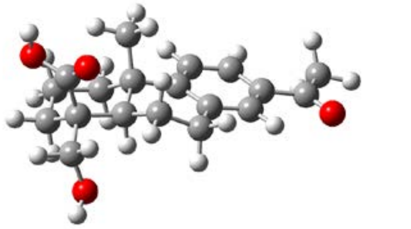
**3Bay**	**3Bbx**	**3Bby**
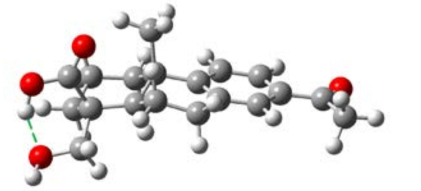	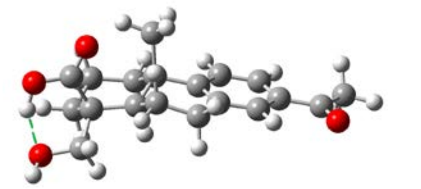	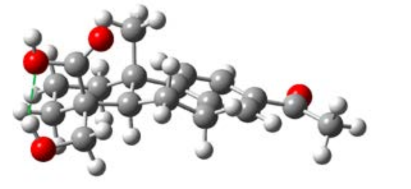
**3Bcx-1**	**3Bcy-1**	**3Bcx-2**
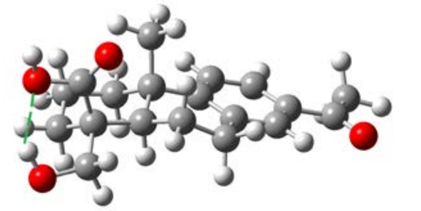		
**3Bcy-2**		

Figure S2. Geometries at B3LYP/6-31G** level of the conformations of the bodinieric acid A (**3**), in acetone (solvent used in NMR).

Table S1. B3LYP/6-31G** total energies (E, au) and ΔE (Kcal/mol) of the conformations of the bodinieric acid A (**3**), in acetone.

conformation	E	ΔE^a
**3Aax-1**	-1039.444089	0.6
**3Aay-1**	-1039.444159	0.6
**3Aax-2**	-1039.443900	0.8
**3Aay-2**	-1039.443900	0.8
**3Abx**	-1039.442167	1.8
**3Aby**	-1039.442220	1.8
**3Acx**	-1039.442684	1.5
**3Acy**	-1039.442728	1.5
**3Bax**	-1039.445108	0.0
**3Bay**	-1039.445089	0.0
**3Bbx**	-1039.441857	2.0
**3Bby**	-1039.441889	2.0
**3Bcx-1**	-1039.442202	1.8
**3Bcy-1**	-1039.442332	1.7
**3Bcx-2**	-1039.442380	1.7
**3Bcx-2**	-1039.442418	1.7

^{a} from **3Bax**

Molecular orbital calculations can be used for good estimates of ^{13}C NMR chemical shifts. Ab initio and DFT calculation of NMR shielding at very accurate levels of approximation are available in literature.[1-4] The GIAO (gauge including atomic orbital) method, implemented in the Gaussian package, is now widely used for these purposes. Good quality shielding results depend on the quality of the basis sets selected. Excellent results are obtained using B3LYP as DFT method and 6-311++G** as basis set.[3,5] The NMR calculations yield absolute shielding constants (σ) while experimental data is typically given as relative shielding constants (δ) to the tetramethylsilane (TMS).

Eqn.(1) is used to convert computed absolute shieldings (σ_{calc}), to computed relative shieldings (δ_{calc}), which can be used to compare to the experimentally measured relative shieldings (δ_{exp}).

$$\delta_{calc} = I + S\,\sigma_{calc} \qquad (1)$$

The variable I represent the shielding of the TMS, and ideally S is -1 for ^{13}C shifts. The best results are achieved when I and S are empirically determined by regressing σ_{calc} against δ_{exp} over a diverse set of organic compounds. I is the resulting intercept of the regression equation and S is the slope. We have used for I and S the values of 175.0 and -0.976, respectively.[3]

Table S2 shows the values of computed relative shieldings (δ_{calc}) and experimentally assigned relative shieldings (δ_{exp}) for the bodinieric acid A (**3**).

It should be noted that these calculations are referred only to the more stable conformation as represented in Figure S2 (**3Bax** conformation), while experimental NMR spectra are averages affected by dynamic processes such as conformational equilibrium. Remember that each of these structures, has several conformers resulting from rotation of the side chain (-CO_2H, -CH_2OH and –$COCH_3$).

Table S2. ^{13}C NMR δ shift (ppm) calculated (δ_{calc}; B3LYP/6-311++G**) and experimental ($\delta_{exp1,}$ synthesized by us; $\delta_{exp2,}$ natural product in acetone-d6) and their deviations for bodinieric acid A.

**Carbon**	δ_{calc}[a]	δ_{exp1}[b]	δ_{exp2}[c]	$\Delta_{(exp1-calc)}$	$\Delta_{(exp2-calc)}$
1	36.9	39.7	40.1	2.8	3.2
2	19.2	20.3	20.7	1.1	1.5
3	30.5	32.3	32.7	1.8	2.2
4	48.9	50.6	51.0	1.7	1.1
5	54.5	46.9	47.3	-7.6	-7.2
6	20.5	21.4	21.8	0.9	1.3
7	30.7	32.1	32.6	1.4	1.9
8	137.3	136.5[d]	135.9	-0.8	-1.4
9	156.6	154.6	155.0	-2.0	-1.6
10	41.3	39.5	39.9	-1.8	-1.4
11	124.9	126.7	127.1	1.8	2.2
12	123.6	126.3	126.7	2.7	3.1
13	132.0	135.6[d]	136.9	3.6	4.9
14	130.7	130.0	130.4	-0.7	-0.3
15	199.3	197.7	198.1	-1.6	-1.2
17	24.7	26.6	27.0	1.9	2.3
18	72.1	69.9	70.3	-2.2	-1.8
19	181.4	177.3	177.6	-4.1	-3.8
20	20.0	23.7	24.1	3.7	4.1

$\sum|\Delta_{(exp1-calc)}|/20 = 1.82$ (Mean dev.)

$\sum|\Delta_{(exp2-calc)}|/20 = 2.32$ (Mean dev.)

[a] Conformation **3Bax**
[b] Assignment made using ^{1}H, ^{13}C, DEPT, COSY(^{1}H-^{1}H) and HSQC(^{1}H-^{13}C) NMR data
[c] From ref. 6. Note a systematic displacement of about 0.4 ppm
[d] They can be interchangeable

REFERENCES

1. Barfield, M.; Fagerness, P. *J. Am. Chem. Soc.* **1997**, *119*, 8699.
2. Jaroszewska-Manaj, J.; Maciejewska, D.; Wawer, I. *Magn. Reson. Chem.* **2000**, *38*, 482.
3. Giesen, D. J.; Zumbulyadis, N. *Phys. Chem. Chem. Phys.* **2002**, *4*, 5498.
4. Azizi, S. N.; Esmaili, C. *Word Appl. Sci. J.* **2009**, *7*, 559.
5. El Moncef, A.; Zaballos, E.; Zaragoza, R. J. *Tetrahedron* **2011**, *67*, 3677.
6. Gao, J.-B.; Yang, S.-J.; Yan, Z.-R.; Zhang, X.-J.; Pu, D.-B.; Wang, L.-X.; Li, X.-L.; Zhang, R.-H.; Xiao, W.-L. *J. Nat. Prod.* **2018**, *81*, 998.

Table S3. ^{13}C NMR δ shift (ppm) experimental ($\delta_{exp-1,}$ synthesized by us; $\delta_{exp-2,}$ natural product in acetone-d6) for the bodinieric acid B.

**Carbon**	δ_{exp-1}[a]	δ_{exp-2}[b]
1	40.1	40.0
2	20.4	20.4
3	32.3	32.4
4	50.6	50.5
5	47.5	47.4
6	21.7	21.6
7	32.5	32.3
8	135.7	135.7
9	146.7	146.7
10	38.8	38.7
11	126.2	126.1
12	124.6	124.6
13	146.3	146.2
14	127.5	127.5
15	34.3	34.2
16	24.3	24.3
17	24.3	24.3
18	70.2	70.2
19	177.4	177.4
20	24.0	23.9

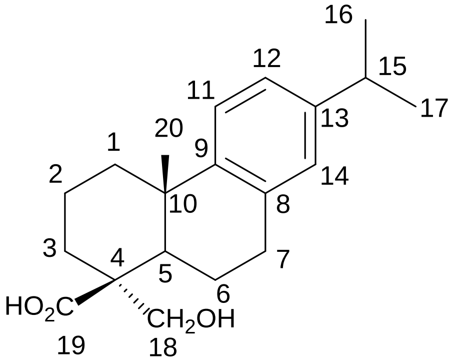

4 Bodinieric acid B

[a] Assignment made using ^{1}H, ^{13}C, DEPT, COSY(^{1}H-^{1}H) and HSQC(^{1}H-^{13}C) NMR data
[b] From ref. 6

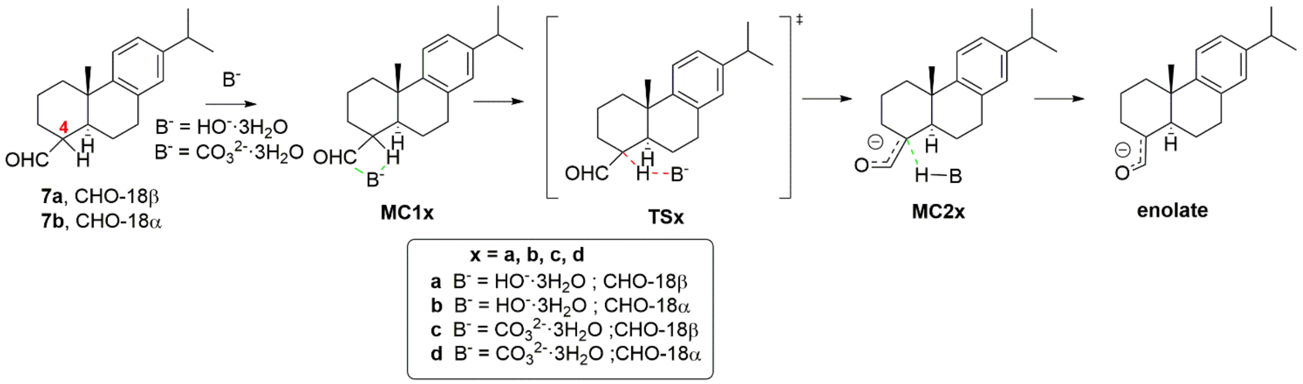

Table S4. B3LYP/6-31G** total energies (*E*, *H* and *G*, au), *S* (Cal/mol-kelvin, 298.15 K) and relative energies (Δ*G*, Kcal/mol) of species involved in the enolization of **7a** and **7b** with HO^- and CO_3^{2-}, in water.

species[a]	*E*	*H*	*S*	*G*	Δ*G*[b]
$HO^-\cdot 3H_2O$	-305.243381	-305.149574	94.776	-305.194605	
$CO_3^{2-}\cdot 3H_2O$	-493.358827	-493.258217	102.559	-493.306947	
**7a**	-814.962819	-814.534078	139.394	-814.600309	
**7b**	-814.963267	-814.534304	140.921	-814.601260	
**MC1a**	-1120.218366	-1119.692724	192.438	-1119.784159	0.0
**TSa**	-1120.205347	-1119.684161	189.599	-1119.774245	6.2
**MC2a**	-1120.211987	-1119.686169	194.646	-1119.778652	3.5
**MC1b**	-1120.221580	-1119.695703	194.542	-1119.788136	0.0
**TSb**	-1120.199806	-1119.678659	188.450	-1119.768198	12.5
**MC2b**	-1120.208470	-1119.682726	196.543	-1119.776110	7.5
**MC1c**	-1308.329607	-1307.798500	209.269	-1307.897930	0.0
**TSc**	-1308.318887	-1307.792536	198.591	-1307.886894	6.9
**MC2c**	-1308.330814	-1307.799800	206.124	-1307.897736	0.1
**MC1d**	-1308.333744	-1307.801650	201.024	-1307.897163	0.0
**TSd**	-1308.313233	-1307.787316	203.895	-1307.884193	8.1
**MC2d**	-1308.326260	-1307.797225	197.798	-1307.891206	3.7

[a] Structures fully optimized.
[b]from **MC1a**, **MC1b**, **MC1c** or **MC1d**

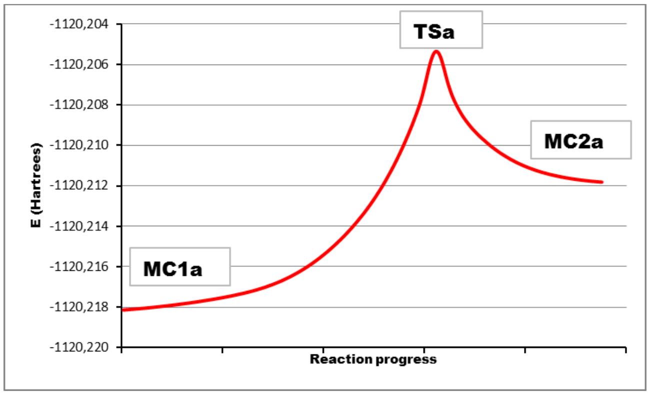

Figure S3. Energy profile (IRC, *E*) corresponding to the transition structure **TSa** in **water**

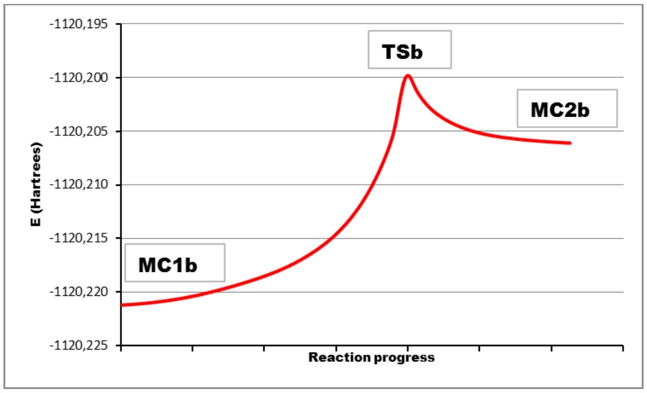

Figure S4. Energy profile (IRC, *E*) corresponding to the transition structure **TSb** in **water**

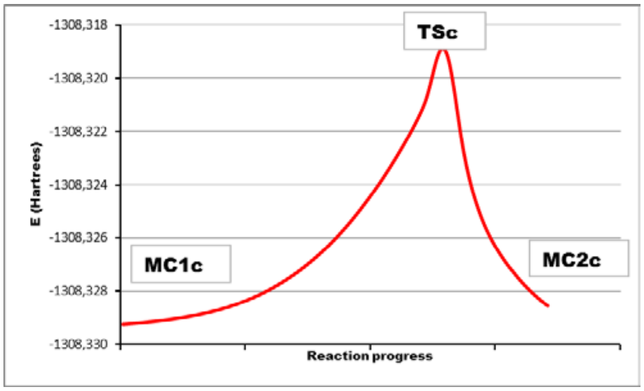

Figure S5. Energy profile (IRC, *E*) corresponding to the transition structure **TSc** in **water**

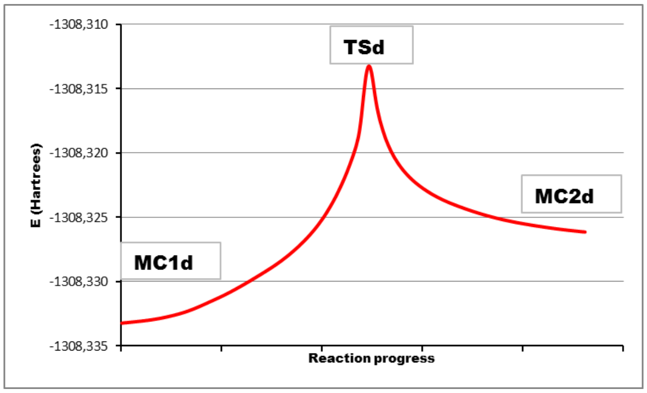

Figure S6. Energy profile (IRC, *E*) corresponding to the transition structure **TSd** in **water**

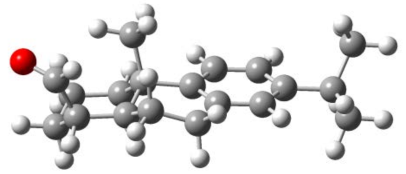	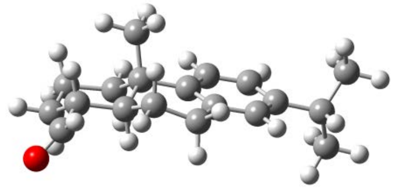	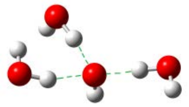
**7a**	**7b**	$HO^{-}\cdot 3H_2O$
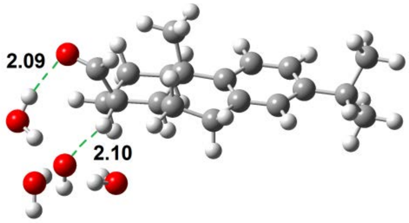	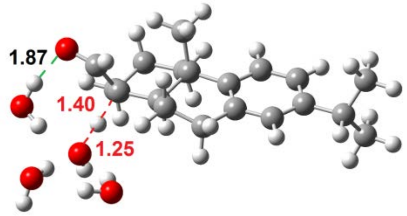	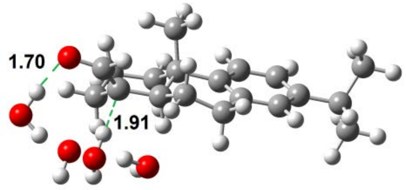
**MC1a**	**TSa**	**MC2a**
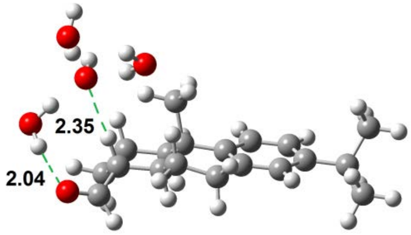	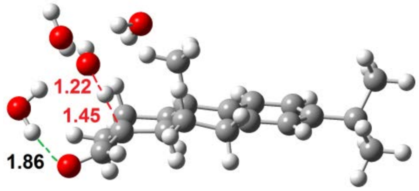	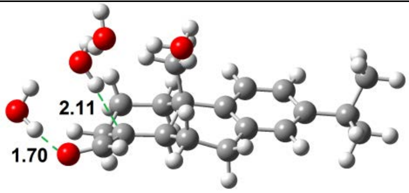
**MC1b**	**TSb**	**MC2b**

Figure S7. Geometries at B3LYP/6-31G** level of species involved in the enolization of **7a** and **7b** with HO^{-}, in water.

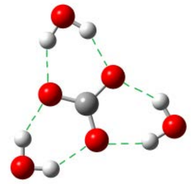		
$CO_3^{2-}\cdot 3H_2O$		
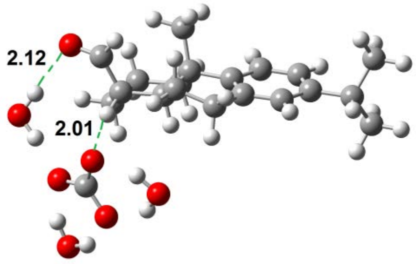	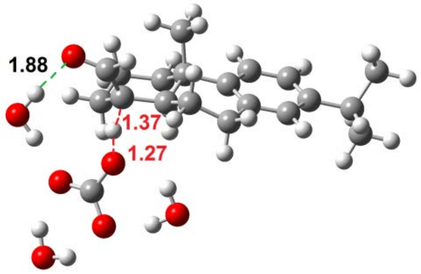	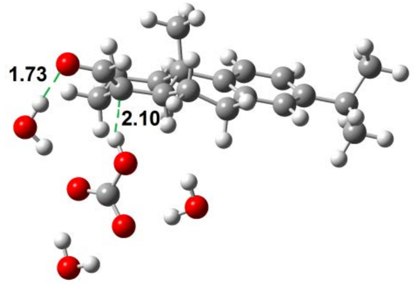
**MC1c**	**TSc**	**MC2c**
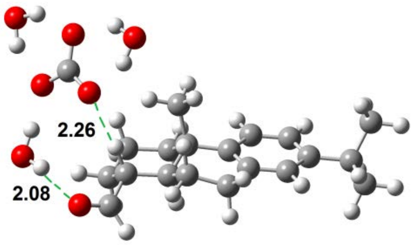	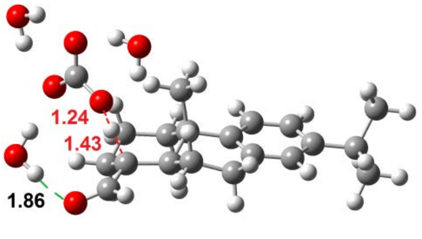	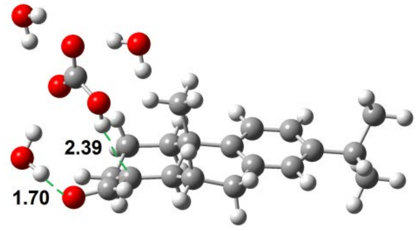
**MC1d**	**TSd**	**MC2d**

Figure S8. Geometries at B3LYP/6-31G** level of species involved in the enolization of **7a** and **7b** with CO_3^{2-}, in water.

XYZ matrices B3LYP/6-31G**

$HO^{-}\cdot 3H_2O$

-1 1

O	-0.40286100	-0.29448500	0.37749700
H	-1.95012200	-0.07009200	-0.00897000
H	-0.42353700	-0.28235800	1.34484600
O	-2.93148500	0.05210900	-0.23012700
H	-3.27659900	-0.84773300	-0.17668100
H	1.04450200	-1.13450800	0.04280700
O	2.00292400	-1.30235200	-0.18395700
H	2.25773600	-0.37790600	-0.33964500
H	0.58354900	0.89271100	0.05253500
O	1.33771700	1.55348500	-0.16555200
H	1.71410600	1.74982700	0.70221300

$CO_3^{2-}\cdot 3H_2O$

-2 1

C	0.00185300	-0.00595500	0.05179200
O	-1.25868700	0.32218200	0.09591900
O	0.91299300	0.92392100	-0.00021200
O	0.35018300	-1.25985100	0.06085200
H	-1.34174500	2.18238200	0.00135700
H	-1.23186300	-2.25945900	-0.04595300
H	2.55184000	0.07023500	-0.03672900
O	-2.21245600	-2.20358700	-0.08132700
H	-2.20353900	-1.21733000	-0.02060200
O	-0.81934800	3.01429400	-0.05367700
H	0.03913300	2.52878000	-0.05502900
O	3.02618900	-0.79246100	-0.04014700
H	2.18405600	-1.30486500	-0.00506200

7a

0 1

C	4.11792600	0.86400100	0.67300200
O	5.04807900	0.22279200	1.12909500
H	3.78284500	1.79009300	1.18510400
C	3.39486500	0.54619800	-0.61836100
H	3.74242600	1.35531400	-1.28480300
C	1.84818800	0.69818300	-0.56685100
H	1.53947000	0.59607900	-1.61824000
C	3.79736300	-0.80573900	-1.22680200
H	4.88303200	-0.92948000	-1.16993500
H	3.52838100	-0.79368700	-2.29038000
C	1.12412900	-0.46028800	0.18632600
C	3.08860700	-1.98002600	-0.53855900
H	3.31726200	-2.91020300	-1.07183300
H	3.48923900	-2.10484200	0.47384000
C	1.56335300	-1.79331900	-0.48516200
H	1.16218400	-1.81378300	-1.50741500
H	1.12164100	-2.64627200	0.04044200
C	1.34545900	2.07444800	-0.11323800
H	1.94272600	2.87028700	-0.57354000

H	1.44679200	2.19595600	0.97081900
C	-0.12205300	2.22987700	-0.52075700
H	-0.16887700	2.42605100	-1.60181700
H	-0.56581500	3.10900000	-0.03935900
C	-0.39972800	-0.25291100	0.07092600
C	-0.96658800	1.00423500	-0.21663900
C	-1.27783500	-1.32224100	0.32251700
C	-2.36372400	1.13859400	-0.25347000
C	-2.65992100	-1.17153200	0.27882700
H	-0.87415100	-2.30037200	0.56433500
C	-3.23425100	0.07479200	-0.01363700
H	-2.78130000	2.11919700	-0.47702300
H	-3.29336900	-2.03188500	0.47819400
C	-4.74359400	0.27121600	-0.06393500
H	-4.92202300	1.32817900	-0.30053600
C	-5.39293700	-0.56810200	-1.18040600
H	-6.46860900	-0.36870200	-1.23658700
H	-4.95277500	-0.33802900	-2.15590600
H	-5.26236500	-1.64043300	-0.99777400
C	-5.40560800	-0.01653300	1.29673600
H	-5.27314700	-1.06395200	1.58938800
H	-4.97671700	0.60875900	2.08622700
H	-6.48177300	0.18265500	1.25166500
C	1.46405400	-0.50466300	1.69930700
H	1.16282100	0.41449300	2.20917800
H	0.92328100	-1.32942400	2.17403500
H	2.52796700	-0.66446600	1.89021000

7b

0 1

C	-4.30175100	-1.38544700	-0.18119100
O	-5.07232100	-1.45519000	-1.12101100
H	-4.21990800	-2.23745800	0.52660800
C	-3.44317800	-0.17980100	0.13908100
H	-3.67385800	0.05019200	1.19159800
C	-1.91897600	-0.49866300	0.03776300
H	-1.72547600	-0.67757400	-1.03162800
C	-3.80820100	1.01306000	-0.75648800
H	-4.87205400	1.24944600	-0.64610000
H	-3.66680600	0.71303500	-1.80240400
C	-2.94784300	2.24182400	-0.45241100
H	-3.16620000	3.03431800	-1.17780200
H	-3.21773400	2.64798600	0.53012400
C	-1.04301300	0.73858700	0.43305200
C	-1.44745800	1.91919900	-0.49397900
H	-0.88543300	2.81999200	-0.22798700
H	-1.16164800	1.66487800	-1.52336300
C	-1.45363300	-1.75771600	0.78581300
H	-1.48637100	-1.59169400	1.86863500
H	-2.11019300	-2.60923800	0.58700600
C	-0.03698000	-2.12808300	0.33698500
H	-0.09752600	-2.61712400	-0.64607500
H	0.39303100	-2.87665700	1.01292700
C	0.44661500	0.38627700	0.24688300
C	1.40877700	1.40707500	0.14332800

C	0.90482600	-0.94440200	0.22647400
C	2.76394600	1.13458200	-0.01275000
H	1.09578300	2.44531000	0.18392600
C	2.27591800	-1.20173000	0.06870100
C	3.22640800	-0.18909100	-0.06414900
H	3.46269600	1.96222300	-0.09904600
H	2.60632400	-2.23924500	0.04526500
C	4.69522700	-0.52201300	-0.28938700
H	4.79715600	-1.60979300	-0.18260300
C	5.61989600	0.13330300	0.75173900
H	5.33788400	-0.15188900	1.77023800
H	6.65836100	-0.17415000	0.58765300
H	5.58550700	1.22629900	0.68677200
C	5.13279700	-0.15604000	-1.72125700
H	6.17331900	-0.45074100	-1.89593200
H	4.50693200	-0.65789700	-2.46606400
H	5.05664300	0.92362000	-1.89206900
C	-1.23797300	1.15393800	1.91547300
H	-0.92052700	0.36481200	2.60181400
H	-0.62866200	2.03690200	2.13255600
H	-2.27509700	1.40681400	2.15196400

MC1a

-1 1

C	2.74006100	-1.05287500	1.27929400
O	3.62197400	-1.87655500	1.48816500
H	2.36423800	-0.44624800	2.12807600
C	2.17679700	-0.71090300	-0.07072100
H	2.85694400	0.11341800	-0.38384900
C	0.73494400	-0.14800200	-0.06313900
H	0.57456900	0.19334800	-1.09784100
C	2.32020100	-1.83517400	-1.11181200
H	3.33271300	-2.24426200	-1.07544900
H	2.20242600	-1.38321700	-2.10565200
O	4.56203500	1.22752900	-0.89520500
H	5.29532100	-0.28640300	-0.70995300
H	4.64505100	1.32571600	-1.85490400
O	5.69674500	-1.19853200	-0.60351400
H	5.14793000	-1.58807300	0.09261800
H	3.51831400	2.56202400	-0.53888800
O	3.18850200	3.43226900	-0.18292300
H	3.99879500	3.69748300	0.28360400
H	5.55753600	2.30048100	-0.24169900
O	6.05759200	3.06230300	0.21443800
H	6.25489200	3.66269800	-0.51618000
C	-0.36032600	-1.23271600	0.18179800
C	1.25730000	-2.92431000	-0.92966400
H	1.32427700	-3.65188300	-1.74763100
H	1.45837600	-3.48681400	-0.00958600
C	-0.16668000	-2.34169200	-0.89339500
H	-0.40212600	-1.91340000	-1.87715500
H	-0.87955500	-3.15705800	-0.73076300
C	0.52122200	1.09053700	0.81899200
H	1.35761200	1.78994800	0.70508900
H	0.47658500	0.81887900	1.88017500

C	-0.78474900	1.77572300	0.40717400
H	-0.61569200	2.31805900	-0.53480500
H	-1.07123300	2.53863400	1.14074800
C	-1.75169100	-0.57823200	0.06319800
C	-1.94080900	0.81078200	0.20520000
C	-2.89411100	-1.37512700	-0.13175200
C	-3.23956900	1.34011300	0.13394100
C	-4.17331100	-0.83293400	-0.20183100
H	-2.78549500	-2.45091600	-0.22834400
C	-4.37133800	0.54986800	-0.07038500
H	-3.36394400	2.41633700	0.24570400
C	-0.27364300	-1.86887100	1.59380900
H	-0.40493400	-1.12469200	2.38389700
H	-1.06743100	-2.61306800	1.71406100
H	0.67553600	-2.38350600	1.76910900
C	-5.75938400	1.17224300	-0.14080000
H	-5.63336400	2.25419000	-0.00449800
C	-6.67310900	0.66681200	0.99144200
H	-7.64663400	1.16771300	0.95444500
H	-6.85015600	-0.41108100	0.90724200
H	-6.22947300	0.85790100	1.97377400
C	-6.41600700	0.95315900	-1.51677500
H	-7.38742600	1.45703800	-1.56512500
H	-5.78806500	1.34664300	-2.32248700
H	-6.58344900	-0.11190100	-1.71157500
H	-5.02171300	-1.49449900	-0.35729000

TSa

1 imaginary frequencies = -1128.8300

-1 1

C	2.94213000	-0.93920800	1.27361400
O	3.97122600	-1.62967100	1.48212700
H	2.55278100	-0.34004800	2.12465400
C	2.27575900	-0.72771200	0.02896300
H	3.07370000	0.31804500	-0.43739100
C	0.84722800	-0.16653400	0.02555200
H	0.69922000	0.24530900	-0.98807500
C	2.47680900	-1.77078200	-1.07211900
H	3.48707600	-2.18467600	-1.01433200
H	2.39845400	-1.27831900	-2.05407800
O	3.79633100	1.18621700	-0.96562100
H	5.22051200	-0.11076600	-0.77950200
H	3.72063000	1.09581200	-1.92621200
O	5.77874600	-0.88166000	-0.55601000
H	5.25148300	-1.28782400	0.16088300
H	2.80774300	2.78323900	-0.65306700
O	2.66155200	3.68245400	-0.29300500
H	3.54903100	3.83880400	0.07592300
H	4.96109900	2.31978400	-0.31121800
O	5.45356200	3.07681500	0.09411300
H	5.80615800	3.55179200	-0.67001700
C	-0.25053300	-1.27784100	0.16469000
C	1.41655400	-2.88091200	-1.00302900
H	1.51273300	-3.55818900	-1.86229600
H	1.59082900	-3.49341000	-0.10951800

C	-0.01149700	-2.30911900	-0.97533700
H	-0.21551600	-1.81458000	-1.93547500
H	-0.72523600	-3.13680800	-0.89591900
C	0.57645000	1.00202600	0.98331600
H	1.39641800	1.72649800	0.93908700
H	0.51173700	0.65488500	2.02093100
C	-0.72948000	1.69466800	0.58475500
H	-0.54326800	2.30417500	-0.31211300
H	-1.04788700	2.40205000	1.36036800
C	-1.65005100	-0.64129400	0.05373000
C	-1.86577900	0.73202200	0.28597100
C	-2.77631000	-1.43861600	-0.21943600
C	-3.17099900	1.24611800	0.22321400
C	-4.06312000	-0.91212000	-0.27816900
H	-2.64839100	-2.50379300	-0.38605100
C	-4.28617400	0.45533800	-0.05743000
H	-3.31494000	2.31025000	0.40615900
C	-0.18208700	-1.99948100	1.53469700
H	-0.40640900	-1.31759300	2.36001900
H	-0.92060300	-2.80755700	1.57160500
H	0.80167100	-2.43840700	1.72046600
H	-4.89761300	-1.57436100	-0.49458500
C	-5.68284000	1.06052100	-0.10836400
H	-5.57330800	2.13633100	0.08081600
C	-6.59506700	0.48826900	0.99303900
H	-6.15945900	0.63888100	1.98594900
H	-7.57615600	0.97539200	0.97438200
H	-6.75443400	-0.58703300	0.85632500
C	-6.33006400	0.89897700	-1.49643600
H	-7.30735200	1.39302200	-1.52617100
H	-5.70341400	1.33744300	-2.27954100
H	-6.48418700	-0.15749800	-1.74217000

MC2a

-1 1

C	3.04010200	-0.64912400	1.20197900
O	4.17627800	-1.21392800	1.42437100
H	2.69892500	0.08383100	1.96345000
C	2.19567200	-0.79592600	0.11256700
H	3.21782900	0.54591800	-0.78366600
C	0.80228100	-0.19107200	0.04157200
H	0.65633300	0.14608000	-1.00235500
C	2.41645300	-1.89123000	-0.91251800
H	3.41791600	-2.31497600	-0.79671200
H	2.36714300	-1.48143900	-1.93715000
O	3.75521000	1.18708500	-1.34428900
H	5.28956200	-0.30429200	-1.16510500
H	3.54032700	0.99320900	-2.26635600
O	5.79363600	-0.99778000	-0.71435700
H	5.23145300	-1.15231400	0.09236200
H	2.92351600	2.95149200	-0.71506700
O	2.98628700	3.69942800	-0.09759900
H	3.88815800	3.55402500	0.24468400
H	5.18654400	1.99756700	-0.40195100
O	5.64441200	2.63955600	0.17511400

H	6.11607100	3.20988400	-0.44693800
C	-0.31609700	-1.27605700	0.25415000
C	1.33327300	-2.98039300	-0.79685700
H	1.42402800	-3.70598700	-1.61705100
H	1.48580000	-3.54464400	0.13180800
C	-0.08466400	-2.38264900	-0.81536700
H	-0.27357900	-1.94595400	-1.80656800
H	-0.81360900	-3.19168400	-0.69136700
C	0.53673000	1.04611100	0.90942900
H	1.36291700	1.76009900	0.81968200
H	0.47223300	0.77332900	1.96915000
C	-0.76432800	1.71897300	0.46223300
H	-0.57635400	2.25965300	-0.47756500
H	-1.07460600	2.48456800	1.18426600
C	-1.70891600	-0.63833800	0.09906000
C	-1.91151000	0.74955100	0.23546500
C	-2.84330000	-1.44208100	-0.11554500
C	-3.21196600	1.27050800	0.13994400
C	-4.12546600	-0.90888900	-0.20849200
H	-2.72532200	-2.51743500	-0.20744300
C	-4.33529100	0.47254800	-0.08250200
H	-3.34564500	2.34615000	0.24862600
C	-5.72617200	1.08593000	-0.17419100
H	-5.60865200	2.16920000	-0.03998100
C	-6.36399200	0.85843800	-1.55763200
H	-7.33786500	1.35609800	-1.62057200
H	-5.72806600	1.25287000	-2.35661800
H	-6.52241500	-0.20830600	-1.75096700
C	-6.65200700	0.57921700	0.94765400
H	-6.82109900	-0.50011100	0.86513900
H	-6.22246900	0.77640200	1.93507800
H	-7.62820700	1.07378100	0.89640700
C	-0.24439100	-1.89796000	1.67087800
H	-0.51551000	-1.16992400	2.44133400
H	-0.94393800	-2.73689200	1.75594300
H	0.76058400	-2.26400500	1.89500300
H	-4.96705300	-1.57642500	-0.37637300

MC1b

-1 1

C	-2.83287100	-1.59786200	1.81795000
O	-4.01920000	-1.47513100	2.08081400
H	-2.15975600	-2.07243500	2.56580100
C	-2.18991500	-1.22968300	0.50428400
H	-2.75333800	-0.39350200	0.07030600
C	-0.70342100	-0.86765500	0.70287500
H	-0.22730200	-1.74416100	1.17524500
C	-2.35794500	-2.47185800	-0.41602100
H	-3.42570800	-2.60253300	-0.61664100
H	-2.00387400	-3.36882900	0.11232000
O	-4.00888500	1.33199300	-0.90308400
H	-4.95952500	-0.01401100	-0.66499200
H	-3.87818800	1.35069200	-1.86172500
O	-5.48673800	-0.84163600	-0.45013000
H	-5.15021200	-1.06180700	0.43262300

H	-2.80158200	2.42730300	-0.33635300
O	-2.36769800	3.24392100	0.03673500
H	-3.17410500	3.75482000	0.21773100
H	-4.85587400	2.65191700	-0.57805400
O	-5.26014700	3.55220600	-0.31976600
H	-5.15421100	4.07691100	-1.12393200
C	-1.57522100	-2.31835100	-1.72473200
H	-1.62387300	-3.25736500	-2.28896500
H	-2.05843500	-1.55848400	-2.34984900
C	0.05462200	-0.64657200	-0.64269200
C	-0.10648700	-1.94073800	-1.48572500
H	0.38830900	-1.82893400	-2.45584500
H	0.40137400	-2.76547900	-0.96729900
C	-0.49702300	0.31896700	1.65424000
H	-0.85339900	1.24491500	1.18957700
H	-1.09609900	0.19027000	2.56492400
C	0.98072400	0.42208400	2.04553500
H	1.19137700	-0.32270900	2.82680300
H	1.19148100	1.39601000	2.50344000
C	1.53529700	-0.34923800	-0.34125800
C	2.51825000	-0.53434400	-1.32966400
C	1.94627200	0.18513400	0.89589400
C	3.85762800	-0.22856400	-1.10930100
H	2.23438200	-0.92440500	-2.30186000
C	3.30207200	0.48707500	1.10084000
C	4.27928100	0.28976500	0.12414500
H	4.57673200	-0.39466900	-1.90721100
H	3.59927600	0.89471600	2.06616400
C	5.73892900	0.62949100	0.39497800
H	5.79356500	1.01681300	1.42067900
C	6.25549700	1.73503300	-0.54491000
H	5.64151300	2.63814600	-0.46966300
H	7.28772500	2.00308900	-0.29455200
H	6.24171100	1.40612200	-1.58986600
C	6.63923800	-0.61832100	0.32289000
H	7.67445800	-0.36285500	0.57401700
H	6.30083000	-1.39158900	1.01992600
H	6.63783500	-1.05067600	-0.68382600
C	-0.50211400	0.56135500	-1.44408200
H	-0.29951300	1.50810000	-0.93816800
H	-0.01804400	0.60321900	-2.42601700
H	-1.58306900	0.50626500	-1.59275300

TSb

1 imaginary frequencies = -1178.9978

-1 1

C	-3.07403400	-0.60768500	1.78315800
O	-4.15371700	-1.18554800	2.06404800
H	-2.77214400	0.22691700	2.44895700
C	-2.24004500	-0.84907300	0.64582900
H	-2.97116800	0.03842400	-0.23400400
C	-0.74840200	-0.45298000	0.77141300
H	-0.28320900	-1.16312600	1.48185100
C	-2.45498800	-2.23416700	0.02030600
H	-3.51864800	-2.37336300	-0.19397300

H	-2.18294200	-3.01627600	0.75064300
O	-3.68229800	0.71953400	-0.95799400
H	-5.19134500	-0.43178200	-0.57483000
H	-3.62217100	0.39336500	-1.86711600
O	-5.78687300	-1.11928700	-0.21675500
H	-5.33347400	-1.31101900	0.62862200
H	-2.58652900	2.32857400	-1.05344000
O	-2.39993400	3.28009000	-0.93035700
H	-3.26526600	3.56018700	-0.58270600
H	-4.76719200	2.06242200	-0.56996700
O	-5.20021700	2.92154300	-0.34374200
H	-5.55255900	3.22692700	-1.19022400
C	-1.62833900	-2.45364700	-1.24910300
H	-1.71413800	-3.49783200	-1.57640800
H	-2.03408200	-1.84663200	-2.06836200
C	0.07561400	-0.65434200	-0.55106000
C	-0.14841000	-2.11195600	-1.03495200
H	0.39601500	-2.29228000	-1.96814000
H	0.27469100	-2.79862300	-0.28798500
C	-0.48516100	0.95278400	1.33876900
H	-0.78330100	1.71709800	0.61277300
H	-1.09298400	1.13881800	2.22805000
C	0.98725300	1.10291900	1.73890800
H	1.13368700	0.63377200	2.72309400
H	1.24006600	2.16117800	1.88089500
C	1.56669200	-0.38495900	-0.26555700
C	2.56976000	-0.92521300	-1.09074600
C	1.97416600	0.47436800	0.77291400
C	3.91988300	-0.64764500	-0.89771000
H	2.29464100	-1.57775400	-1.91269800
C	3.34014400	0.74326100	0.95452200
C	4.33485600	0.19934100	0.14062600
H	4.65250200	-1.09367900	-1.56555100
H	3.63109600	1.40818300	1.76691300
C	5.80411500	0.52625100	0.37309600
H	5.85138900	1.18680800	1.24869200
C	6.41411500	1.29221800	-0.81622500
H	5.85675500	2.21145000	-1.02302600
H	7.45470300	1.56453000	-0.60830900
H	6.40479700	0.68286400	-1.72673300
C	6.63023600	-0.73194600	0.69825900
H	7.66966200	-0.46501000	0.91820500
H	6.22325900	-1.25942000	1.56684200
H	6.63806400	-1.43089100	-0.14527200
C	-0.33436400	0.32400700	-1.68237700
H	-0.10925500	1.36142400	-1.42272400
H	0.22347300	0.08784300	-2.59523600
H	-1.39863000	0.26704000	-1.91086300

MC2b

-1 1

C	-3.28756000	-0.28458600	1.59649900
O	-4.51338700	-0.63770500	1.78995800
H	-2.96478300	0.63184000	2.13150400
C	-2.32357600	-0.87204200	0.79244100

H	-3.47560500	0.19759700	-0.61084100
C	-0.83296900	-0.56434000	0.91922900
H	-0.39606200	-1.41200400	1.48778400
C	-2.59969800	-2.20342800	0.11928300
H	-3.67476000	-2.32610300	-0.04181100
H	-2.28236000	-3.03791900	0.77470600
O	-4.08731600	0.60385300	-1.28465700
H	-5.62337800	-0.76121700	-0.89809000
H	-3.69079400	0.39188000	-2.14062200
O	-6.01593800	-1.41493100	-0.29777400
H	-5.48593400	-1.22636100	0.52277800
H	-0.86274900	3.80779200	-1.09101500
O	-1.28570300	3.90637800	-0.22887600
H	-2.23114200	3.70041500	-0.40912400
H	-4.00073000	2.36087500	-0.97330000
O	-3.95293300	3.33079600	-0.80142300
H	-4.09295100	3.73765300	-1.66628200
C	-1.84630000	-2.34532100	-1.21368700
H	-1.95070300	-3.36521300	-1.60704100
H	-2.30410600	-1.68556900	-1.96262900
C	-0.06654200	-0.62128600	-0.45185400
C	-0.35178700	-2.01551000	-1.07897900
H	0.12069200	-2.09652700	-2.06474700
H	0.12308600	-2.77761900	-0.44481200
C	-0.43563500	0.69000800	1.71004800
H	-0.66061100	1.60282200	1.14612400
H	-1.01185500	0.75243300	2.63793700
C	1.05197500	0.63029200	2.07453100
H	1.18094200	-0.07108500	2.91248100
H	1.39585900	1.60191200	2.45101200
C	1.44555300	-0.42985300	-0.22143100
C	2.36975000	-0.80691900	-1.21260000
C	1.95331700	0.18445600	0.93913700
C	3.73931000	-0.60797300	-1.06661900
H	2.01273100	-1.26564400	-2.12944000
C	3.33803600	0.37660900	1.07188700
C	4.25492600	-0.00942200	0.09285700
H	4.40740900	-0.91887700	-1.86571600
H	3.70821000	0.85070600	1.98015800
C	5.74829600	0.22467200	0.27990600
H	5.88148300	0.66836400	1.27515700
C	6.30448800	1.22516200	-0.75027800
H	5.77034900	2.17967600	-0.70367900
H	7.36653100	1.42058100	-0.56543200
H	6.20964500	0.83765000	-1.77077500
C	6.54460900	-1.09413000	0.24965600
H	7.60729600	-0.90913000	0.44046500
H	6.17980200	-1.79485500	1.00739700
H	6.46203700	-1.58302500	-0.72711900
C	-0.50956800	0.49600700	-1.42788100
H	-0.33436600	1.49198500	-1.01220600
H	0.05002500	0.42192300	-2.36637000
H	-1.57075600	0.43353400	-1.66714000

MC1c

-2 1

C	-2.13902100	-2.30584600	-1.27981800
O	-3.08433600	-3.07173200	-1.12780700
H	-1.51130600	-2.40874200	-2.18847700
C	-1.82291700	-1.15124500	-0.37937800
H	-2.47253300	-0.34746400	-0.80096300
C	-0.38520800	-0.58970600	-0.46565200
H	-0.44379300	0.35518900	0.09572800
C	-2.24317500	-1.35934400	1.08860600
H	-3.23432300	-1.81649500	1.13120400
H	-2.34637200	-0.36827600	1.54505900
C	-3.86754700	1.78275600	-0.20046100
O	-4.74357300	1.06632600	0.44851200
O	-3.39599100	1.35875100	-1.33093800
O	-3.47591500	2.92166400	0.29866800
H	-5.15716900	-0.51343200	0.02530500
H	-4.42054200	3.18561500	1.85015900
H	-2.00486900	2.48506800	-1.75239600
O	-5.08759700	2.84221600	2.48995600
H	-5.21840900	2.01168400	1.97687600
O	-5.49788300	-1.44659100	-0.08790300
H	-4.74392100	-1.93952500	-0.44206700
O	-1.45244600	3.25743500	-1.48310100
H	-2.00029700	3.42794300	-0.68050200
C	0.68503800	-1.44419000	0.27667100
C	-1.21215100	-2.17943100	1.87348000
H	-1.49304600	-2.21196200	2.93328300
H	-1.22505800	-3.22069300	1.52736500
C	0.20866900	-1.60178400	1.74961100
H	0.23551300	-0.61358800	2.22842800
H	0.90297000	-2.23767200	2.30945800
C	0.87211000	-2.84820100	-0.35325900
H	1.21605700	-2.78721800	-1.38928500
H	1.62543700	-3.41141800	0.20705300
H	-0.04836000	-3.43947400	-0.33834300
C	2.04349500	-0.71766200	0.20421000
C	0.07848400	-0.19593700	-1.87369700
H	0.36397000	-1.07506500	-2.46306300
H	-0.73858200	0.29793200	-2.40974000
C	1.26484600	0.76186500	-1.74627900
H	1.74227700	0.92581600	-2.72012500
H	0.87226700	1.73953400	-1.43203900
C	2.30871500	0.28629600	-0.74987700
C	3.08733600	-1.08767000	1.07079100
C	3.57946800	0.88305000	-0.78303700
C	4.34095400	-0.48620000	1.02149600
C	4.61116600	0.52373600	0.08563300
H	2.92005300	-1.87154200	1.80334800
H	5.11224100	-0.81028000	1.71545100
H	3.76528300	1.65702700	-1.52648900
C	5.97511200	1.19569000	-0.00062300
H	5.90637200	1.96116400	-0.78460000
C	7.07498000	0.20236200	-0.42115400
H	8.03685200	0.71491000	-0.53212000

H	7.20296900	-0.58768800	0.32718000
H	6.83225800	-0.27623900	-1.37537600
C	6.35172300	1.91028700	1.31056100
H	7.30781700	2.43402000	1.20295300
H	5.59119800	2.64501400	1.59374600
H	6.45449800	1.19836700	2.13694600

TSc

1 imaginary frequencies = -1185.1651

-2 1

C	-2.26404100	-2.05400200	-1.38586400
O	-3.25452100	-2.82498900	-1.35157300
H	-1.73961200	-1.94824500	-2.36066800
C	-1.80051800	-1.18512500	-0.35126300
H	-2.54414500	-0.07795200	-0.67598300
C	-0.38623500	-0.59190200	-0.43854900
H	-0.42230400	0.30779300	0.19842900
C	-2.18279600	-1.51626400	1.09244800
H	-3.16634100	-1.99074200	1.12228400
H	-2.28113800	-0.57952700	1.65871100
C	-3.76464200	1.65281400	-0.03072100
O	-4.55065400	0.92087200	0.66867500
O	-2.97223200	1.09018400	-0.93848600
O	-3.70756800	2.93013100	0.10062700
H	-5.07089500	-0.72317400	0.21643700
H	-4.93009500	3.38234000	1.48053700
H	-1.57667100	2.57912700	-1.66350500
O	-5.58059900	3.03927000	2.12896500
H	-5.46973800	2.10405200	1.85855800
O	-5.43437800	-1.62347800	0.04242100
H	-4.70092800	-2.07795200	-0.41556300
O	-1.45788300	3.48569700	-1.33939800
H	-2.23889600	3.48407900	-0.73115600
C	0.72118400	-1.49212200	0.20409400
C	-1.12339300	-2.39508400	1.77629300
H	-1.37028200	-2.53415900	2.83760900
H	-1.13168500	-3.39655200	1.32726300
C	0.28473300	-1.78662000	1.66768200
H	0.30907300	-0.84300600	2.23064500
H	1.00428400	-2.45578100	2.15335200
C	0.90315100	-2.82892600	-0.55710600
H	1.28745700	-2.66934700	-1.56857700
H	1.62254800	-3.46799900	-0.03365800
H	-0.03651100	-3.38164500	-0.64012300
C	2.06962100	-0.74551900	0.16897800
C	0.05754800	-0.08900000	-1.81876300
H	0.35146500	-0.92037600	-2.46962100
H	-0.77189200	0.42118100	-2.31895500
C	1.23293000	0.87478200	-1.63801800
H	1.68815600	1.12626300	-2.60411700
H	0.84179500	1.82077800	-1.23480600
C	2.30449300	0.33659800	-0.70425600
C	3.13570000	-1.16716700	0.98373600
C	3.56631100	0.95265000	-0.71374200
C	4.37974400	-0.54398100	0.96162700

C	4.61921300	0.54089800	0.10508900
H	2.99322700	-2.00962800	1.65365900
H	5.16809900	-0.90928800	1.61482800
H	3.72757600	1.78639700	-1.39587000
C	5.97018800	1.24242900	0.05570800
H	5.88988200	2.04084000	-0.69349000
C	7.09444000	0.29303300	-0.39954100
H	8.04461200	0.83177400	-0.48379500
H	7.23794500	-0.52399700	0.31609300
H	6.86759500	-0.15107000	-1.37404500
C	6.32186800	1.90488100	1.40108700
H	7.27083100	2.44741100	1.32854100
H	5.54687900	2.61463000	1.70762500
H	6.42540800	1.15803600	2.19599500

MC2c

-2 1

C	-2.49786000	-2.06769900	-1.41995000
O	-3.62583400	-2.70086200	-1.41144100
H	-2.03514600	-1.89991700	-2.41712100
C	-1.79365500	-1.54626700	-0.35390500
H	-2.79134300	0.24893000	-0.77752800
C	-0.44843000	-0.85113100	-0.46318600
H	-0.53220800	0.08005100	0.12848700
C	-2.21541600	-1.80013700	1.07726400
H	-3.16099500	-2.34950200	1.09202800
H	-2.40789000	-0.84615100	1.59655500
C	-3.54539200	1.85741600	0.03389800
O	-4.22210400	1.15255300	0.83163800
O	-2.84181500	1.22105700	-0.96031600
O	-3.43350800	3.11656200	0.03098600
H	-4.99316400	-0.53604700	0.32086500
H	-4.58026100	3.71931400	1.51490200
H	-1.26189100	2.80135600	-2.01255000
O	-5.17344200	3.41631400	2.22835700
H	-5.08784700	2.46250900	2.04140400
O	-5.45836000	-1.35981600	0.08329900
H	-4.78576200	-1.86888800	-0.43926100
O	-1.17915000	3.62681200	-1.51740100
H	-1.95402200	3.54440400	-0.91394600
C	0.69980200	-1.65062400	0.25126900
C	-1.12176900	-2.55612100	1.85440800
H	-1.38290100	-2.62820100	2.91956200
H	-1.05639400	-3.58712400	1.48318200
C	0.24960200	-1.87221000	1.72371100
H	0.20622900	-0.89128200	2.21848600
H	0.99774300	-2.46003900	2.26828800
C	0.95088800	-3.01893000	-0.42919600
H	1.39668800	-2.89797800	-1.42108400
H	1.64330900	-3.62430800	0.16640200
H	0.01824500	-3.57642500	-0.54811100
C	2.01262300	-0.84724200	0.19395800
C	0.00075700	-0.39664200	-1.85749600
H	0.33218200	-1.24886500	-2.46247700
H	-0.83606300	0.05966700	-2.39554800

C	1.13162200	0.62512200	-1.71287500
H	1.59063600	0.84168000	-2.68597300
H	0.69724000	1.57650800	-1.37004500
C	2.21244300	0.19281000	-0.73692000
C	3.08561300	-1.17433900	1.04257200
C	3.44549300	0.86397300	-0.76701200
C	4.30167500	-0.49922300	0.99815500
C	4.50466400	0.54632600	0.08520500
H	2.97185800	-1.98387000	1.75727800
H	5.09677600	-0.79341200	1.67877200
H	3.57896700	1.66476200	-1.49331400
C	5.82385100	1.30363600	0.01066700
H	5.71361900	2.06345400	-0.77414600
C	6.99099500	0.38475000	-0.39668000
H	7.91781900	0.95960900	-0.50033200
H	7.16436400	-0.39313800	0.35513200
H	6.78984200	-0.11141400	-1.35154100
C	6.13878300	2.04099400	1.32572700
H	7.06081300	2.62491900	1.23026300
H	5.32954800	2.72547900	1.59946800
H	6.27598300	1.33619200	2.15324500

MC1d

-2 1

C	1.77160600	3.08932800	0.54622800
O	2.88643500	3.38268900	0.94959300
H	0.97596500	3.86755400	0.53836100
C	1.38733100	1.75004600	-0.02789900
H	2.01341400	0.98636900	0.45442000
C	-0.11890300	1.47309300	0.16254500
H	-0.65354300	2.28911900	-0.35342700
C	1.76045100	1.79636600	-1.53751500
H	2.84580400	1.91024000	-1.62173200
H	1.29514300	2.67957000	-1.99969100
C	4.27288300	-1.29557000	0.42526700
O	4.96496400	-0.70599700	-0.51348700
O	3.45850400	-0.60206100	1.15445000
O	4.41312400	-2.57626100	0.61402200
H	5.00163400	0.95538800	-0.47158000
H	5.69800400	-3.15826500	-0.55488800
H	2.61154100	-1.77626900	2.23268400
O	6.33592800	-2.93850500	-1.27407300
H	6.07246100	-1.99080700	-1.28000900
O	5.10004200	1.95256200	-0.52080500
H	4.42720900	2.27832600	0.09587200
O	2.46546000	-2.70906400	2.52590800
H	3.15195000	-3.05322000	1.91135500
C	1.28202500	0.53624600	-2.26693500
H	1.46507300	0.64747800	-3.34271200
H	1.88055300	-0.32143400	-1.94049300
C	-0.58354500	0.15354800	-0.52861000
C	-0.20940100	0.25823200	-2.03214500
H	-0.48246600	-0.66298000	-2.55689700
H	-0.80256700	1.06452800	-2.48527900
C	0.08768100	-1.10242300	0.09260600

H	1.17473100	-1.01798200	0.17017800
H	-0.29267100	-1.29802100	1.09907700
H	-0.15065800	-1.98299900	-0.51461600
C	-2.10554300	-0.00073600	-0.34292500
C	-0.56957200	1.50737900	1.62867800
H	-0.15824400	2.38350500	2.14458900
H	-0.18148900	0.63192700	2.16134300
C	-2.79048300	0.62440800	0.71772300
C	-2.09882400	1.55497200	1.70029000
H	-2.44519300	1.33494400	2.71719800
H	-2.42888100	2.58287400	1.49143400
C	-2.84942300	-0.83428600	-1.19670000
C	-4.17009300	0.41271700	0.86921300
C	-4.91029300	-0.40388500	0.01265200
C	-4.21689500	-1.03287900	-1.03197600
H	-2.35033900	-1.34726900	-2.01240400
H	-4.74406000	-1.68406800	-1.72441200
H	-4.68079900	0.90903000	1.69331500
C	-6.40732100	-0.59792100	0.21513500
H	-6.69240800	-0.00175500	1.09197800
C	-6.76080200	-2.06573900	0.52017600
H	-7.83273000	-2.17033500	0.72087700
H	-6.51587800	-2.71826900	-0.32541200
H	-6.21363500	-2.43076400	1.39536400
C	-7.22000300	-0.07387900	-0.98416800
H	-6.99054700	-0.63736900	-1.89553900
H	-8.29465700	-0.17160500	-0.79480600
H	-7.00362500	0.98155100	-1.17885300

TSd

1 imaginary frequencies = -1283.7215

-2 1

C	-2.18897100	-2.39908900	1.14544600
O	-3.21645300	-3.09738800	0.96561900
H	-1.76690400	-2.40181200	2.17202700
C	-1.56317800	-1.51674400	0.20832900
H	-2.37733000	-0.40399700	0.57626500
C	-0.05747200	-1.21418000	0.42696500
H	0.49213300	-2.13773500	0.16091200
C	-1.88924200	-1.82937600	-1.25686200
H	-2.96853900	-1.92901800	-1.38518400
H	-1.45030900	-2.80632000	-1.53100900
C	-3.89987900	1.19194000	0.24226900
O	-4.38320100	0.58839000	-0.78038000
O	-3.01713600	0.56589800	1.02172200
O	-4.22542500	2.38962000	0.56297900
H	-4.97008500	-1.07840900	-0.64580900
H	-5.49269400	2.97274400	-0.75355700
H	-2.20711300	1.92641100	2.37140600
O	-5.97122500	2.71331100	-1.56771800
H	-5.57179300	1.81901100	-1.60878300
O	-5.30129300	-2.00588100	-0.60741800
H	-4.59906800	-2.44962000	-0.09159700
O	-2.37827800	2.86965600	2.53131000
H	-3.09939200	2.95029900	1.86332000

C	-1.34009800	-0.76978200	-2.21254800
H	-1.51862900	-1.07003800	-3.25396500
H	-1.88951600	0.16583200	-2.05915700
C	0.52456200	-0.13356700	-0.55519100
C	0.16287200	-0.55098300	-2.00522600
H	0.51794800	0.20441800	-2.71511100
H	0.69645000	-1.48181800	-2.24663700
C	-0.03271000	1.28990500	-0.28698400
H	-1.12177700	1.30262200	-0.24721000
H	0.33099000	1.69477300	0.66174900
H	0.30246500	1.97182300	-1.07691500
C	2.05344000	-0.05296100	-0.36907200
C	0.36440700	-0.87179700	1.86636200
H	-0.09617800	-1.54592800	2.59279900
H	0.02197800	0.13502400	2.12904400
C	2.66915900	-0.40545800	0.84793100
C	1.88587900	-0.98145600	2.01287400
H	2.21791400	-0.50915700	2.94597000
H	2.15323800	-2.04408300	2.11102600
C	2.88009200	0.45251400	-1.38873000
C	4.05721300	-0.25421400	0.99328600
C	4.87667200	0.24035600	-0.02256600
C	4.25560800	0.59501400	-1.22911900
H	2.44152100	0.74916200	-2.33576400
H	4.84603600	0.98874500	-2.05262400
H	4.50973600	-0.53551500	1.94350500
C	6.37910100	0.38441500	0.18148000
H	6.59534500	0.06735300	1.21005000
C	6.84161800	1.84665500	0.04079500
H	7.91410200	1.93455400	0.24616700
H	6.66545300	2.22219000	-0.97315600
H	6.30703100	2.50012000	0.73761700
C	7.17470900	-0.53652200	-0.76297200
H	7.00887500	-0.26627000	-1.81174200
H	8.24941900	-0.45900100	-0.56481200
H	6.88007300	-1.58322900	-0.63652800

MC2d

-2 1

C	-2.21105500	-2.19975400	1.30216500
O	-3.37093400	-2.77419900	1.24114900
H	-1.86658700	-1.93166000	2.32244100
C	-1.34711400	-1.86606100	0.27839000
H	-2.87937800	-0.09118600	0.73301600
C	0.10421400	-1.44903200	0.48824400
H	0.72345000	-2.32741300	0.20918100
C	-1.65666000	-2.27325500	-1.14691200
H	-2.72772700	-2.46411000	-1.25545500
H	-1.13739800	-3.21984800	-1.39744800
C	-4.13257200	1.30143200	0.18213600
O	-4.39124300	0.65589300	-0.87001600
O	-3.27657900	0.72974600	1.10072900
O	-4.56533300	2.43893600	0.51300800
H	-4.91139500	-1.18142400	-0.79302600
H	-5.73342500	2.97890400	-0.98290800

H	-2.26751000	2.45435100	2.38701400
O	-6.08497600	2.70065600	-1.84924100
H	-5.63067400	1.83816900	-1.88028200
O	-5.12771200	-2.12714800	-0.69112200
H	-4.46782400	-2.41240900	-0.00299900
O	-2.62925100	3.34711900	2.30618600
H	-3.37964300	3.16609600	1.69797700
C	-1.20191500	-1.21193900	-2.15936300
H	-1.33052000	-1.57939800	-3.18703800
H	-1.84422000	-0.32791900	-2.06396000
C	0.59131400	-0.33480400	-0.51219600
C	0.26866600	-0.82238700	-1.95242500
H	0.54056200	-0.05230300	-2.68345900
H	0.90113800	-1.69507100	-2.17118500
C	-0.10243000	1.02787200	-0.26832600
H	-1.18882200	0.93958100	-0.29834400
H	0.16803300	1.45423900	0.70244200
H	0.20605000	1.74816400	-1.03453600
C	2.10625600	-0.11252400	-0.33744800
C	0.52221400	-1.09399700	1.92156400
H	0.13675400	-1.83551700	2.62713100
H	0.08534100	-0.13341600	2.21934500
C	2.76368900	-0.41603700	0.87048600
C	2.04885400	-1.05631400	2.04587200
H	2.34654200	-0.54967900	2.97283800
H	2.41937600	-2.08720200	2.14580000
C	2.87633300	0.46111300	-1.36589400
C	4.13803900	-0.15798400	0.99656100
C	4.90351800	0.39522900	-0.03069200
C	4.23938500	0.70680300	-1.22653100
H	2.40159000	0.72448000	-2.30555700
H	4.78658100	1.14402000	-2.05799800
H	4.62444000	-0.40584800	1.93939200
C	6.39839400	0.62779100	0.14556300
H	6.64154400	0.37671800	1.18635600
C	6.79228800	2.09885000	-0.07958000
H	7.86091700	2.24520100	0.11159700
H	6.59486000	2.41094000	-1.11089500
H	6.23341900	2.76547400	0.58506900
C	7.22441100	-0.30682500	-0.75947500
H	7.02724300	-0.10541000	-1.81836800
H	8.29712200	-0.16620600	-0.58642200
H	6.98341600	-1.35741200	-0.56810300

Deuterium exchange experiment of a 1:1 mixture of aldehydes 7a, b with base in deuterated solvent.

To confirm the theoretical study with regard to the observed different reactivity of aldehydes **7a, b** (β and α, respectively) in the enolization and aldol attempts, an additional experiment was carefully designed and executed successfully.

If our conclusions were correct, it should be observed a difference in the exchange rate of proton H-4 by deuterium. This difference could be detected essentially in carbon C-4 which would suffer the exchange of its proton by deuterium. Proton H-4 is contiguous to the aldehyde and, therefore, susceptible to enolization. Consequently, a small change in the chemical shift of the carbonyl group (*C*HO) in its corresponding ^{13}C NMR spectrum could also be perceived. Finally, we could also observe changes in the integration and shape of the proton of the aldehyde group in the corresponding ^{1}H NMR spectrum.

To this end, a ca. 1:1 mixture of aldehydes **7a**/**7b** (from 4(18)-epoxide), containing as a impurity a 6% of a rearranged aldehyde from 3,4-epoxide after treatment with BF_3, was subjected to deuterium exchange conditions in the presence of a base (carbonate) (see experimental section). This sample is similar to that shown in the spectrum for compounds **7** (see supporting information page S10) but from a different batch. All the solvents able to release protons (methanol and water) in this assay where substituted by their deuterated equivalents. Three aliquots after 3, 15 and 60 minutes of reaction were extracted, processed, and analyzed by ^{1}H and ^{13}C NMR spectroscopy (Fig. S9-11).

The results were as follows:

- In the ^{13}C NMR spectra (Fig. S9), it can be observed the disappearance of the signal at 52.3 ppm corresponding to the C-4 of isomer **7a** as the reaction progresses. After 60 minutes, almost all **7a** has reacted to the deuterated counterpart (appearance of a triplet of the deuterated C-4). The signal of C-4 at 51.2 ppm corresponding to the isomer **7b** remains unaltered.
- In the ^{13}C NMR spectra (Fig. S10), it can be observed the disappearance of the signal at 204.7 ppm corresponding to the carbonyl group of isomer **7a** as the reaction progresses. A new signal at 204.9 ppm is displayed corresponding to the aldehyde **7a** but with a deuterated C-4, while the signal at 205.2 ppm of isomer **7b** remains unaltered.
- In the ^{1}H NMR spectra (Fig. S11), the signal of the aldehyde group in isomer **7b** at 9.54 ppm is shown as a doublet with coupling constant J = 4.5 Hz and remains unaltered. In case of deuterium exchange at the contiguous C-4 this signal should be a singlet. For isomer **7a**, that signal is at 10.03 ppm with J = 1.2 Hz and is not helpful. This small coupling constant hinders the visualization of shape change from doublet to singlet on deuteration at C-4.

Finally, it should be noted that the integration of both signals (9.54 and 10.03 ppm) remains unaltered. This is only compatible with a deuteration preference, of the enolate of isomer **7a,** by the α-face of the molecule. Being of the same stereoselectivity observed in the aldol reaction of such enolate.

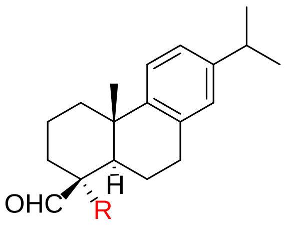

7a-H R=H
7a-D R=D

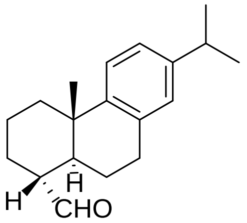

7b

52.3 ppm
(^{13}C, 4-*C*H, β-aldehyde 7a-H)

51.70 ppm
(^{13}C, 4-*C*D, triplet,
$J_{C\text{-}D}$ ~ 19 Hz, β-aldehyde 7a-D)

51.2 ppm
(^{13}C, 4-*C*H, α-aldehyde 7b)

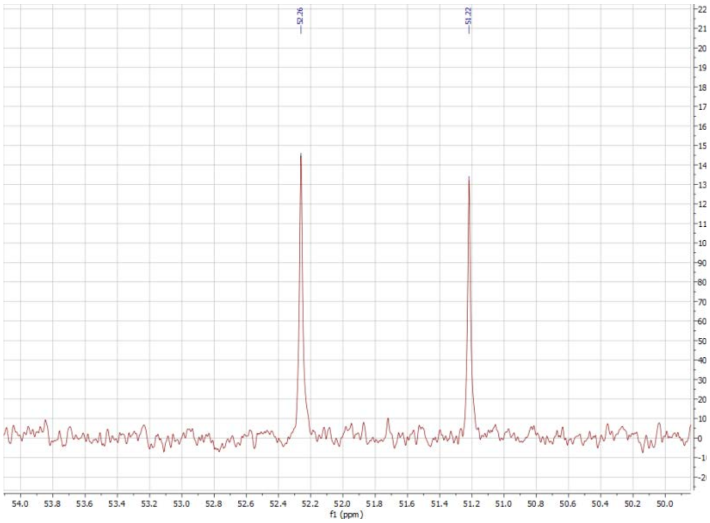

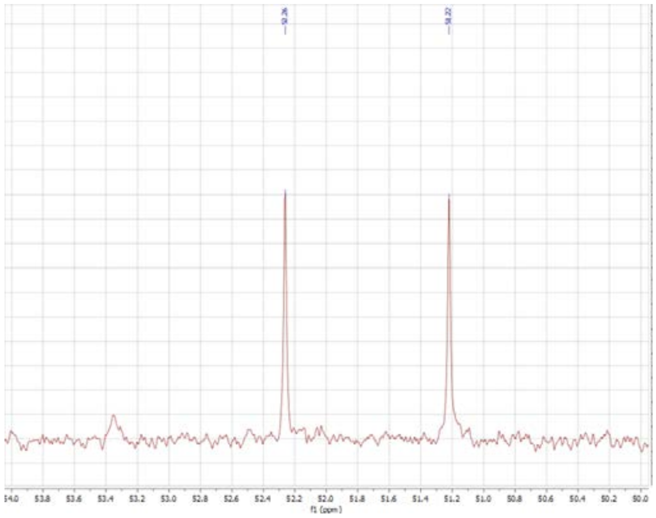

0 minutes

3 minutes

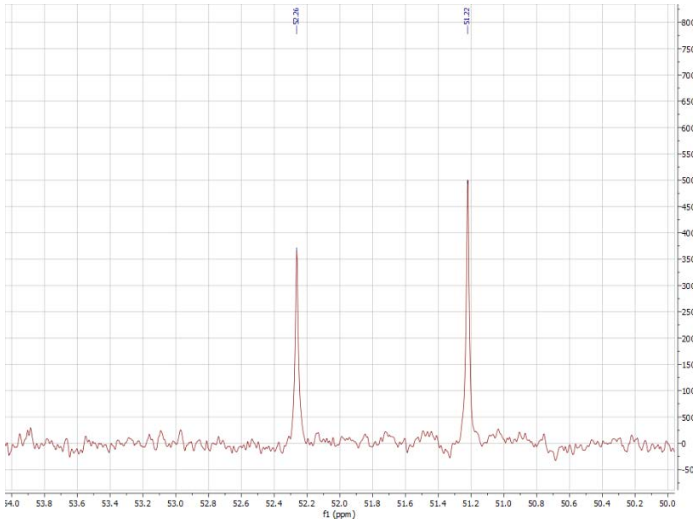

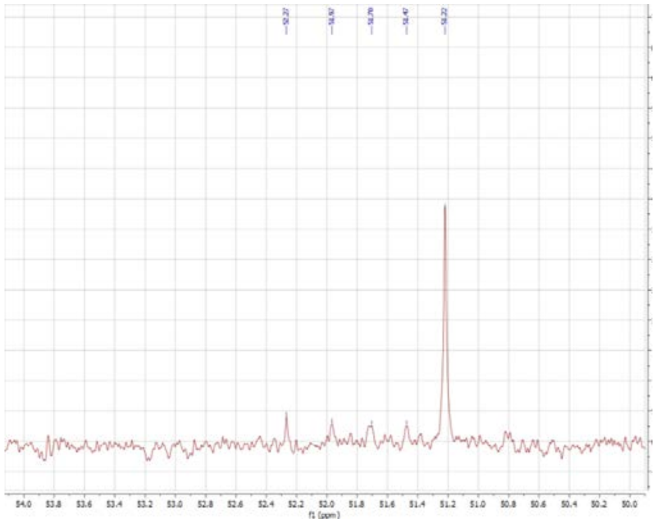

15 minutes

60 minutes

Figure S9. ^{13}C (75MHz) spectrum zoom, between 54-50 ppm, in the exchange experiment.

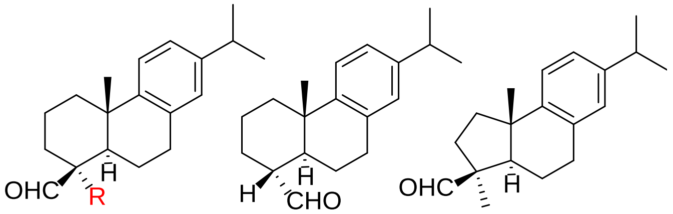

7a-H R=H
7a-D R=D

7b

rearranged aldehyde

205.2 ppm
(^{13}C, 18-*C*HO, α-aldehyde 7b-H)

204.9 ppm
(^{13}C, 19-*C*HOCD, β-aldehyde 7b-D)

204.7 ppm
(^{13}C, 19-*C*HO, β-aldehyde 7a)

203.6 ppm
(^{13}C, rearranged aldehyde)

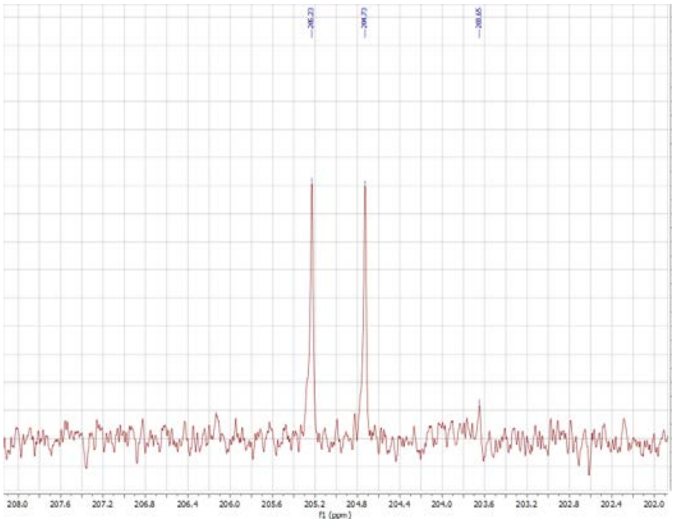

0 minutes

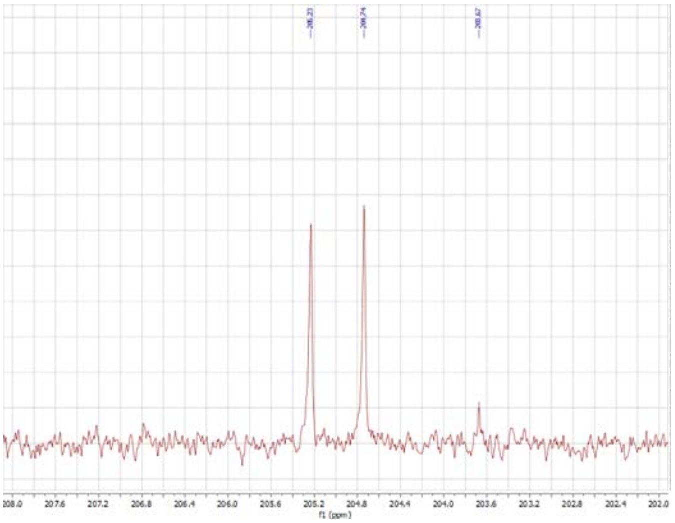

3 minutes

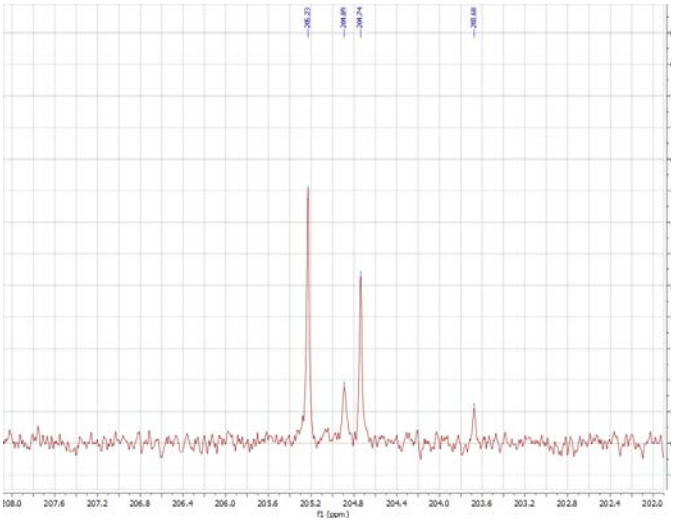

15 minutes

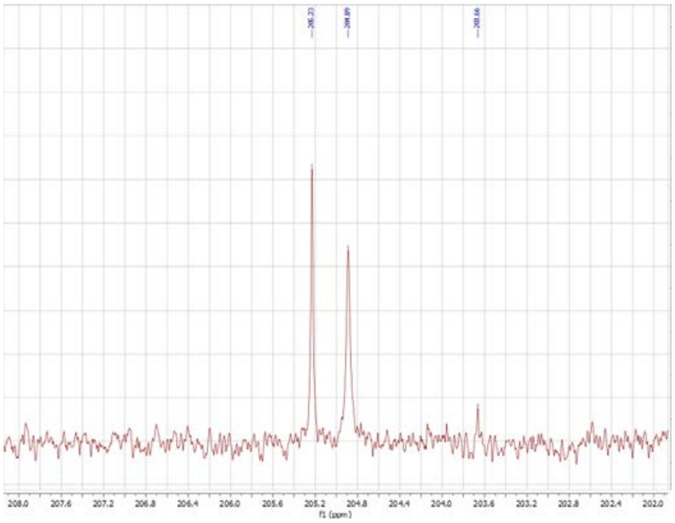

60 minutes

Figure S10. ^{13}C (75MHz) spectrum zoom, between 208-202 ppm, in the exchange experiment.

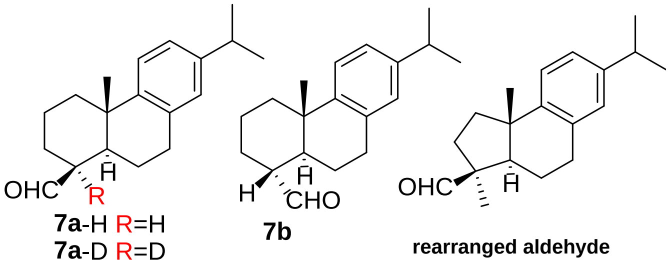

7a-H R=H
7a-D R=D

7b

rearranged aldehyde

10.03 ppm
(1H, 19-C*H*O, β-aldehyde 7a)

9.54 ppm
(1H, 18-C*H*O, α-aldehyde 7b)

9.38 ppm
(1H, rearranged aldehyde impurity from opening of mixture of epoxides, ca: 6%)

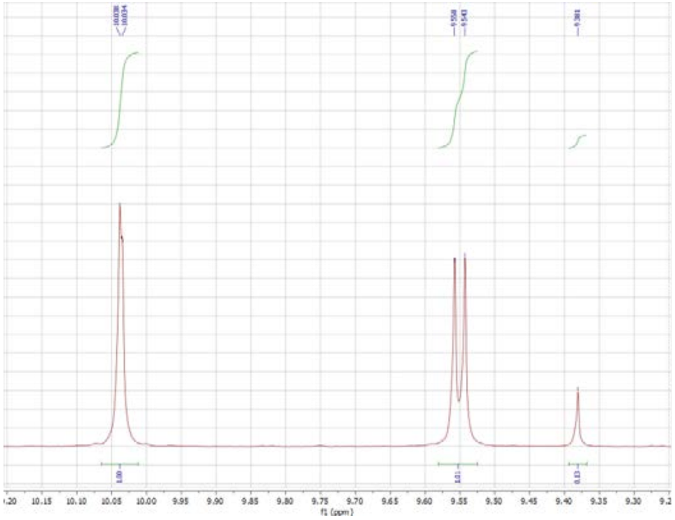

0 minutes

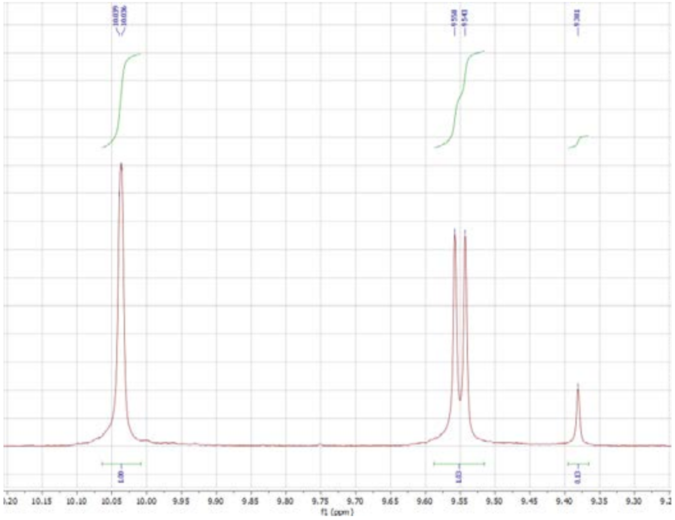

3 minutes

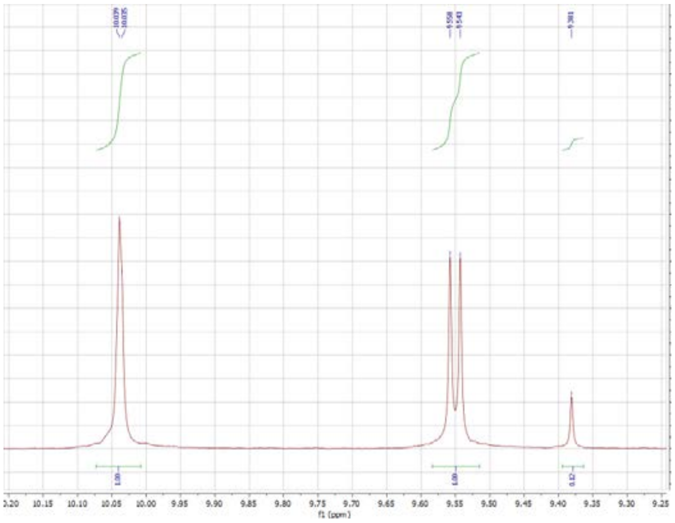

15 minutes

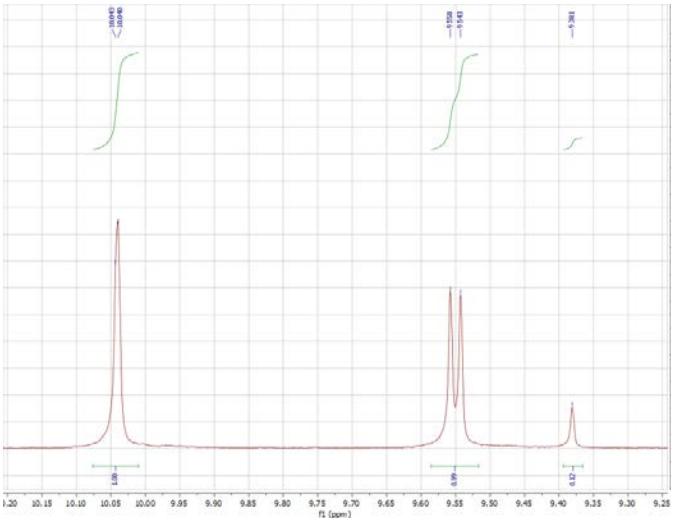

60 minutes

Figure S11. ^{1}H (300MHz) spectrum zoom, between 10.20-9.25 ppm, in the exchange experiment.
